# Hybrid ConvNeXt–Vision Mamba with Hemispheric Difference-Aware Attention and Bioinspired Optimization for Stroke Classification

**DOI:** 10.3390/biomimetics11100709

**Published:** 2026-10-04

**Authors:** Fathima Beevi Mohammed Ali, Nagarajan Santhi, Narayanan Ramasamy

**Affiliations:** 1 Department of Electronics and Communication Engineering, Noorul Islam Centre for Higher Education, Kanyakumari 629180, India; santhi@niuniv.com; 2 Department of Mechanical Engineering, Noorul Islam Centre for Higher Education, Kanyakumari 629180, India; ramasamy@niuniv.com

**Keywords:** brain stroke, computed tomography, bioinspired deep learning, hemispheric asymmetry, ConvNeXt, vision mamba, bioinspired optimization, gorilla troops optimization

## Abstract

Stroke requires rapid and accurate diagnosis, yet automated interpretation of computed tomography (CT) images remains challenging because of subtle lesion characteristics, anatomical variability, and similarities between normal and pathological tissue patterns. This study presents a hybrid ConvNeXt-Tiny–Vision Mamba framework for binary stroke classification from rendered axial CT slice images, in which ConvNeXt-Tiny extracts hierarchical local features and Vision Mamba captures long-range contextual dependencies. Motivated by the bilateral organization of the brain, an Adaptive Hemispheric Difference Attention (AHDA) module models interhemispheric feature differences through central sagittal partitioning and contralateral reflection, while Adaptive Cross-Attention Fusion (ACAF) integrates the local and asymmetry-enhanced representations. An Adaptive Lévy Flight Gorilla Troops Optimizer (ALGTO), extending Gorilla Troops Optimization with nonlinear parameter adaptation and stagnation-triggered Lévy-flight perturbation, performs hyperparameter optimization. A public Kaggle dataset of 2501 unique CT slice images from 82 filename-defined case groups was used; the images are provided as rendered files without DICOM metadata, and the correspondence of case groups to distinct patients could not be verified. All partitioning was performed at the case-group level. On the locked, single-use internal case-level test set (17 case groups), the framework achieved an accuracy of 94.12%, a weighted F1-score of 94.20%, and an area under the receiver operating characteristic curve (ROC-AUC) of 0.9697, and case-grouped five-fold cross-validation on 65 development case groups yielded a mean accuracy of 90.77 ± 3.44%, with mean precision of 89.33 ± 9.83% and F1-score of 87.92 ± 4.54%. Ablation analyses on the development set showed performance gains with the addition of Vision Mamba and AHDA, more consistent performance across folds with ACAF, and a modest further improvement with ALGTO, and Gradient-weighted Class Activation Mapping (Grad-CAM) visualization provided a qualitative illustration of the image regions influencing predictions. The framework demonstrates the potential of combining anatomically motivated attention with bioinspired adaptive optimization for CT-based stroke classification on this dataset.

## 1. Introduction

Stroke is one of the leading causes of death and permanent disability worldwide, resulting from an interruption of cerebral blood circulation that causes irreversible neuronal damage without timely intervention [1]. Stroke is broadly categorized as ischemic or hemorrhagic, and both types require rapid diagnosis to guide therapeutic decisions and improve patient survival [2].

Computed tomography (CT) is the primary imaging modality for the initial assessment of stroke owing to its rapid acquisition, wide availability, and affordability [3]. However, accurate interpretation remains challenging because stroke lesions vary considerably in size, shape, location, and intensity, while low tissue contrast, indistinct lesion boundaries, and inter-patient anatomical variation reduce diagnostic consistency, particularly in the early stages of stroke [4]. These challenges motivate the development of computer-aided diagnostic tools for automated CT analysis.

Deep learning (DL) has substantially advanced automated medical image analysis through end-to-end feature learning [5]. Convolutional neural networks (CNNs) learn hierarchical spatial representations of anatomical structures, texture patterns, and lesion boundaries; however, their limited receptive fields restrict the modeling of long-range contextual relationships, which are relevant when pathological changes must be interpreted relative to surrounding and contralateral cerebral structures. Transformer-based architectures model global dependencies through self-attention [6], although their computational cost and reliance on large annotated datasets limit clinical deployment. Vision Mamba has recently emerged as an efficient alternative for long-range contextual modeling [7]; nevertheless, global modeling alone may not preserve the fine-grained local characteristics required to identify subtle stroke-related abnormalities.

Several challenges therefore remain. First, standalone CNN- or global-context-based architectures may not fully exploit the complementary relationship between local lesion characteristics and long-range anatomical dependencies. Second, conventional attention and fusion strategies rarely incorporate the bilateral anatomical organization of the brain explicitly, although stroke-related abnormalities often manifest as differences between corresponding regions of the two hemispheres. Third, simple concatenation or static fusion may not adequately capture interactions between heterogeneous local and global representations.

Hyperparameter selection presents an additional challenge, as the performance and convergence of complex hybrid architectures depend strongly on hyperparameters, and conventional tuning methods can be computationally demanding in complex search spaces [8,9]. Bioinspired population-based metaheuristics provide an alternative by translating biological search behaviors into computational mechanisms. Gorilla Troops Optimization (GTO), inspired by the social organization and movement behavior of gorilla troops, balances global exploration and local exploitation; however, like other population-based optimizers, it may experience reduced population diversity and premature convergence during later search stages.

To address these limitations, a dual-stream ConvNeXt-Tiny–Vision Mamba framework is proposed, in which ConvNeXt-Tiny extracts hierarchical local representations and Vision Mamba captures long-range contextual dependencies. Motivated by the bilateral organization of the cerebral hemispheres, an Adaptive Hemispheric Difference Attention (AHDA) module explicitly models interhemispheric feature differences, and an Adaptive Cross-Attention Fusion (ACAF) mechanism integrates the complementary representations for classification. Furthermore, an Adaptive Lévy Flight Gorilla Troops Optimizer (ALGTO) extends GTO with nonlinear parameter adaptation and stagnation-triggered Lévy-flight perturbation to regulate the exploration–exploitation balance and preserve population diversity during hyperparameter optimization. The major contributions are as follows:

- A dual-stream ConvNeXt-Tiny–Vision Mamba architecture is developed to jointly extract local lesion characteristics and long-range anatomical dependencies from brain CT images.
- An anatomically motivated AHDA module is introduced to model interhemispheric feature differences through central sagittal partitioning and horizontal reflection of the contralateral feature representation, thereby emphasizing asymmetric characteristics associated with stroke.
- An ACAF mechanism is designed to integrate local spatial and global contextual representations through adaptive cross-attention.
- A bioinspired ALGTO is developed by extending GTO with nonlinear parameter adaptation and stagnation-triggered Lévy-flight perturbation for automated hyperparameter optimization.

The remainder of this paper is organized as follows: Section 2 reviews related work, Section 3 describes the proposed methodology, Section 4 presents and discusses the experimental results, and Section 5 concludes the paper.

## 2. Related Works

Automated brain stroke detection using DL has evolved from CNN-based classifiers to hybrid, attention-based, segmentation-based, and transformer-based approaches. Early CNN-based studies established the feasibility of CT-based stroke classification: Tursynova et al. [10] reported 80% accuracy, Abdi et al. [11] incorporated explainable AI into a CNN trained on 2501 CT images, achieving 96% precision and recall and 89.73% external accuracy, and Fontanella et al. [12] reported 72% accuracy for ischemic lesion detection on 5772 CT scans. These studies, however, relied primarily on local feature representations. Hybrid and attention-based approaches subsequently combined local and global representations. Raj et al. [13] developed StrokeViT, combining CNN-based local features with Vision Transformer-based long-range modeling, and achieved 87% slice-level and 92% patient-wise accuracy. Bajaj et al. [14] reported 92.43% accuracy with an ensemble CNN, Jena et al. [15] achieved 93.36% for hemorrhagic stroke using a DenseNet121-based CNN–BiLSTM framework, and Inamdar et al. [16] reported 92.42% accuracy using a dual-stream attention-based framework on 7842 multi-center CT images.

Segmentation-based studies have focused on lesion localization, including YOLOv10 on DWI data [17], attention-based CT-perfusion segmentation [18], a modified 3D U-Net for NCCT–DWI pairs [19], and 3D U-Net and attention-enhanced networks for MRI lesion delineation [20,21]; these approaches, however, require pixel-level annotations and modality-specific optimization. Transformer-based approaches have introduced global contextual modeling, including a Swin Transformer for multi-class intracranial hemorrhage classification [22] and a Vision Transformer for three-class multi-slice CT classification with patient-wise lesion localization, achieving 87.51% accuracy [23].

Despite these advances, several gaps remain. CNN-based approaches [10,11,12] provide limited modeling of long-range context, while hybrid and transformer-based frameworks [13,14,15,16,22,23] have not explicitly explored the interaction between complementary local and global representations or incorporated feature-level interhemispheric comparison. In particular, none of the reviewed studies explicitly compared mirrored left–right feature locations about a fixed central sagittal reference before computing interhemispheric feature differences. In addition, adaptive bioinspired hyperparameter optimization integrated with hybrid stroke-classification architectures has received limited attention. The proposed framework addresses these gaps through AHDA, ACAF, and ALGTO within a single binary stroke-classification architecture.

## 3. Materials and Methods

The proposed framework for automated stroke classification from axial CT images comprises image preprocessing, dual-stream feature learning, anatomically motivated attention, adaptive feature fusion, and bioinspired hyperparameter optimization. Duplicate images are first removed, and the remaining images are partitioned at the case-group level into training, validation, and case-independent test subsets. Following preprocessing, with online data augmentation applied only to the training subset, the images are processed by ConvNeXt-Tiny and Vision Mamba to extract local spatial and long-range contextual features, respectively. These representations are refined by the AHDA module, which uses a central sagittal reference and contralateral reflection to model interhemispheric feature differences, and are then integrated by the ACAF module for classification. ALGTO identifies the training hyperparameters used for the final model, which is evaluated once on the case-independent test set, whereas cross-validation, ablation studies, convergence and sensitivity analyses, and baseline and optimizer comparisons are conducted on the development set; Grad-CAM visualization is used for qualitative interpretation. Figure 1 illustrates the overall workflow of the proposed framework.

### 3.1. Dataset

The dataset was obtained from a publicly available Kaggle repository for brain stroke classification [24]. It comprises 2515 axial brain CT slice images in two classes, 1551 Normal and 964 Stroke, corresponding to 82 filename-defined case groups containing 19–40 slices each (median 30). The data are provided as rendered two-dimensional image files rather than raw DICOM CT data; consequently, Hounsfield unit values, display-window settings, acquisition parameters, and slice ordering are unavailable, and all processing operates on the pre-windowed 8-bit intensities of individual slices. As the repository provides no clinical metadata, images were grouped according to the case identifiers embedded in their filenames (hereafter referred to as case groups), and all images sharing a case identifier were assigned to the same subset. Whether each case group corresponds to one distinct patient or examination could not be verified from the available information. The repository does not disclose the acquisition protocol, inclusion criteria, diagnostic reference standard, annotation procedure, or stroke subtype (ischemic or hemorrhagic), as labels are provided only at the directory level. Fourteen duplicate images were excluded, yielding 2501 unique images. All images have a resolution of 650 × 650 pixels and contain identical grayscale intensities across three channels; this three-channel representation was preserved. The dataset exhibits a moderate class imbalance and variations in lesion appearance, location, intensity, and slice characteristics. Representative examples are presented in Figure 2.

### 3.2. Data Splitting

The dataset was partitioned at the case-group level, such that all images sharing a case identifier were confined to a single subset, which was verified programmatically by confirming that the pairwise intersections of the subsets’ case-identifier sets were empty. The training set comprised 57 case groups (1761 images: 1065 Normal and 696 Stroke), the validation set 8 case groups (221 images: 148 Normal and 73 Stroke), and the case-independent test set 17 case groups (519 images: 338 Normal and 181 Stroke); no resampling or class-balancing procedures were applied. Because case groups could not be verified as distinct patients, this partitioning cannot guarantee independence between subsets at the patient level. The training and validation subsets together formed the development set (65 case groups), on which all model development and comparative analyses were performed, whereas the test set was not used for any model development or comparative analysis and was evaluated only once using the final, locked model configuration. The complete split manifest, listing the case identifier, class label, and subset assignment for all 82 case groups, is provided in the Supplementary Materials (Table S1).

### 3.3. Pre-Processing

All images were resized from 650 × 650 × 3 to 224 × 224 × 3 pixels, retaining the three-channel representation for compatibility with the pretrained ConvNeXt-Tiny backbone. Contrast Limited Adaptive Histogram Equalization (CLAHE), which applies histogram equalization within local tiles with a clip limit to prevent noise over-amplification [25], was then applied to enhance local tissue contrast (clip limit = 2.0, tile grid size = 8 × 8). Given the identical intensities across channels, CLAHE was applied to a single channel and the result duplicated across all three, avoiding inter-channel discrepancies. Gamma correction with a fixed, non-optimized coefficient of γ = 1.2 was subsequently applied to moderately adjust image intensity while preserving lesion-related contrast:

(1) $$I_{\gamma }\left(x,y\right)=255{\left(I_{C}\left(x,y\right)/255\right)}^{\gamma }$$

where *I_C_*(*x*,*y*) and *I_γ_*(*x*,*y*) denote the CLAHE-enhanced and gamma-corrected pixel intensities, respectively. The intensities were finally normalized to [0, 1]:

(2) $$I_{\mathrm{norm}}\left(x,y\right)=\frac{I_{\gamma }(x,y)}{255}$$

The complete preprocessing workflow is illustrated in Figure 3.

### 3.4. Online Data Augmentation

Online data augmentation was applied only to the training set to improve generalization and reduce overfitting, while the validation and test sets remained unchanged. During each epoch, random rotation (±8°), random zoom (±15%), and random contrast adjustment (±20%) were applied, as these transformations introduce moderate, clinically plausible variation. Translation was omitted to preserve the fixed midline reference required by the AHDA module, as horizontal shifting could introduce spurious interhemispheric asymmetry into normal scans. Horizontal and vertical flipping, large-angle rotation, and elastic deformation were likewise excluded to avoid altering anatomical orientation or generating implausible samples. The adopted augmentation operations are illustrated in Figure 4.

### 3.5. Model Development

The preprocessed images are processed by a dual-stream ConvNeXt-Tiny–Vision Mamba architecture that extracts complementary local and long-range contextual representations, which are refined by AHDA, integrated by ACAF, and classified using global average pooling (GAP) and a fully connected layer.

#### 3.5.1. Hierarchical Local Feature Representation Using ConvNeXt-Tiny

ConvNeXt-Tiny extracts hierarchical local features using modern CNN design principles, including large-kernel depthwise convolution, inverted bottlenecks, Layer Normalization, and residual connections [26]. The backbone was initialized with ImageNet-1K pretrained weights, and all layers were fine-tuned. As shown in Figure 5, it comprises a patch embedding layer followed by four hierarchical stages. The input image (224 × 224 × 3) is first partitioned into non-overlapping patches by a 4 × 4 convolution with stride 4, producing a 56 × 56 × 96 embedding:

*F*_0_ = *W_p_* ∗ *I_p_* + *b_p_* (3)

where W_p_ and b_p_ are the learnable convolution kernel and bias, and F_0_ is the embedded feature map.

The four stages, comprising 3, 3, 9, and 3 ConvNeXt blocks, produce feature maps of 56 × 56 × 96, 28 × 28 × 192, 14 × 14 × 384, and 7 × 7 × 768, progressively encoding tissue boundaries and textures in early stages and lesion morphology and higher-level semantics in deeper stages. Each block applies a 7 × 7 depthwise convolutions:

(4) $$F_{i}^{\prime }=\mathrm{DWConv}(F_{i})$$

where *F_i_* is the input feature map and *DWConv*(⋅) represents the depthwise convolution operation, followed by Gaussian Error Linear Unit (GELU) activation function, pointwise transformations (1 × 1 convolutions), and Layer Normalization:

(5) $$F_{i}^{\prime \prime }={\mathrm{PW}}_{2}\left(\phi \left(PW_{1}\left(\mathrm{LN}(F_{i}^{\prime })\right)\right)\right)$$

where *LN*(⋅) denotes Layer Normalization, *PW*_1_(⋅) and *PW*_2_(⋅) represent the two pointwise linear transformations and *ϕ*(⋅) denotes the GELU activation function. Residual learning is then employed to preserve the input representation and facilitate stable gradient propagation.

(6) $$F_{i+1}=F_{i}+F_{i}^{\prime \prime }$$

where *F_i_*_+1_ represents the output feature map generated after residual aggregation.

The overall backbone is expressed as:

*F_C_* = *f_ConvNeXt_*(*F*_0_; Θ*_C_*) (7)

where *f_ConvNeXt_*(⋅) denotes the ConvNeXt-Tiny feature extraction function comprising the four sequential stages, Θ*_C_* represents the trainable network parameters. The local representation *F_C_* (14 × 14 × 384) is taken from Stage 3 rather than Stage 4 to retain the finer spatial resolution required for the hemispheric comparison performed by AHDA.

#### 3.5.2. Long-Range Contextual Feature Representation Using Vision Mamba

Vision Mamba complements the local ConvNeXt-Tiny features by modeling long-range contextual relationships across spatially distributed brain regions through selective state-space modeling [27]. The Vision Mamba (Vim) branch was trained from scratch with all layers trainable. As shown in Figure 6, it comprises patch embedding, stacked Vision Mamba blocks, selective state-space operations, gating, Layer Normalization, and residual connections. The preprocessed image *I_p_* is divided into patches by a 4 × 4 convolution with stride 4 and flattened into a sequence of 3136 tokens (56 × 56) with embedding dimension 384:

*X*_0_ = *W_e_* ∗ *PatchEmbed*(*I_p_*) + *b_e_* (8)

where *W_e_* and *b_e_* represent the learnable embedding weights and bias, respectively, and *X*_0_ (3136 × 384) denotes the embedded feature sequence.

Each of the 24 stacked blocks propagates contextual information using the continuous-time state-space formulation, with a state dimension of 16:

*h*′(*t*) = *Ah*(*t*) + *Bx*(*t*) (9)

*y*(*t*) = *Ch*(*t*) + *Dx*(*t*) (10)

where *h*(*t*) is the hidden state, *x*(*t*) the input sequence, and *A*, *B*, *C*, and *D* the state transition, input projection, output projection, and direct input mapping matrices, respectively.

To provide input-dependent selectivity, the parameters are derived from the current features:

*B_t_* = *f_B_*(*x_t_*), *C_t_* = *f_C_*(*x_t_*), *Δ_t_* = *softplus*(*f_Δ_*(*x_t_*)) (11)

where *f_B_*(·), *f_C_*(·), and *f_Δ_*(·) denote the learnable projection functions, while *B_t_*, *C_t_*, and *Δ_t_* represent the input-dependent state-space parameters at position *t*. The softplus operation ensures that the discretization step *Δ_t_* remains positive. These continuous parameters are then discretized according to the input-dependent step size:

(12) $${\bar{A}}_{t}=\exp\left(\Delta_{t}A\right),{\bar{B}}_{t}={\left(\Delta_{t}A\right)}^{\left(-1\right)}\left(\exp\left(\Delta_{t}A\right)-I\right)\Delta_{t}B_{t}$$

where ${\bar{A}}_{t}$ and ${\bar{B}}_{t}$ are the discretized state transition and input projection terms, respectively. The recurrent selective state update is:

(13) $$h_{t}={\bar{A}}_{t}h_{\left(t-1\right)}+{\bar{B}}_{t}x_{t}$$

with the corresponding contextual output:

*y_t_* = *C_t_h_t_* + *Dx_t_* (14)

where *h_t_* is the current hidden state, *h*_(*t*−1)_ denotes the preceding hidden state, *x_t_* represents the current visual feature, and *y_t_* denotes the resulting contextual output. A gating mechanism regulates the contribution of the contextual representation:

*g_t_* = *σ*(*W_g_x_t_* + *b_g_*) (15)

where *W_g_* and *b_g_* represent learnable gating parameters and *σ* denotes the sigmoid activation function; the gated output is combined with residual information and normalized. The stacked blocks are expressed overall as:

*F_M_* = *f_Mamba_*(*X*_0_;*Θ_M_*) (16)

where *f_Mamba_*(.) is the stacked Vision Mamba feature extraction function and *Θ_M_* represents its trainable parameters. The output sequence (3136 × 384) is reshaped to 56 × 56 × 384 and downsampled by adaptive average pooling to 14 × 14 × 384, matching the spatial resolution o *F_C_* for subsequent processing by AHDA.

#### 3.5.3. AHDA for Stroke-Specific Feature Enhancement

Although ConvNeXt-Tiny and Vision Mamba provide complementary local and contextual information, neither explicitly models structural asymmetry between the cerebral hemispheres. In brain CT images, ischemic and hemorrhagic lesions frequently appear as unilateral abnormalities that alter tissue density, morphology, and intensity distributions across the hemispheres [28]. AHDA therefore explicitly models feature-level interhemispheric differences to enhance stroke-relevant responses while suppressing redundant symmetric information. As illustrated in Figure 7, it comprises feature alignment, hemispheric decomposition, contralateral alignment, asymmetry estimation, adaptive attention generation, and feature recalibration.

The local feature representation *F_C_* (14 × 14 × 384, from Stage 3 of ConvNeXt-Tiny) and the global contextual feature representation *F_M_* (14 × 14 × 384, obtained from Vision Mamba via adaptive average pooling) are concatenated (14 × 14 × 768) and projected into a single feature space (14 × 14 × 384) using a 1 × 1 convolution followed by the GELU:

*F_A_* = *ϕ*(*W_a_* ∗ [*F_C_*∥*F_M_*] + *b_a_*) (17)

where ∥ represents channel-wise concatenation, *W_a_* and *b_a_* correspond to the learnable convolution kernel and bias, and *F_A_* (14 × 14 × 384) is the aligned feature representation.

AHDA assumes that the input images follow the consistent axial orientation and approximately midline-centered framing of standard head CT acquisition. The central spatial axis is therefore used as a fixed midline reference (*x_m_* = *W*/2 = 7, for spatial width *W* = 14), without per-image midline estimation, registration, or skull stripping; as the acquisition protocol of the dataset is undocumented, this centering could not be verified for each image. Consequently, the left–right comparison performed by AHDA approximates, rather than establishes, anatomical hemispheric correspondence, and its validity depends on the head being approximately centered and aligned within the image. The aligned feature map is divided at this midline into left and right hemispheric representations:

*F_L_* = Ψ*_L_*(*F_A_*),  *F_R_* = Ψ*_R_*(*F_A_*) (18)

where *F_L_* and *F_R_* denote the left and right hemispheric feature representations, respectively, while Ψ*_L_*(⋅) and Ψ*_R_*(⋅) represent the corresponding hemispheric partitioning operations. Specifically, the left hemisphere is obtained from the spatial 0 ≤ *x* < 7 (14 × 7 × 384), whereas the right hemisphere is obtained from 7 ≤ *x* < 14 (14 × 7 × 384), an equal split since *W* = 14 is even. Because the two hemispheres have opposite medial-to-lateral ordering, the right hemispheric representation is horizontally reflected before comparison:

(19) $$F_{R}^{\mathrm{ref}}=\Psi_{\mathrm{flip}}\left(F_{R}\right)$$

where Ψ_flip_(⋅) denotes horizontal reflection along the spatial width dimension, and $F_{R}^{\mathrm{ref}}$ (14 × 7 × 384) represents the horizontally reflected right-hemisphere feature representation. Consequently, a feature location at a given spatial position in *F_L_* is compared with its mirrored location relative to the image center, without a learned registration module. The absolute interhemispheric difference is then computed:

(20) $$H_{D}=\left|F_{L}-F_{R}^{\mathrm{ref}}\right|$$

where *H_D_* (14 × 7 × 384) is the hemispheric difference map, in which larger values indicate greater asymmetry between mirrored regions. GAP summarizes this map into a channel descriptor:

*z* = *GAP*(*H_D_*) (21)

where z (384 × 1) is subsequently processed by two fully connected layers with GELU and sigmoid activations to generate adaptive attention weights:

*A_H_* = *σ*(*W*_2_ ∗ *φ*(*W*_1_ ∗ *z* + *b*_1_) + *b*_2_) (22)

where *W*_1_ and *W*_2_ denote the learnable weight matrices, *b*_1_ and *b*_2_ represent their corresponding bias terms, and *A_H_* (384 × 1) denotes the adaptive hemispheric attention vector. The attention weights are broadcast spatially and applied with residual learning:

*F_H_* = *LN*(*F_A_* + *F_A_* ⊙ *A_H_*) (23)

where ⊙ denotes element-wise multiplication and *F_H_* (14 × 14 × 384) denotes the stroke-enhanced representation. The overall AHDA transformation is:

*F_H_* = *f_AHDA_*(*F_A_*; *Θ_H_*) (24)

where *f_AHDA_*(·) denotes the proposed AHDA transformation and *Θ_H_* represents the trainable parameters of the module. By comparing mirrored left–right feature locations about the image midline, AHDA translates the bilateral organization of the cerebral hemispheres into a feature-recalibration mechanism that enhances asymmetry-related responses while retaining the complementary local and contextual information from both branches.

#### 3.5.4. Adaptive Cross-Attention Fusion

The AHDA-enhanced representation must be integrated with the complementary local features of ConvNeXt-Tiny. ACAF therefore establishes adaptive interactions between *F_C_* and *F_H_* (each 14 × 14 × 384, flattened to 196 × 384) through single-head scaled dot-product cross-attention [29]. As illustrated in Figure 8, ACAF comprises feature projection, Query–Key–Value generation, cross-attention computation, feature aggregation, and feature refinement. Both representations are first projected into a common latent space:

*P_C_* = *W_C_* ∗ *F_C_* + *b_C_* (25)

*P_H_* = *W_H_* ∗ *F_H_* + *b_H_* (26)

where *W_C_* and *W_H_* denote the learnable projection matrices, *b_C_* and *b_H_* (196 × 384 each) are the bias vectors, and *P_C_* and *P_H_* are the projected feature representations.

The projected local representation generates the Query, whereas the AHDA-enhanced representation generates the Key and Value:

*Q* = *P_C_* ∗ *W_Q_* (27)

*K* = *P_H_* ∗ *W_K_* (28)

*V* = *P_H_* ∗ *W_V_* (29)

where *W_Q_*, *W_K_*, and *W_V_* (384 × 384 each) represent the learnable Query, Key, and Value projection matrices, and *Q*, *K*, *V* are each 196 × 384. The cross-attention weights are computed using scaled dot-product attention [30]:

(30) $$A_{C}=\mathrm{softmax}\left(\frac{QK^{T}}{\sqrt{d_{k}}}\right)$$

where *A_C_* (196 × 196) denotes the cross-attention weight matrix, *K^T^* represents the transpose of the Key matrix, and *d_k_* = 384 is the Key dimensionality, with the scaling factor $\sqrt{d_{k}}$ controlling the magnitude of the dot products. The attention weights are applied to the Value representation:

*F_CA_* = *A_C_* ∗ *V* (31)

where *F_CA_* (196 × 384) represents the cross-attended feature embedding.

To preserve the original local information, residual aggregation and Layer Normalization are applied:

*Z_R_* = *LN*(*P_C_* + *F_CA_*) (32)

*F_F_* = *LN*(*Z_R_* + *FFN*(*Z_R_*)) (33)

where *Z_R_* is the residual-refined representation and *F_F_* (196 × 384, reshaped to 14 × 14 × 384) is the final fused feature representation. The complete ACAF operation is expressed as:

*F_F_* = *f_ACAF_*(*F_C_*, *F_H_*; *Θ_F_*) (34)

where *f_ACAF_*(·) denotes the proposed Adaptive Cross-Attention Fusion operation and *Θ_F_* represents its trainable parameters. Through query-guided interaction, ACAF selectively integrates fine-grained local features with the contextual and interhemispheric information encoded by AHDA.

#### 3.5.5. Classification Head

Following Adaptive Cross-Attention Fusion, the resulting fused representation *F_F_* is aggregated using GAP:

*g* = *GAP*(*F_F_*) (35)

where *F_F_* ∈ ℝ^14×14×384^ denotes the reshaped fused feature representation and *g* ∈ ℝ^384^ denotes the resulting pooled feature vector. A dropout layer is then applied to reduce overfitting:

*g_d_* = *Dropout*(*g*, *p_d_*) (36)

where *g_d_* ∈ ℝ^384^ represents the regularized feature vector and *p_d_* = 0.30 is the dropout rate selected by ALGTO. A fully connected layer produces the classification logit:

*o* = *W_f_c* ∗ *g_d_* + *b_f_c* (37)

where *W_f_c* ∈ ℝ^384^ and *b_f_c* ∈ ℝ represent the learnable weight and bias and *o* ∈ ℝ denotes the resulting classification logit. The Stroke probability is obtained using the sigmoid function:

*P*(*y* = 1|*I_p_*) = 1/(1 + *exp*(−*o*)) (38)

and the class label is assigned using a threshold of 0.5:

*y_hat_* = 1, *if P*(*y* = 1|*I_p_*) ≥ 0.5; 0, *otherwise* (39)

where *y_hat_* = 1 represents Stroke and *y_hat_* = 0 represents Normal.

### 3.6. Proposed Adaptive Lévy Flight Gorilla Troops Optimizer

Hyperparameters such as the learning rate, weight decay, batch size, and dropout rate strongly influence the convergence and predictive performance of the proposed framework, and their interactions create a nonlinear search space in which manual tuning, grid search, and random search become computationally demanding. Conventional GTO offers a bioinspired population-based search mechanism for such spaces; however, its candidate solutions may concentrate around locally optimal regions as population diversity decreases, with no explicit mechanism to respond when improvement stagnates. ALGTO therefore extends GTO with nonlinear parameter adaptation, which regulates the exploration–exploitation transition across iterations, and stagnation-triggered Lévy-flight perturbation, which introduces controlled long-range movement when the best solution fails to improve.

#### 3.6.1. Hyperparameter Search Space

Each candidate gorilla represents a hyperparameter configuration of the proposed framework. Optimization is restricted to four training and regularization hyperparameters, summarized with their search ranges in Table 1, while the network architecture remains fixed.

Each candidate solution at iteration t is represented as:

(40) $$X_{i}^{t}=\left[x_{\left(i,1\right)}^{t},x_{\left(i,2\right)}^{t},\ldots ,x_{\left(i,d\right)}^{t}\right]$$

where $X_{i}^{t}$ denotes the i^th^ candidate solution at iteration *t*, $x_{i,j}^{t}$ is the value of the j^th^ hyperparameter, and *d* denotes the optimization dimensionality (here, d = 4). The candidate hyperparameter vector is therefore:

(41) $$X_{i}^{t}=\left[\eta_{i}^{t},B_{i}^{t},\lambda_{i}^{t},p_{\left(d,i\right)}^{t}\right]$$

where $\eta_{i}^{t}$ is the learning rate, $B_{i}^{t}$ is the batch size, $\lambda_{i}^{t}$ denotes the weight decay, and $p_{\left(d,i\right)}^{t}$ represents the dropout rate associated with the i^th^ candidate solution at iteration *t*.

To maintain feasible configurations, each decision variable is constrained by its predefined bounds:

(42) $$LB_{j}\leq x_{\left(i,j\right)}^{t}\leq UB_{j},\;\;j=1,2,\ldots ,d$$

where *LB_j_* and *UB_j_* denote the lower and upper bounds of the j^th^ hyperparameter; batch size is mapped to the nearest permissible value in {8, 16, 32}. All remaining settings, including the ConvNeXt drop-path rate, Vision Mamba block count, hidden and state dimensions, optimizer, learning-rate scheduler, and epoch count, remain fixed. Architectural hyperparameters were excluded from the search for two reasons: altering the ConvNeXt-Tiny architecture would discard its ImageNet-1K pretrained initialization, and, given the limited dataset size, expanding the search space would substantially increase its dimensionality relative to the available evaluation budget and the risk of poorly generalizing configurations.

#### 3.6.2. Fitness Function

For each candidate $X_{i}^{t}$, the corresponding hyperparameters are assigned to the framework, which is trained on the training subset and evaluated on the validation subset; the case-independent test subset is excluded from optimization to prevent information leakage. Validation loss, computed using Binary Cross-Entropy, serves as the fitness criterion, with lower values indicating better configurations:

(43) $$\mathrm{Fitness}\left(X_{i}^{t}\right)=L_{\mathrm{val}}\left(X_{i}^{t}\right)$$

where $L_{\mathrm{val}}\left(X_{i}^{t}\right)$ denotes the validation loss obtained using the configuration represented by $X_{i}^{t}$. The candidate with the minimum validation loss at each iteration is retained as the current best solution:

(44) $$X_{\mathrm{best}}^{t}=\mathrm{argmi}n_{\left(X_{i}^{t}\right)}\mathrm{Fitness}\left(X_{i}^{t}\right)$$

where $X_{\mathrm{best}}^{t}$ represents the best hyperparameter configuration identified up to iteration *t*.

#### 3.6.3. Conventional Gorilla Troops Optimizer Search Mechanism

GTO is a bioinspired population-based algorithm that abstracts the social and movement behaviors of gorilla troops into exploration and exploitation strategies [31]. Each gorilla represents a candidate hyperparameter configuration, and the candidate with the minimum validation loss is regarded as the silverback. During exploration, candidates perform stochastic movements to investigate new regions of the search space, whereas during exploitation they move toward the silverback to refine promising regions:

(45) $$X_{i}^{t+1}=\left\{\begin{array}{ll} E\left(X_{i}^{t},X_{r}^{t};C_{t},L_{t}\right), & \mathrm{exploration}\\ P\left(X_{i}^{t},X_{\mathrm{best}}^{t};C_{t},L_{t}\right), & \mathrm{exploitation} \end{array}\right.$$

where $X_{r}^{t}$ is a randomly selected candidate, *E*(⋅) and *P*(⋅) denote the conventional GTO exploration and exploitation operators, respectively, while *C_t_* and *L_t_* control the magnitude and transition of the search. Because these coefficients decay in a fixed manner regardless of optimization progress, population diversity may decrease without any mechanism to respond to stagnation, motivating the two adaptive mechanisms of ALGTO.

#### 3.6.4. Nonlinear Parameter Adaptation

ALGTO adjusts the search-control coefficient nonlinearly according to optimization progress:

(46) $$C_{t}=C_{\max}\left[1-{\left(\frac{t}{T_{0}}\right)}^{\alpha }\right]$$

where *C_max_* represents its initial maximum value, *T*_0_ denotes the maximum number of optimization iterations, and represents the nonlinear adaptation exponent. For *α* > 1, the coefficient remains high during early iterations and decreases more rapidly later, supporting broad exploration initially and stronger exploitation as optimization advances. The adapted coefficient is incorporated into the operators of Equation (45).

#### 3.6.5. Stagnation-Triggered Lévy-Flight Perturbation

To escape locally optimal regions, ALGTO applies Lévy-flight perturbation only when the best fitness fails to improve sufficiently over consecutive iterations. Stagnation is tracked as:

(47) $$S_{t}=\left\{\begin{array}{cc} S_{t-1}+1, & \left|f_{\mathrm{best}}^{t}-f_{\mathrm{best}}^{t-1}\right|<\varepsilon \\ 0, & \mathrm{otherwise} \end{array}\right.$$

where *S_t_* denotes the stagnation counter, $f_{\mathrm{best}}^{t}$ and $f_{\mathrm{best}}^{t-1}$ represent the best fitness values at two successive iterations, and ε denotes the minimum improvement tolerance. When *S_t_* reaches a predefined stagnation threshold *S_max_*, Lévy-flight perturbation is activated. A Lévy-flight step is subsequently generated as:

(48) Levy(β)=uv1β

where *u* and *v* are random variables of the Lévy-flight formulation and *β* controls the heavy-tailed step distribution, producing predominantly local movements with occasional long-range jumps.

#### 3.6.6. Integrated ALGTO Update and Optimal Hyperparameter Selection

The complete ALGTO update is:

(49) $$X_{i}^{t+1}=\left\{\begin{array}{cc} \mathrm{GTO}\left(X_{i}^{t},X_{\mathrm{best}}^{t};C^{t}\right), & S_{t}<S_{\max}\\ X_{i}^{t}+\rho \mathrm{Levy}\left(\beta \right)⊙\left(X_{i}^{t}-X_{\mathrm{best}}^{t}\right), & S_{t}\geq S_{\max} \end{array}\right.$$

where *ρ* represents the Lévy-flight scaling factor and ⊙ denotes element-wise multiplication. After each update, candidates are constrained to the search bounds and the batch size is mapped to the nearest permissible value. The configuration with the minimum validation loss across the optimization process is selected as the final solution:

(50) $$X^{*}=\arg\underset{X}{\min}L_{\mathrm{val}}\left(X\right)$$

where X* is subsequently used to train the final classification framework. The complete procedure is summarized in Algorithm 1.

**Algorithm 1.** ALGTO for Hyperparameter Optimization**Input:** Hyperparameter search space, population size *N_p_*, maximum iterations *T_o_*, nonlinear adaptation parameters *C_max_* and *α*, stagnation tolerance *ϵ*, stagnation threshold *S_max_*, Lévy exponent *β*, and Lévy scaling factor *ρ*
**Output:** Best identified hyperparameter vector X*
Randomly initialize a population of *N_p_*, candidate solutions within the predefined search bounds.Map each candidate *X_i_* to the hyperparameter vector [*η*,*B*,*λ*,*p_d_*].**For** each candidate:Assign the corresponding hyperparameters to the proposed framework.Train the model using the training set.Evaluate the trained model using the validation set.Compute the fitness using validation loss *L_val_*.Select the candidate with the minimum validation loss as the initial silverback *X_best_*.Initialize the iteration counter *t* = 1 and stagnation counter *S_t_* = 0.**While** *t* < *T_o_*:Compute the nonlinear adaptive control coefficient *C^t^*.Update candidate positions using the conventional GTO exploration and exploitation mechanisms governed by *C^t^*.Enforce the predefined search boundaries and map the batch size to the nearest valid value in {8, 16, 32}.Evaluate the validation loss of the updated candidate solutions.Update *X_best_* if a candidate produces a lower validation loss.Evaluate the improvement in the best fitness.**If** the fitness improvement is less than *ϵ*, increment *S_t_*; **otherwise**, reset *S_t_* = 0.**If** *S_t_* ≥ *S_max_*:Generate a Lévy-flight step using Levy(*β*).Apply the Lévy-flight perturbation to the candidate solution.Enforce the search boundaries.Evaluate the perturbed candidate and retain it if fitness improves.Reset the stagnation counter (*S_t_* = 0).**Set *t* = *t* + 1.**
**End While**
**Return** the best hyperparameter vector: $X^{*}=\arg\underset{X}{\min}L_{\mathrm{val}}\left(X\right)$


#### 3.6.7. Computational Complexity Analysis

The computational cost of ALGTO is dominated by the repeated training and validation of candidate configurations:

(51) $$O(N_{p}T_{o}C_{\mathrm{tv}})$$

where *N_p_* represents the population size, *T_o_* the maximum optimization iterations, and *C_tv_* the computational cost of one training and validation cycle. The ALGTO parameter values used in this study are summarized in Table 2.

The additional operations introduced by ALGTO, namely nonlinear adaptation, stagnation detection, boundary handling, and Lévy-flight perturbation, are low-cost scalar or vector computations relative to model training, and Lévy-flight perturbation is activated only upon stagnation. As ALGTO is applied only during offline model development, it introduces no additional computation during inference. The ALGTO workflow is illustrated in Figure 9.

Figure 10 distinguishes the offline hyperparameter optimization stage from final model training. ALGTO proposes candidate configurations for the fixed dual-stream architecture, the resulting validation loss guides subsequent candidate selection, and the optimal configuration is then used for final training and a locked, single-use case-independent test evaluation.

### 3.7. Training Strategy

The final framework was trained using the hyperparameter configuration identified by ALGTO, with the AdamW optimizer and a cosine annealing learning-rate schedule. Binary Cross-Entropy (BCE) loss was used as the training objective:

(52) $$L_{\mathrm{BCE}}=-\frac{1}{N}\sum_{i=1}^{N}\left[y_{i}\log\left({\hat{y}}_{i}\right)+\left(1-y_{i}\right)\log\left(1-{\hat{y}}_{i}\right)\right]$$

where *N* is the number of samples, *y_i_* represents the ground-truth binary label, and ${\hat{y}}_{i}$ denotes the predicted probability for the corresponding sample. Unweighted BCE was adopted because the moderate class imbalance of the dataset (approximately 1.6:1, Normal to Stroke) does not typically warrant class weighting or Focal Loss, which are generally applied under more severe imbalance. As BCE is computed at the slice level, case groups with more slices exert proportionally greater influence on training; given slice counts of 19–40 per case group (median 30), the largest case group contributed approximately 2.1 times as many training samples as the smallest. Case-balanced sampling and case-level loss aggregation were not applied, given the moderate range of slice counts across case groups. Training was performed for 50 epochs, and the model state with the best validation performance was retained. After the final configuration had been fixed using the training and validation subsets, the model was evaluated once on the case-independent test set. All subsequent development-stage analyses, including case-grouped five-fold cross-validation, ablation studies, sensitivity analysis, and baseline comparisons, were conducted exclusively on the development set using a fixed random seed (seed = 42) without modifying this configuration, whereas the optimizer comparison was repeated across five random seeds. The final training configuration is summarized in Table 3, and the complete workflow is presented in Algorithm 2.

**Algorithm 2.** Proposed ALGTO-Optimized ConvNeXt-Tiny–Vision Mamba Framework for Brain Stroke Classification**Input:** Brain CT image dataset *D* = {(*X_i_*, *y_i_*)} **Output:** Trained ConvNeXt-Tiny–Vision Mamba model and locked test-set performance **Begin**
❖**Dataset Preparation and Splitting**○Load the brain CT image dataset and remove duplicate images.○Divide the dataset at the case-group level into a development set (training and validation subsets) and a case-independent test set, which is withheld until final evaluation.❖**Image Preprocessing**○**For** each CT image *X_i_* ∈ *D*:
▪Resize the image, apply CLAHE and gamma correction, and normalize pixel intensities.○End **For**❖**Online Data Augmentation**○Apply random rotation, zooming, and contrast adjustment to the training images.❖**Dual-Stream Feature Extraction**○Extract hierarchical local features using ConvNeXt-Tiny.○Extract long-range contextual features using Vision Mamba.❖**Adaptive Hemispheric Difference Attention**○Align the local and contextual features and partition the aligned feature map at the central sagittal axis.○Reflect the right hemispheric representation and compute interhemispheric feature differences.○Generate adaptive attention weights and recalibrate the features to enhance stroke-sensitive responses.❖**Adaptive Cross-Attention Fusion**○Generate Query from the local features and Key–Value from the AHDA-enhanced features.○Compute cross-attention and generate the final fused feature representation.❖**ALGTO-Based Hyperparameter Optimization**○Obtain the optimal hyperparameter configuration using ALGTO (Algorithm 1) on the training and validation subsets.❖**Final Model Training**○Configure the framework using the ALGTO-optimized hyperparameters, the AdamW optimizer, a cosine annealing learning-rate schedule, and BCE loss.○Train the network for 50 epochs and retain the model state with the best validation performance.❖**Locked Test Evaluation**○Aggregate slice-level probabilities into case-level predictions by averaging and applying a threshold of 0.5.○Lock the final model configuration and evaluate it once on the case-independent test set to compute performance metrics.❖**Development-Stage Analyses**○Perform case-grouped five-fold cross-validation, ablation studies, sensitivity analysis, and baseline comparisons on the development set, without modifying the locked final configuration.**End**

### 3.8. Implementation Details

All experiments were performed on Google Colab Pro using an NVIDIA Tesla T4 GPU with 16 GB of GDDR6 memory. The implementation environment used Python 3.12.13, TensorFlow 2.19.0, NumPy 2.0.2, CUDA 12.5, and cuDNN 9.3.0.

### 3.9. Evaluation Metrics

The performance of the proposed framework was assessed using standard classification metrics, which assess overall classification performance, prediction reliability, sensitivity, and discriminative capability. Their mathematical formulations are presented in Equations (53)–(56).

(53) $$\mathrm{Accuracy}=\frac{\mathrm{TP}+\mathrm{TN}}{\mathrm{TP}+\mathrm{TN}+\mathrm{FP}+\mathrm{FN}}$$

(54) $$\mathrm{Precision}=\frac{\mathrm{TP}}{\mathrm{TP}+\mathrm{FP}}$$

(55) $$\mathrm{Recall}=\frac{\mathrm{TP}}{\mathrm{TP}+\mathrm{FN}}$$

(56) $$F1\text{-}\mathrm{score}=\frac{2\times \mathrm{Precision}\times \mathrm{Recall}}{\mathrm{Precision}+\mathrm{Recall}}$$

where TP, TN, FP, and FN represent true-positive, true-negative, false-positive, and false-negative predictions, respectively. As the model produces slice-level predictions, case-level predictions were obtained by averaging the Stroke probabilities of all slices within each case group and applying a threshold of 0.5. This averaging yields a single probability per case group irrespective of its slice count, so that each case group contributes equally to evaluation. All case-level metrics, confusion matrices, and statistical comparisons were computed from these predictions, and the ROC curve and ROC-AUC were constructed by varying the decision threshold over the case-level mean probabilities. Confidence intervals for accuracy were computed using the Wilson score method, and paired comparisons with baseline models were performed using McNemar’s exact test on the case-level predictions, with Holm–Bonferroni adjustment for multiple comparisons.

## 4. Results and Discussion

The final configuration was determined using the training and validation subsets, after which the final model was evaluated once on the case-independent test set (17 case groups) and locked, with no subsequent modification. All further analyses, including cross-validation, ablation studies, optimizer comparison, sensitivity analysis, and baseline comparisons, were performed exclusively on the development set (65 case groups) using case-grouped five-fold cross-validation and did not influence the final model or its test-set evaluation.

### 4.1. Final Model and Locked Test-Set Evaluation

#### 4.1.1. Training Performance Analysis

Figure 11 and Figure 12 show the slice-level training and validation accuracy and loss trajectories of the final model trained with the ALGTO-selected configuration. Both accuracies increased rapidly from approximately 58.8% and 56.5% at Epoch 1 to nearly 85% by Epoch 10, and stabilized from approximately Epoch 30 at 96.8–97.7% for training and 95.3–96.2% for validation. The corresponding losses decreased from 0.67 and 0.75 at Epoch 1 and converged to approximately 0.03 and 0.07, respectively, by Epoch 50. The small gap between the training and validation curves indicates stable convergence with limited overfitting.

#### 4.1.2. Classification Performance

Table 4 and Table 5 summarize the class-wise and overall case-level performance of the final model on the case-independent test set, evaluated once. All Stroke case groups were correctly identified (recall 100.00%), whereas one Normal case group was misclassified as Stroke, yielding a Stroke-class precision of 85.71%, an F1-score of 92.31%, and an accuracy of 94.12%. The macro-average and weighted-average F1-scores of 93.77% and 94.20% indicate balanced performance across both classes. The complete Stroke-class recall obtained under unweighted BCE loss further suggests that the moderate class imbalance did not introduce a detectable bias against the minority class, although the small test set limits the precision of these estimates.

#### 4.1.3. Error Analysis and Misclassifications

Figure 13 presents the case-level confusion matrix on the case-independent test set. Of the 17 test case groups, 16 were correctly classified: all 6 Stroke case groups were identified, and 10 of the 11 Normal case groups were correctly classified, with a single Normal case group predicted as Stroke. The absence of false negatives indicates high sensitivity to stroke case groups.

#### 4.1.4. Receiver Operating Characteristic Curve Analysis

Figure 14 shows the case-level ROC curve on the case-independent test set, with a ROC-AUC of 0.9697, indicating strong discrimination between Normal and Stroke case groups. Owing to the small test set (n = 17), the curve is step-shaped rather than smooth, and the AUC estimate should therefore be interpreted with caution.

#### 4.1.5. Prediction Visualization

Figure 15 presents representative predictions on the case-independent test set. Most Normal and Stroke images were correctly classified, consistent with the single misclassified case group observed in the confusion matrix.

#### 4.1.6. Visualization of Model Decisions

Grad-CAM was applied to highlight the image regions contributing to classification, computed with respect to the ACAF output (14 × 14 × 384), the final fused representation prior to GAP. As shown in Figure 16, in the representative Stroke example the strongest activation spatially coincides with a visible hypodense region, whereas the Normal example shows several discrete, lower-intensity activations distributed across the image without a single dominant focus.

Because the 14 × 14 feature map is upsampled to the original image size, individual activation regions cover comparatively large areas and may extend beyond anatomical boundaries. Moreover, as the model was not trained with any anatomical or foreground–background segmentation objective, activation in non-cerebral regions such as the skull or background does not necessarily indicate diagnostically meaningful attention and may reflect texture, intensity, or edge-related features. These examples are therefore presented as a qualitative illustration of model attention rather than a validated assessment of lesion localization, which would require comparison against expert-annotated lesion masks and blinded radiologist evaluation.

### 4.2. Development-Set Analyses

#### 4.2.1. Five-Fold Cross-Validation Analysis

Five-fold cross-validation was conducted on the development set (65 case groups, 1982 images), with the case-independent test set excluded. The development data were divided into five non-overlapping case-grouped folds of 13 case groups each, with four folds used for training and one for validation in each iteration. The hyperparameter configuration obtained through ALGTO was kept fixed across all folds rather than re-optimized within each fold. Table 6 summarizes the fold-wise results, and the corresponding training and validation curves are provided in the Supplementary Materials (Figure S1). Accuracy ranged from 84.62% to 92.31%, with a mean of 90.77 ± 3.44%, and the mean precision, recall, and F1-score were 89.33 ± 9.83%, 88.00 ± 10.95%, and 87.92 ± 4.54%, respectively. The wider variability in precision and recall reflects the influence of individual case-group outcomes within the small 13-case-group folds, indicating that these estimates should be interpreted with caution.

#### 4.2.2. Ablation Study

Two ablation analyses were conducted on the development set using case-grouped five-fold cross-validation: a component-based ablation to assess the contribution of each architectural module, and an optimization-based ablation to assess the contribution of ALGTO.

Table 7 summarizes the component-wise ablation, in which all configurations were trained under identical, non-optimized hyperparameters. Adding Vision Mamba to the ConvNeXt-Tiny baseline produced the largest improvement, increasing accuracy from 76.92% to 87.69%, recall from 68.00% to 84.00%, and ROC-AUC from 0.800 to 0.900. The incorporation of AHDA further increased recall to 88.00%, F1-score to 86.40%, and ROC-AUC to 0.925. The addition of ACAF did not change accuracy (89.23%) but increased precision and ROC-AUC (0.950) and reduced the fold-wise variability of the F1-score (SD from 4.56% to 3.04%), indicating more consistent performance across folds. Across the 65 development case groups, the number of misclassifications decreased from 15 for ConvNeXt-Tiny to 8 and 7 with the addition of Vision Mamba and AHDA, respectively, and remained at 7 after the addition of ACAF; the corresponding fold-wise confusion matrices for each configuration are provided in the Supplementary Materials (Figure S2).

Table 8 summarizes the optimization-based ablation, in which the complete architecture was trained with default hyperparameters and with configurations selected by conventional GTO, GTO with adaptive Lévy flight, and the proposed ALGTO. Conventional GTO and GTO with adaptive Lévy flight yielded the same mean accuracy as the default configuration (89.23%) but with substantially greater fold-wise variability (SD of 8.77% compared with 4.21%), together with lower ROC-AUC (0.925 and 0.930, respectively) and, for conventional GTO, lower recall (84.00%). The proposed ALGTO achieved the highest mean accuracy (90.77%), precision (89.33%), and F1-score (87.92%), reducing the number of misclassified development case groups from 7 to 6. Its mean ROC-AUC (0.950) matched that of the default configuration, while both its accuracy and ROC-AUC exhibited the lowest fold-wise variability (SD of 3.44% and 0.035, respectively). These results indicate that the improvement attributable to ALGTO is modest in magnitude but is accompanied by more consistent performance across folds. The corresponding fold-wise confusion matrices for each optimization strategy are provided in the Supplementary Materials (Figure S3).

#### 4.2.3. Comparative Evaluation of Optimization Strategies

ALGTO was compared with Random Search, Grid Search, Bayesian Optimization, conventional GTO, and GTO with adaptive Lévy flight using the same search space and a matched budget of 96 model evaluations, with each candidate trained for 50 epochs and scored by validation loss. Random Search sampled 96 configurations independently at random within the search space defined in Table 1. Grid Search evaluated a full factorial grid of 96 configurations, comprising four logarithmically spaced learning rates (1 × 10^−5^, 4.64 × 10^−5^, 2.15 × 10^−4^, and 1 × 10^−3^), the three batch sizes, two weight-decay values (1 × 10^−5^ and 1 × 10^−3^), and four linearly spaced dropout rates (0.10, 0.23, 0.37, and 0.50). Bayesian Optimization employed a Gaussian-process surrogate over the normalized search space, with the learning rate and weight decay on a logarithmic scale, and an expected-improvement acquisition function (ξ = 0.01); it was initialized with 10 randomly sampled configurations, followed by 86 sequential evaluations. Conventional GTO, GTO with adaptive Lévy flight, and ALGTO used a population of 8 over 12 iterations (Table 2). For Grid Search, the evaluated configurations were identical across seeds, so the random seed affected only weight initialization and data ordering, whereas for the remaining strategies it additionally controlled candidate sampling or population initialization.

Each strategy was repeated with five random seeds; the mean, SD, and 95% CI of the best validation loss are reported in Table 9, and per-seed values are provided in the Supplementary Materials (Table S2). ALGTO obtained the lowest mean best validation loss (0.1416 ± 0.0025; 95% CI: 0.1385–0.1447), followed by GTO with adaptive Lévy flight (0.1562 ± 0.0029) and Bayesian Optimization (0.1618 ± 0.0039), whereas Random Search yielded the highest (0.1844 ± 0.0044). As all strategies used the same evaluation budget and training duration, mean optimization times were comparable (1520–1575 min per run); the five ALGTO runs required approximately 128 h of cumulative GPU computation.

#### 4.2.4. Convergence Analysis of ALGTO

Figure 17 presents the convergence trajectory of ALGTO over the 96 model evaluations (population size of 8 over 12 iterations). The best validation loss decreased rapidly during early evaluations, followed by progressively smaller improvements, indicating a transition from broad exploration toward refinement of promising configurations.

#### 4.2.5. Hyperparameter Sensitivity Analysis

The sensitivity of the framework to the ALGTO-selected hyperparameters was assessed on the development set using case-grouped five-fold cross-validation, by varying each hyperparameter individually while holding the others at their selected values. As summarized in Table 10, the ALGTO-selected configuration (learning rate of 0.00005, batch size of 16, weight decay of 0.0001, and dropout rate of 0.30) yielded the highest mean accuracy (90.77%) and F1-score (87.92%) across all four hyperparameters. Deviations from these values reduced mean accuracy to between 76.92% and 87.69%, with the largest decreases observed for higher learning rates (76.92% at 0.001) and higher weight decay values (80.00% at 0.0005 and 0.001). The reductions were driven primarily by lower recall, which decreased to between 64.00% and 80.00% in most alternative settings, indicating that suboptimal configurations predominantly increased the number of missed Stroke case groups. These results indicate a marked dependence of classification performance on the selected hyperparameters, supporting the use of systematic hyperparameter optimization.

#### 4.2.6. Performance Comparison with Baseline Models

The proposed framework was compared with representative CNN-based models (VGG16, ResNet50, DenseNet121, and EfficientNet-B0) on the development set using the same case-grouped five-fold cross-validation, preprocessing, and case-level aggregation. As summarized in Table 11, the proposed framework achieved the highest mean accuracy (90.77%), precision (89.33%), F1-score (87.92%), and ROC-AUC (0.950), followed by EfficientNet-B0 (86.15%), DenseNet121 (83.08%), ResNet50 (76.92%), and VGG16 (67.69%). EfficientNet-B0 attained the same mean recall as the proposed framework (88.00%), but with markedly lower precision (78.95%), indicating a higher number of Normal case groups misclassified as Stroke. Across the 65 development case groups, the number of misclassifications was 21, 15, 11, and 9 for VGG16, ResNet50, DenseNet121, and EfficientNet-B0, respectively, compared with 6 for the proposed framework. The 95% Wilson score confidence intervals for pooled out-of-fold accuracy overlapped between the proposed framework (81.29–95.70%) and EfficientNet-B0, DenseNet121, and ResNet50, but not VGG16 (55.61–77.80%).

McNemar’s exact test applied to the paired out-of-fold case-level predictions of the 65 development case groups (Table 12) indicated that the proposed framework misclassified significantly fewer case groups than VGG16 (p = 0.0015) and ResNet50 (p = 0.0352), whereas the differences relative to DenseNet121 (p = 0.2668) and EfficientNet-B0 (p = 0.5488) were not statistically significant. After Holm–Bonferroni adjustment for the four comparisons, only the difference relative to VGG16 remained significant. Accordingly, the advantage of the proposed framework over the stronger baselines should be interpreted descriptively.

### 4.3. Performance Comparison with Existing Methods

Table 13 lists the accuracies reported for representative published stroke-classification methods, ranging from 87.51% to 93.36%, alongside the 94.12% obtained by the proposed framework on the locked case-level test set. These values were taken from the respective studies rather than obtained by re-implementation, under different datasets, data partitions, preprocessing procedures, evaluation settings, evaluation units (slice- or case-level), and input formats; they are therefore presented as contextual reference rather than as a direct head-to-head comparison.

From a bioinspired design perspective, the framework applies biological principles at two levels: the bilateral organization of the cerebral hemispheres informs feature representation through AHDA, whereas the collective behavior of gorilla troops provides the population-search foundation of ALGTO.

## 5. Conclusions

Experimental evaluation on a locked, single-use test set of 17 case groups indicates that the framework can classify rendered axial CT slice images as Normal or Stroke on the present dataset, achieving 94.12% accuracy, a weighted F1-score of 94.20%, and a ROC-AUC of 0.9697. The framework combines local and long-range representation learning with hemispheric-difference attention and ALGTO-based hyperparameter optimization. On the development set, case-grouped five-fold cross-validation yielded a mean accuracy of 90.77 ± 3.44%, ablation analyses showed incremental gains consistent with the intended contribution of each module, and the proposed framework misclassified significantly fewer case groups than VGG16 after Holm–Bonferroni adjustment, whereas differences relative to the remaining baselines were not statistically significant after adjustment. The sensitivity analysis showed a strong dependence of performance on the selected hyperparameters.

Several limitations warrant acknowledgment. The study relies on a single public dataset with small test (17 case groups) and development (65 case groups) cohorts and lacks external validation. Partitioning relied on filename-defined case groups that could not be cross-referenced with clinical records, so patient-level independence between subsets cannot be guaranteed. The inputs are rendered slice images without Hounsfield units, windowing metadata, acquisition parameters, or slice ordering, which limits clinical interpretation and precludes conclusions regarding diagnostic performance on clinical CT examinations. Training employed slice-level loss, so case groups with more slices contributed moderately more to optimization, whereas evaluation weighted each case group equally. The binary Stroke label does not distinguish ischemic from hemorrhagic subtypes. The main pipeline used a single random seed, and the ALGTO-selected hyperparameters were held fixed across folds rather than re-optimized within each fold, which may render the cross-validation estimates slightly optimistic. Finally, AHDA assumes approximately midline-centered framing, which could not be verified, and uses a fixed image midpoint without registration or midline detection, so its left–right comparison may be less precise for translated, rotated, or off-center scans; it represents a computational abstraction of bilateral cerebral anatomy rather than a physiological model.

Future research will address these limitations through external validation on datasets with verifiable patient-level structure, nested evaluation with multiple random seeds, case-balanced training strategies, automated midline detection or registration prior to hemispheric comparison, multiclass stroke-subtype classification, complementary neuroimaging modalities, and prospective clinical validation.

## Figures and Tables

**Figure 1:**
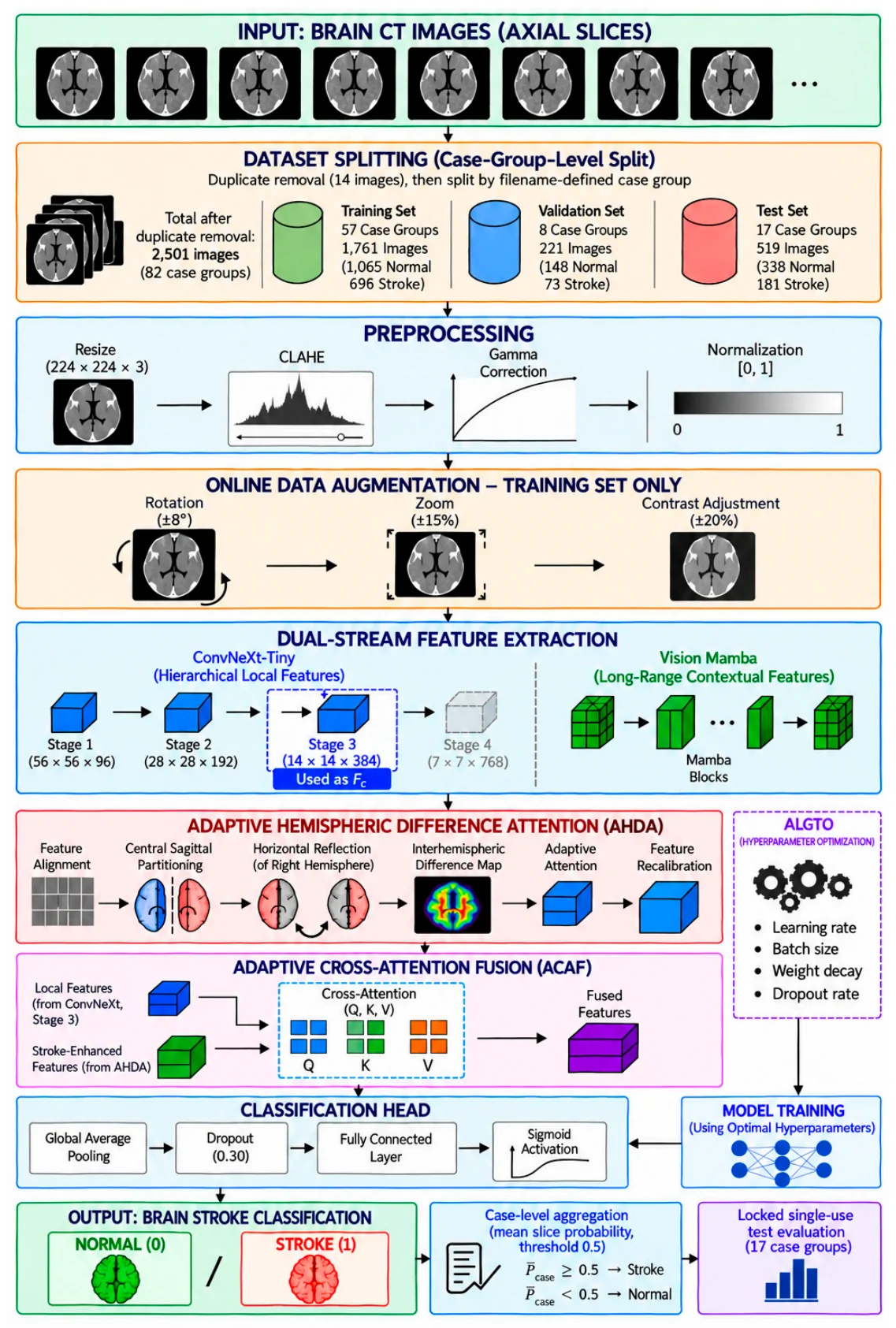
Workflow of the proposed brain stroke detection model.

**Figure 2:**
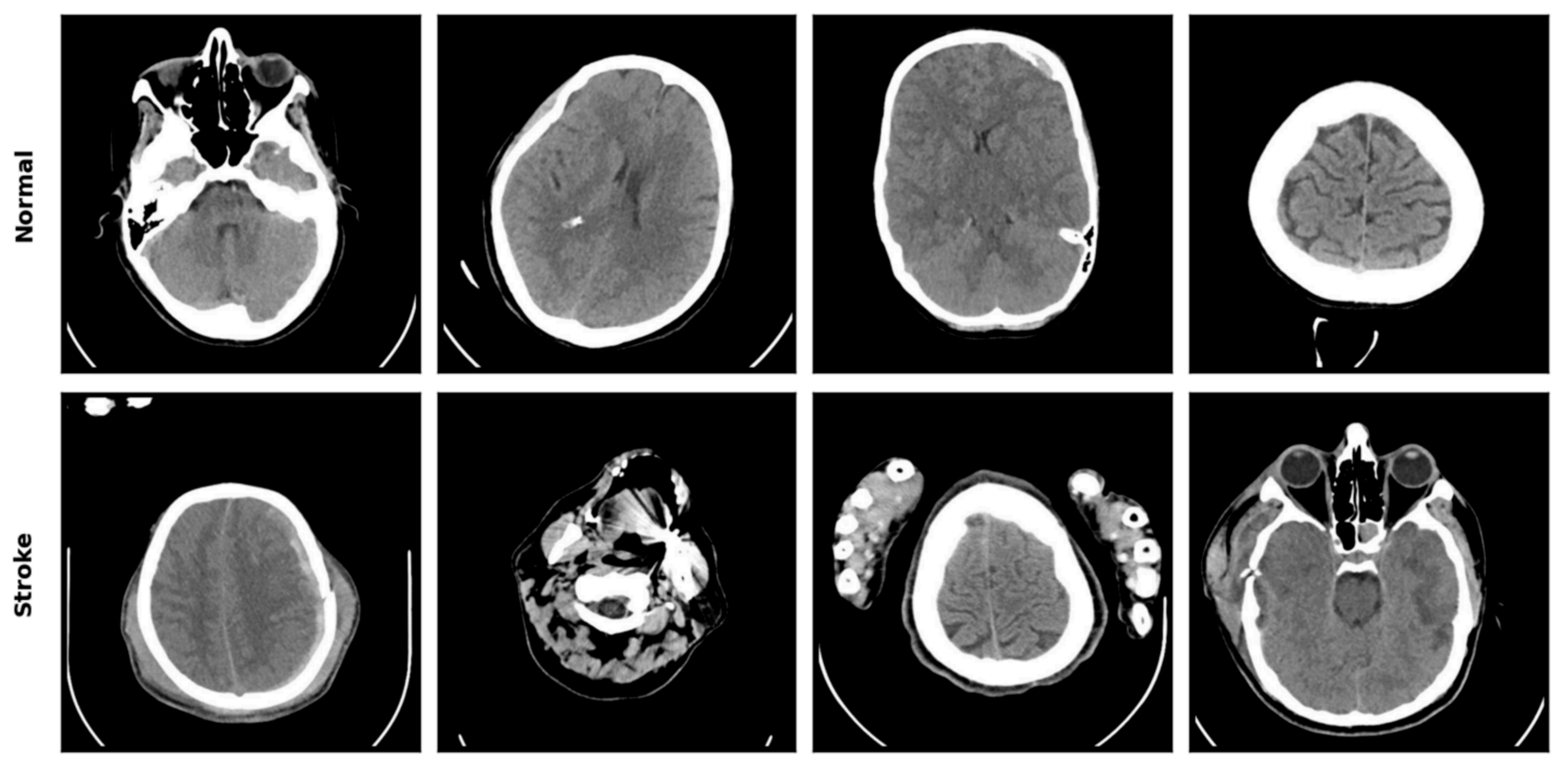
Representative CT images of the normal and stroke classes.

**Figure 3:**
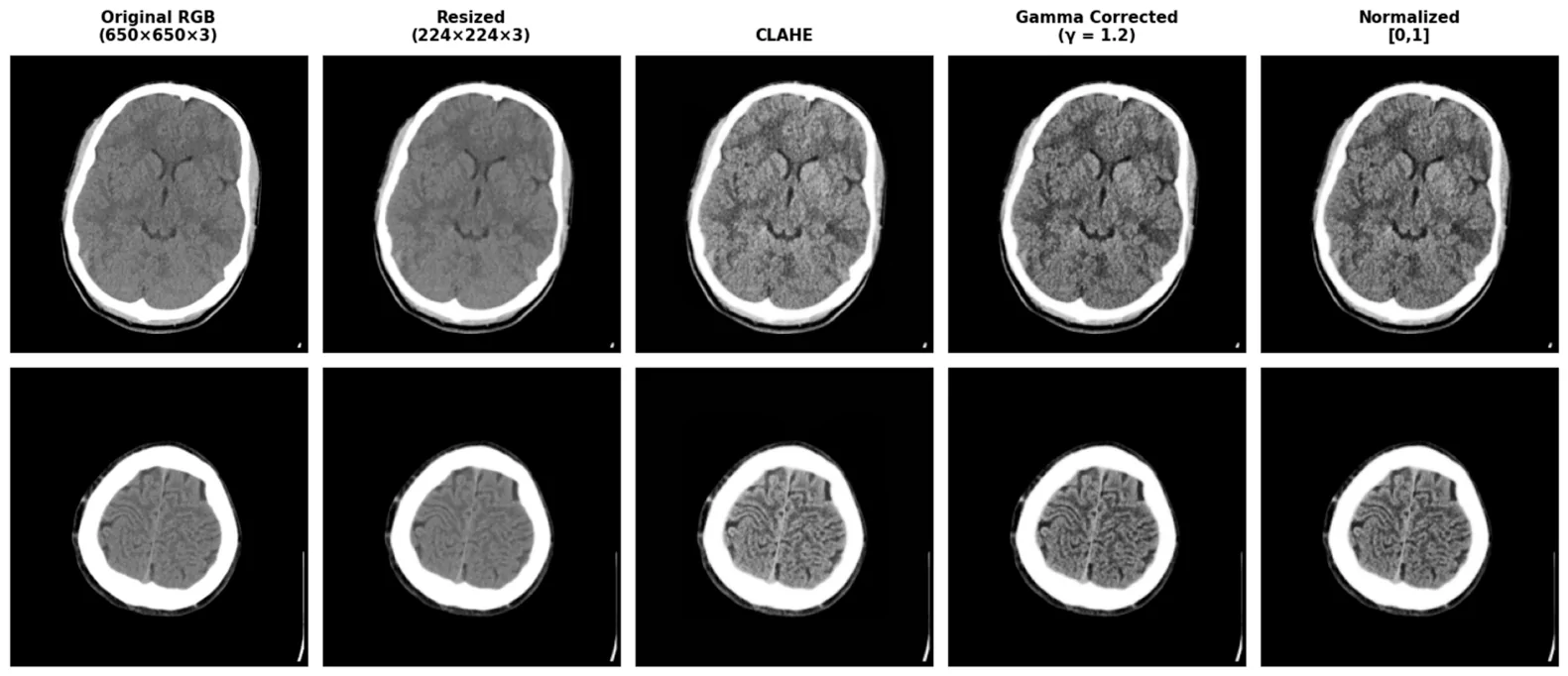
Image preprocessing pipeline.

**Figure 4:**
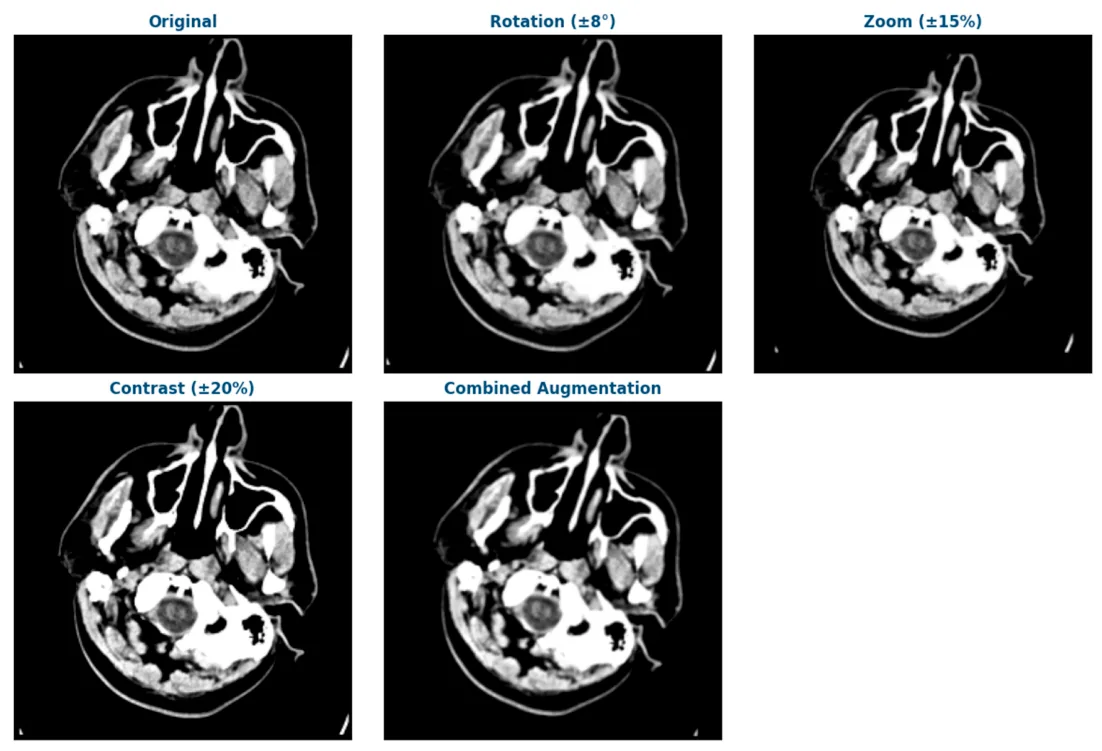
Representative online augmentation techniques.

**Figure 5:**
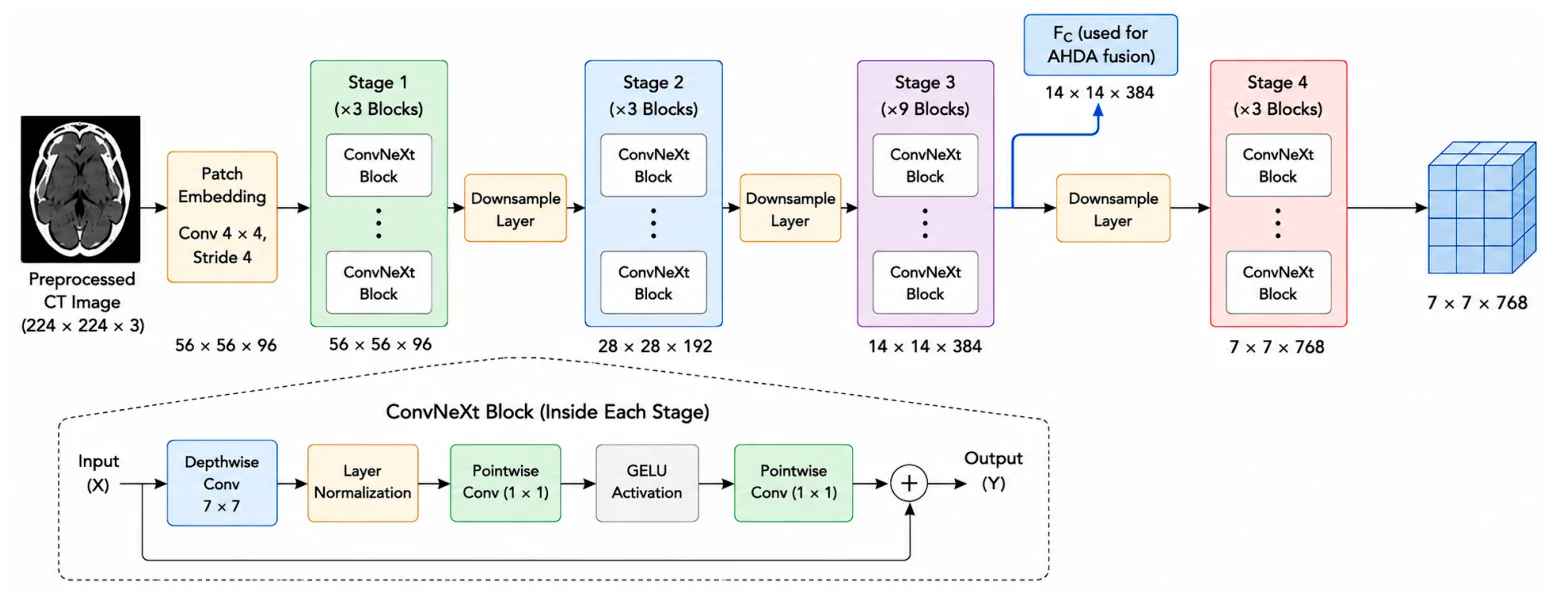
Hierarchical local feature extraction using ConvNeXt-Tiny.

**Figure 6:**
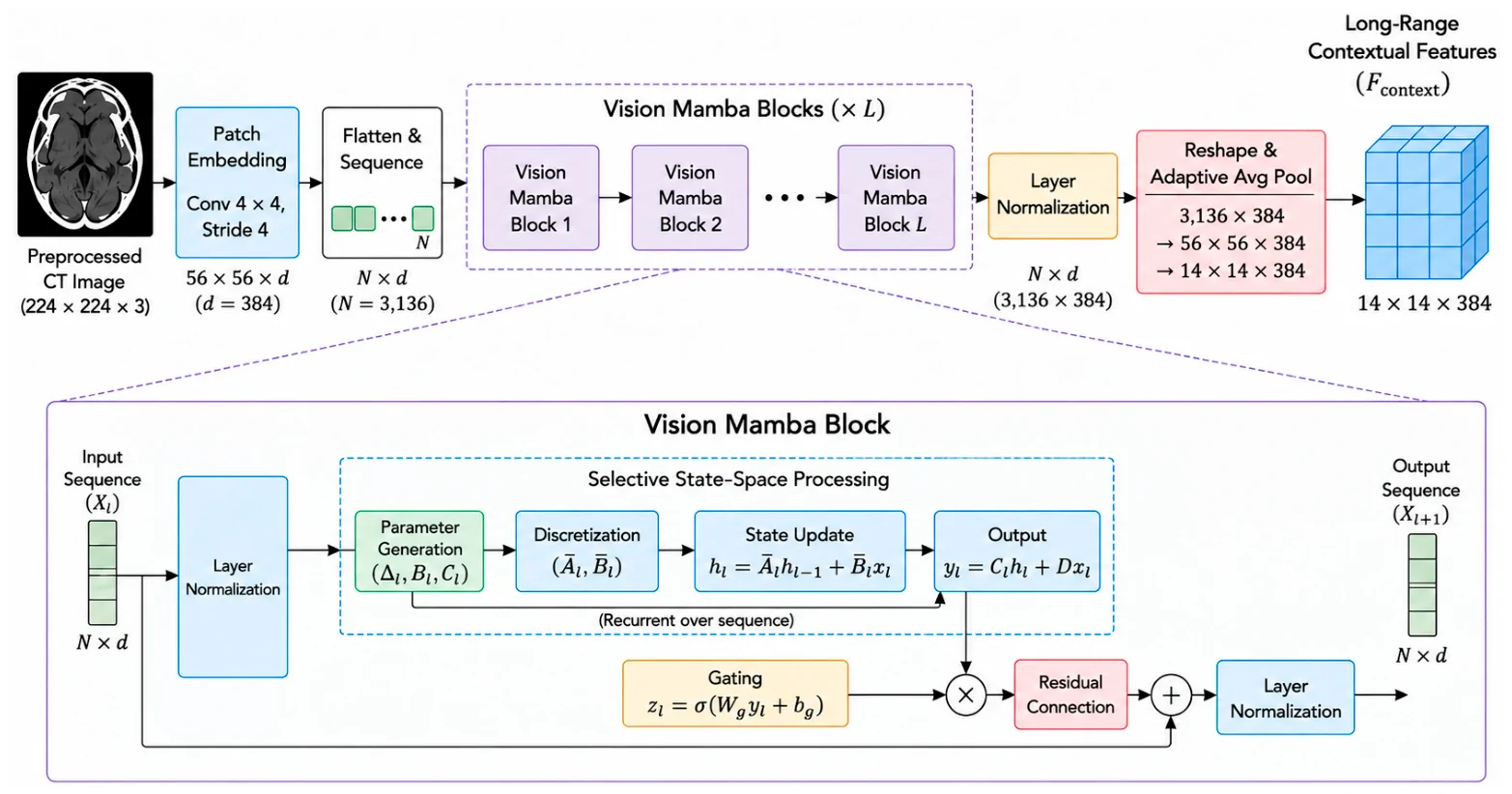
Selective state-space processing and hierarchical feature learning in the Vision Mamba branch.

**Figure 7:**
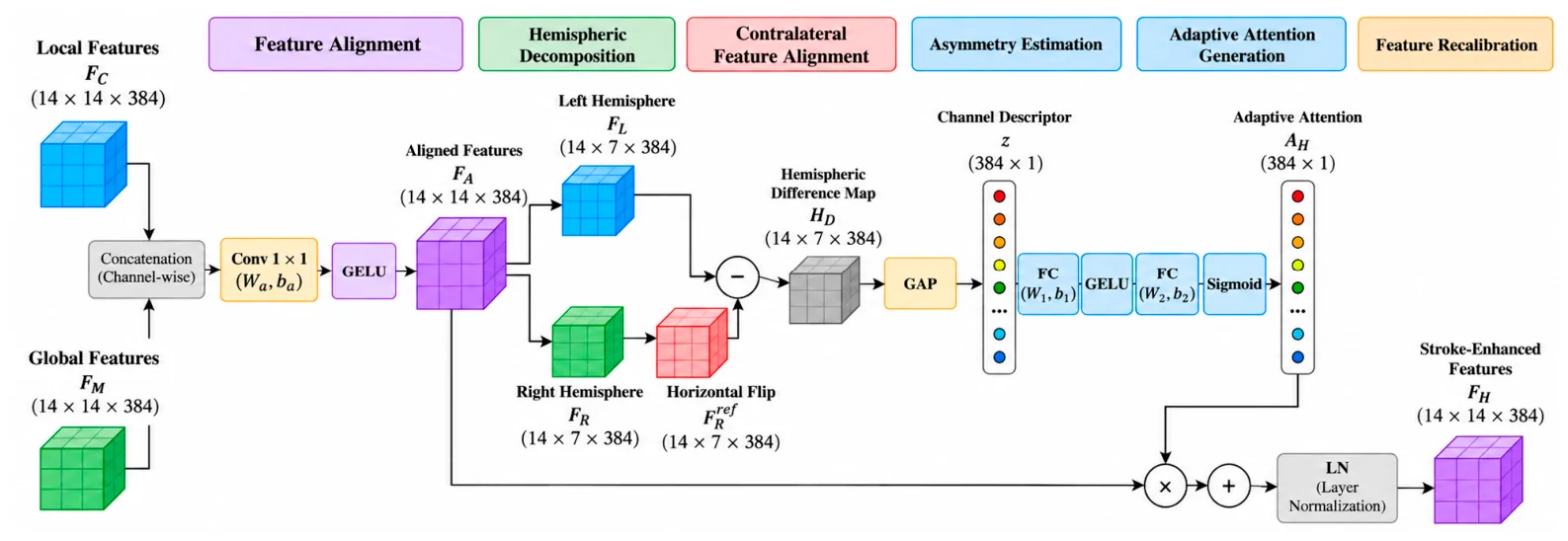
Adaptive Hemispheric Difference Attention module.

**Figure 8:**
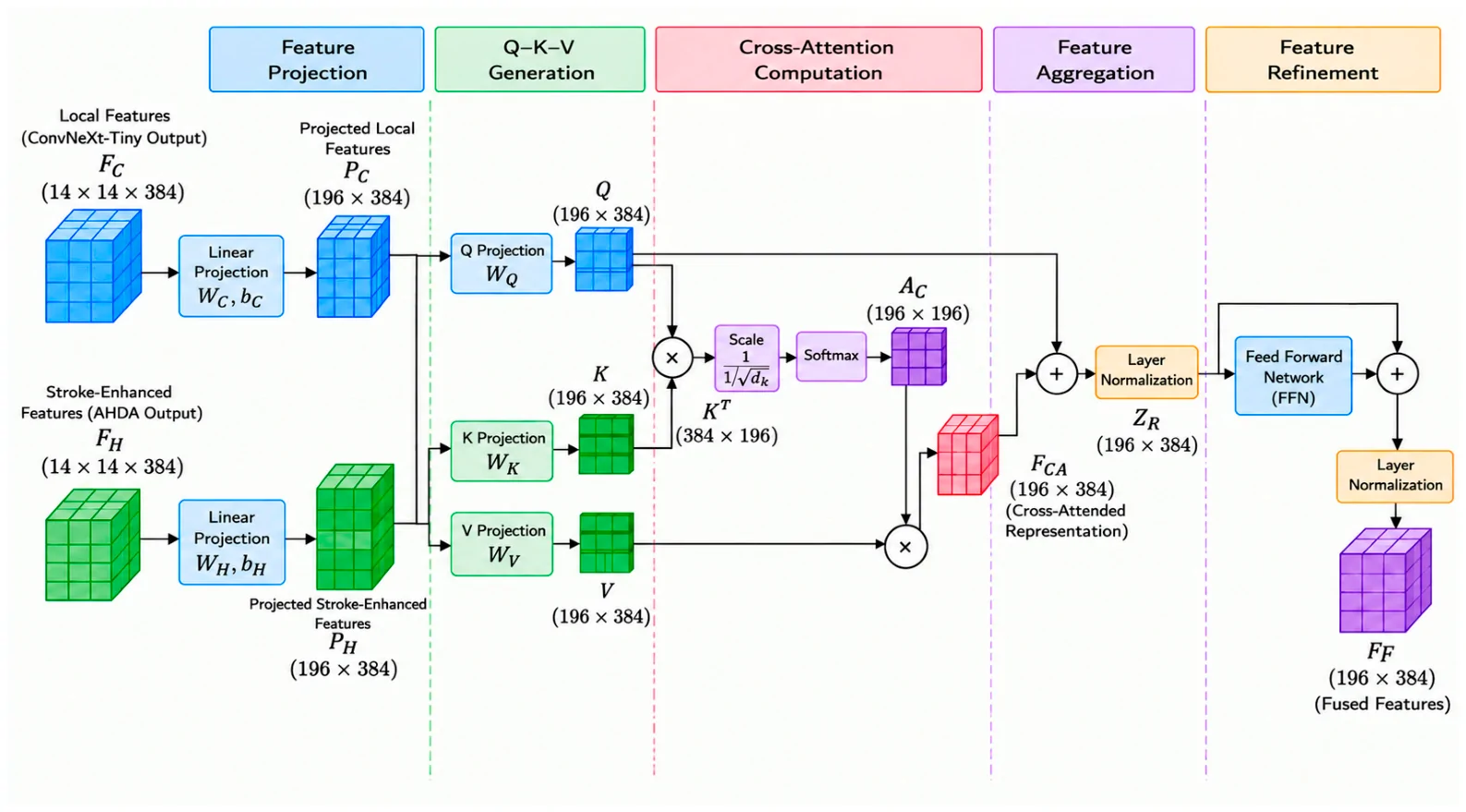
Cross-attention-based integration of local and stroke-enhanced feature representations.

**Figure 9:**
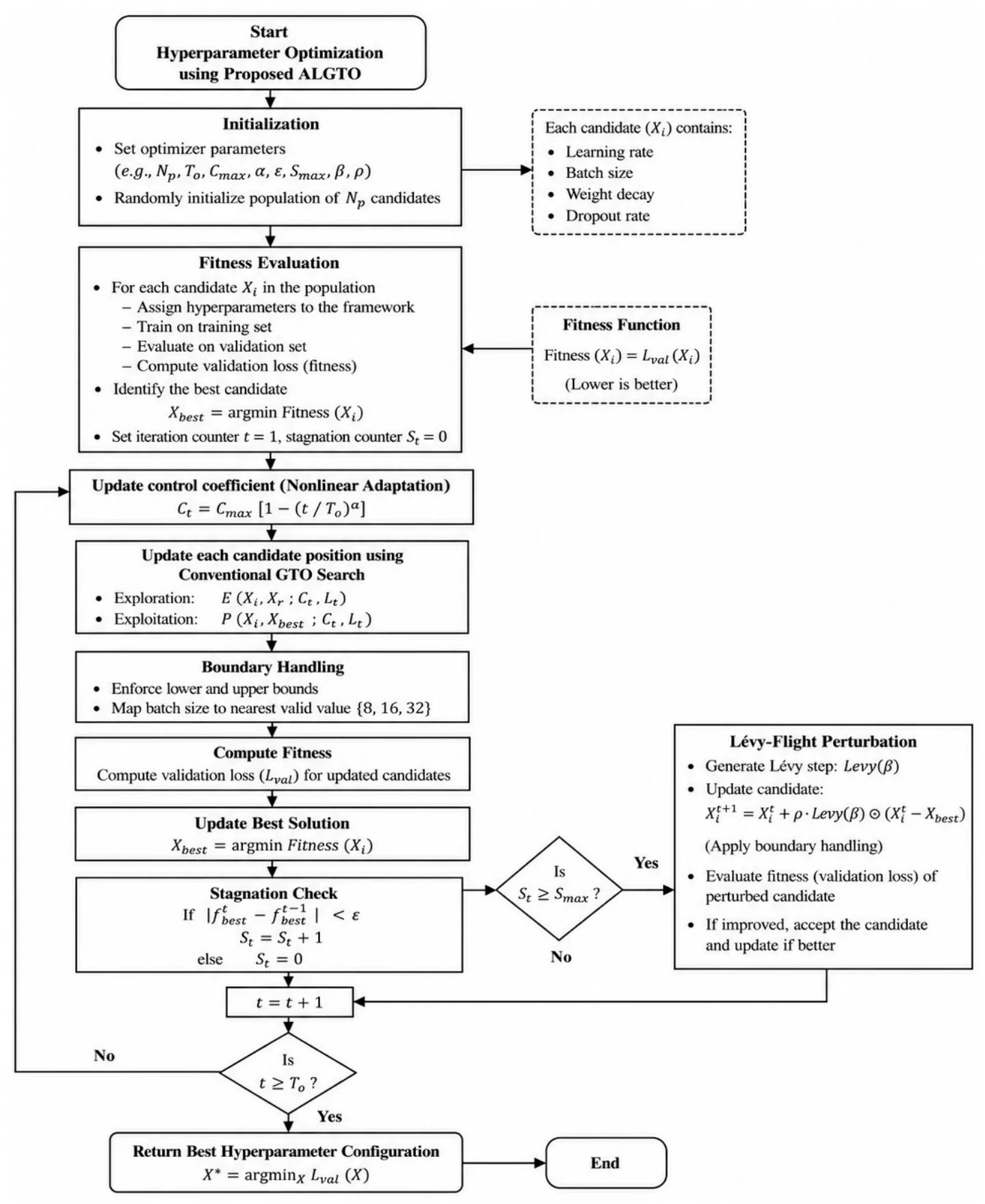
Flowchart of the proposed hybrid ALGTO hyperparameter optimization framework.

**Figure 10:**
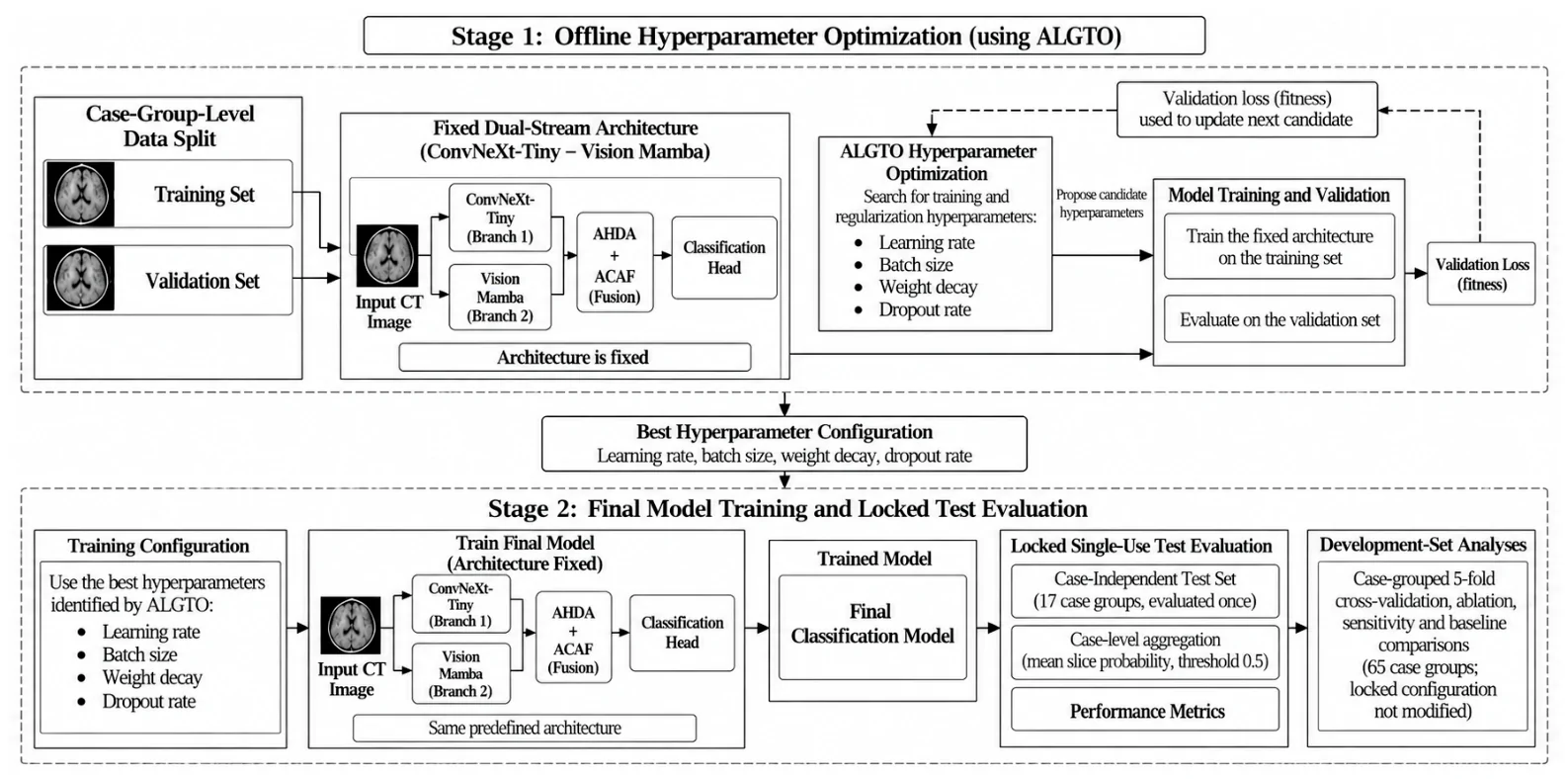
End-to-end ALGTO optimization and final training pipeline.

**Figure 11:**
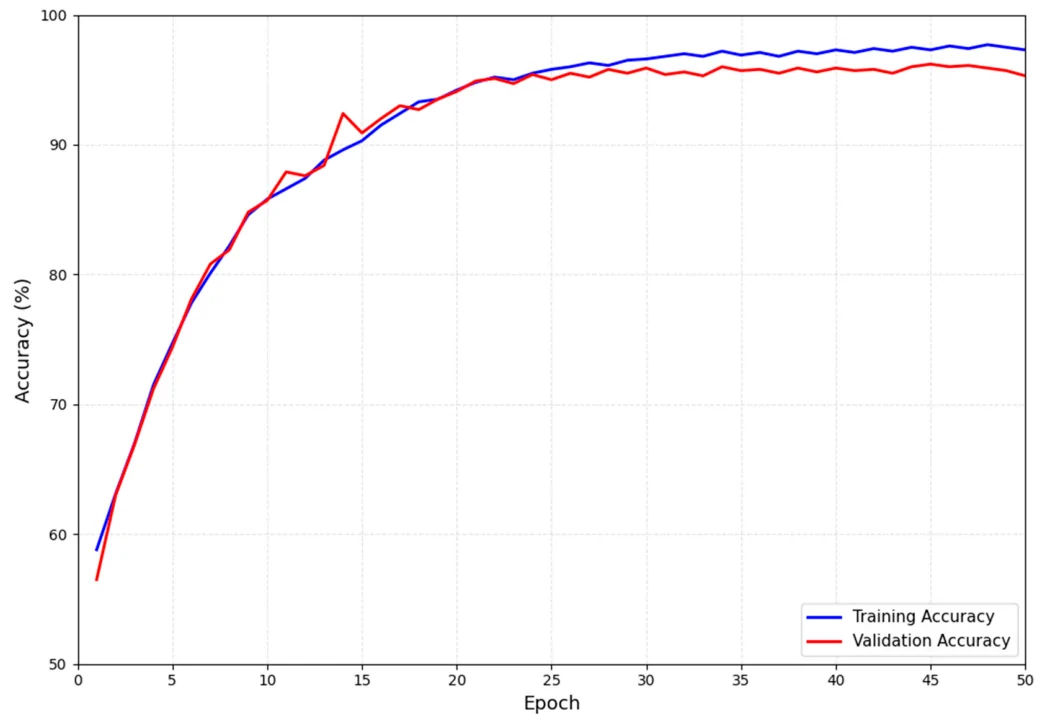
Training and validation accuracy trends.

**Figure 12:**
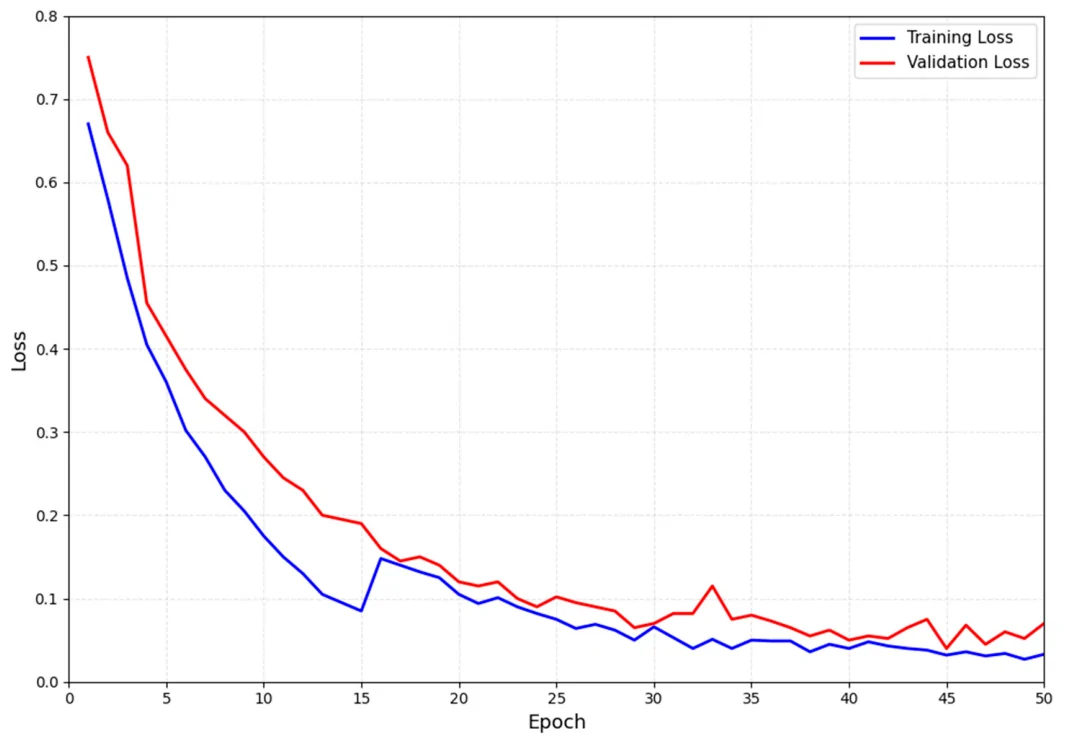
Training and validation loss trends.

**Figure 13:**
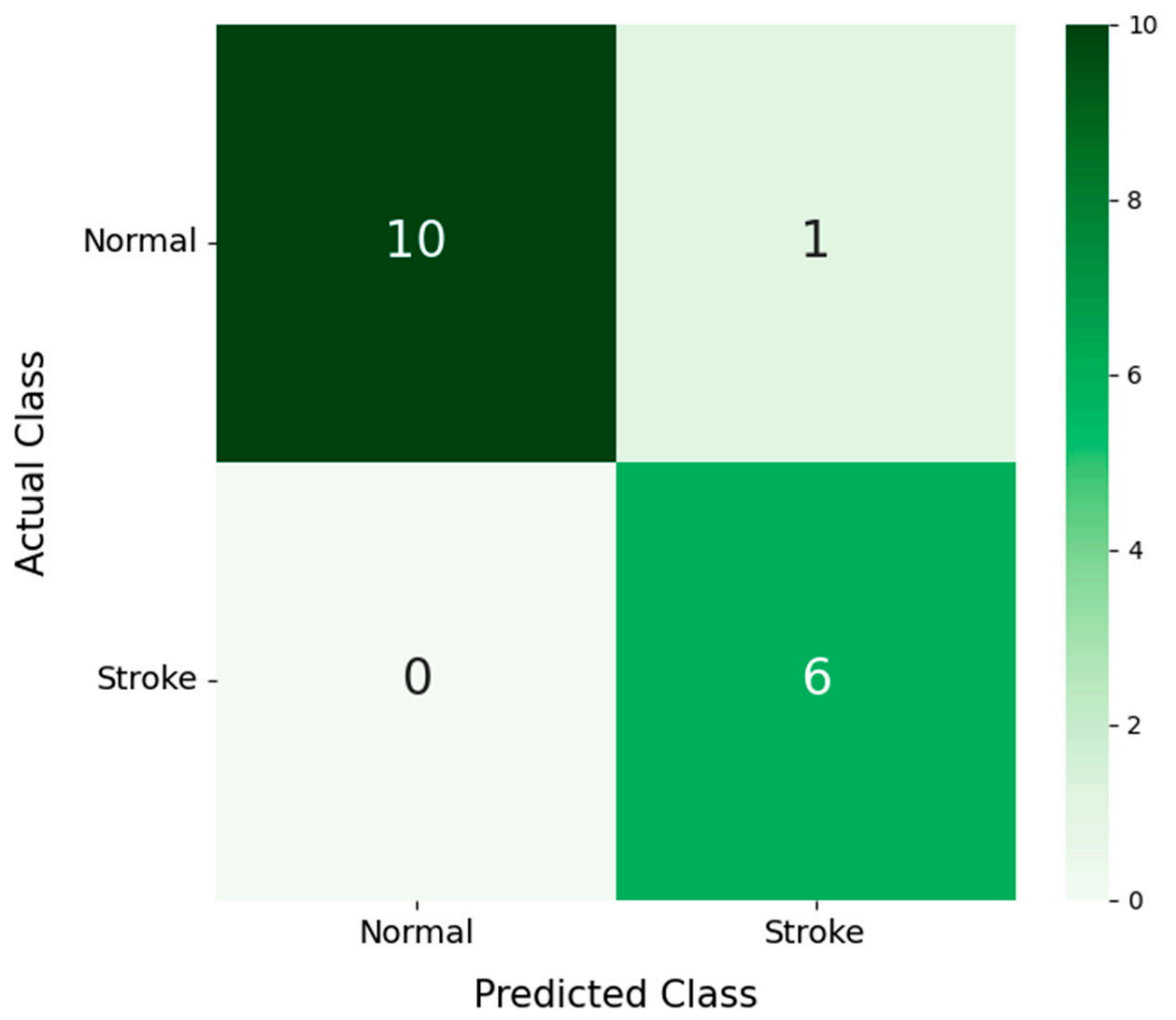
Case-level confusion matrix on the case-independent test set.

**Figure 14:**
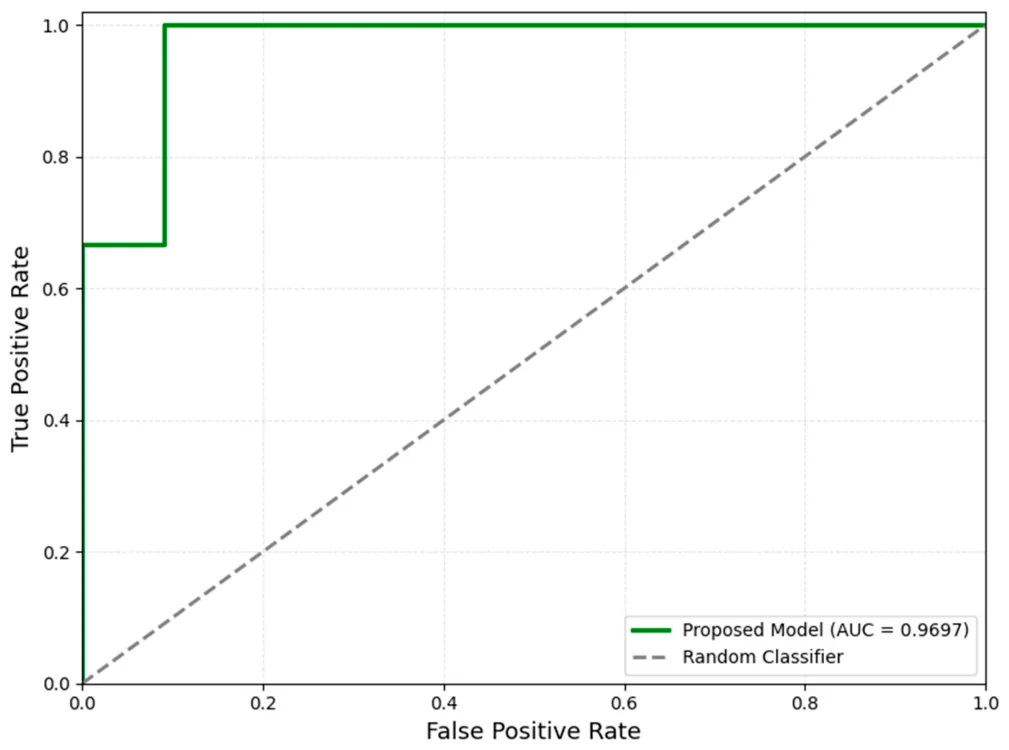
Case-level ROC curve on the case-independent test set.

**Figure 15:**
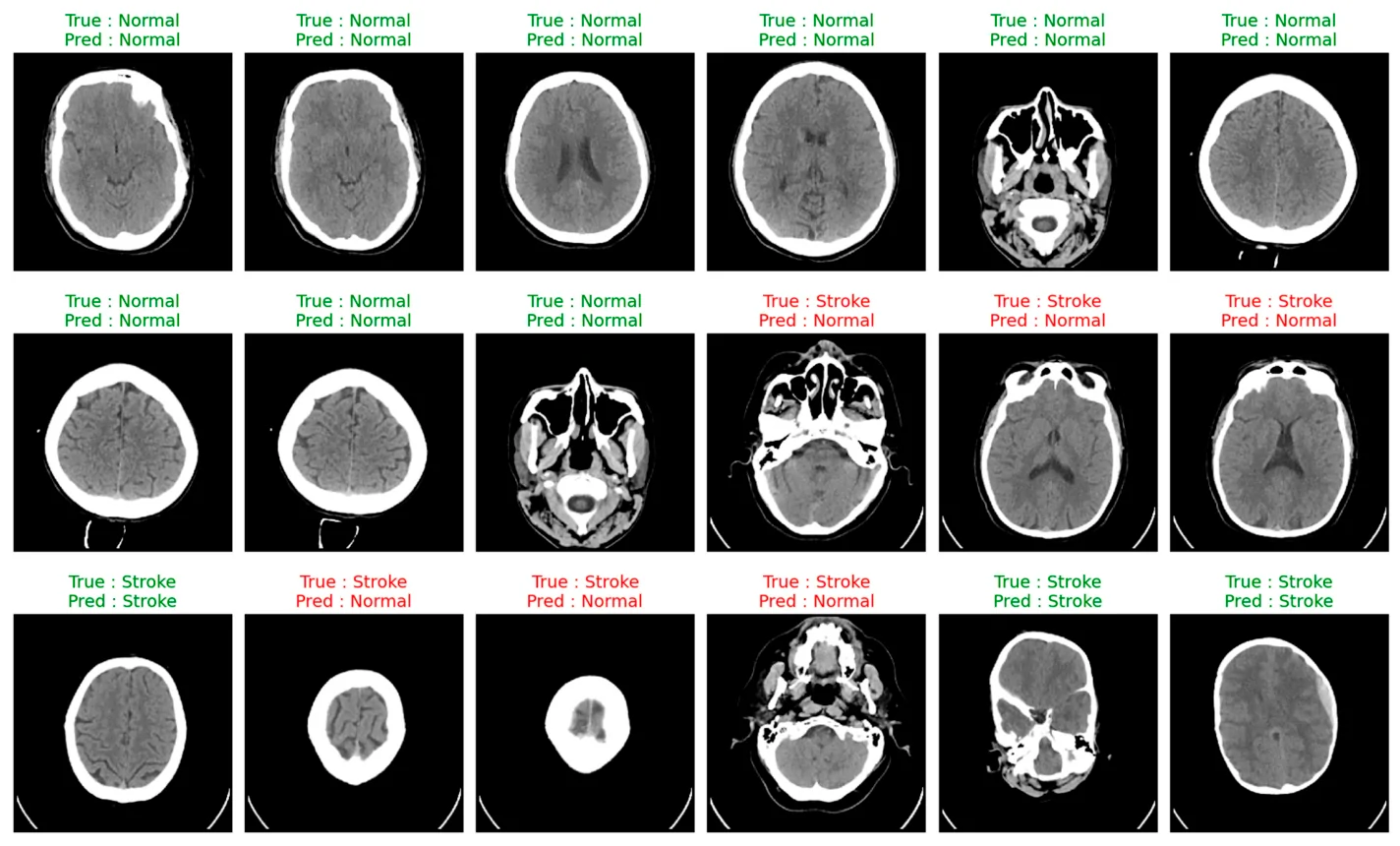
Representative prediction results on the case-independent test set.

**Figure 16:**
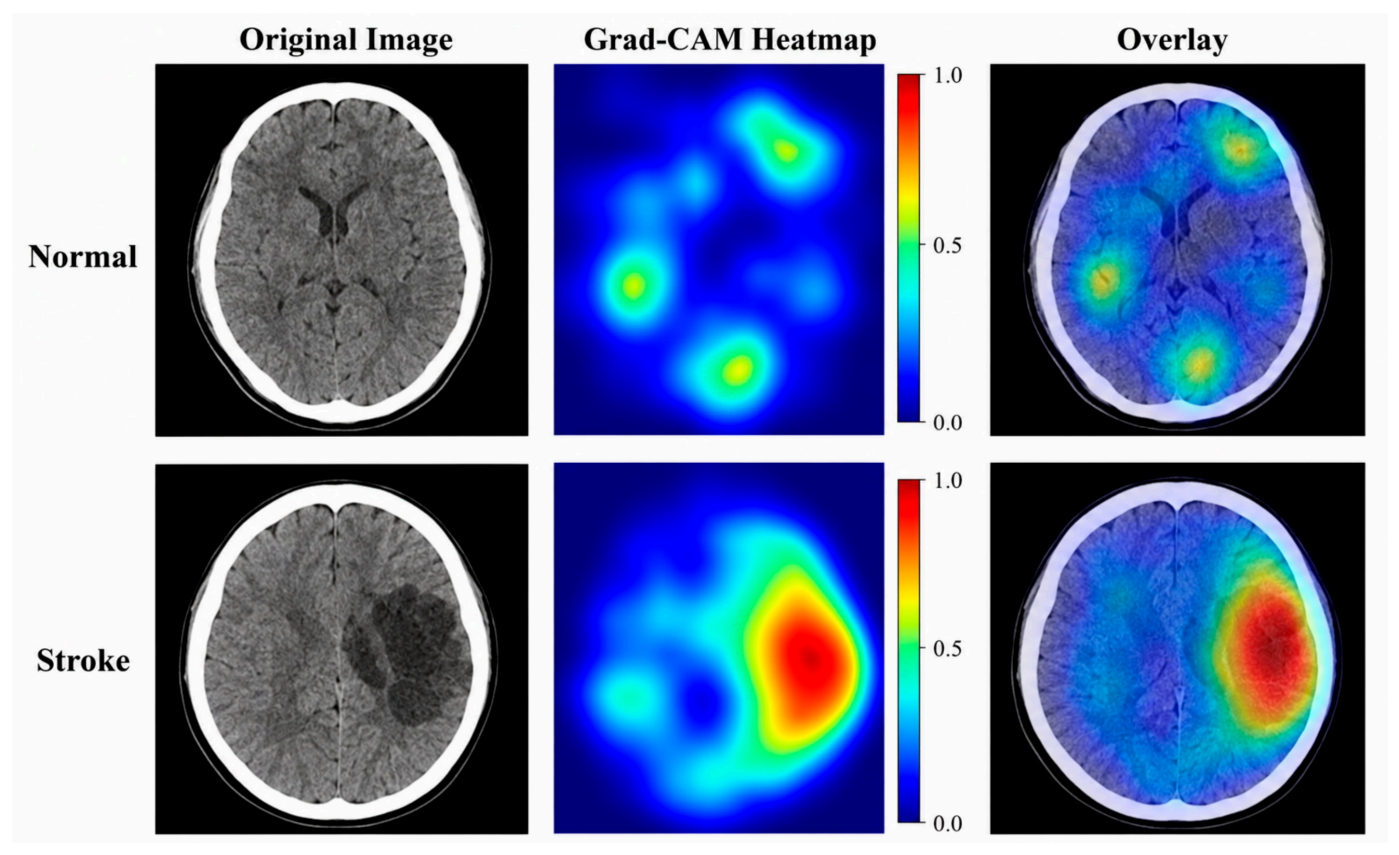
Grad-CAM visualization.

**Figure 17:**
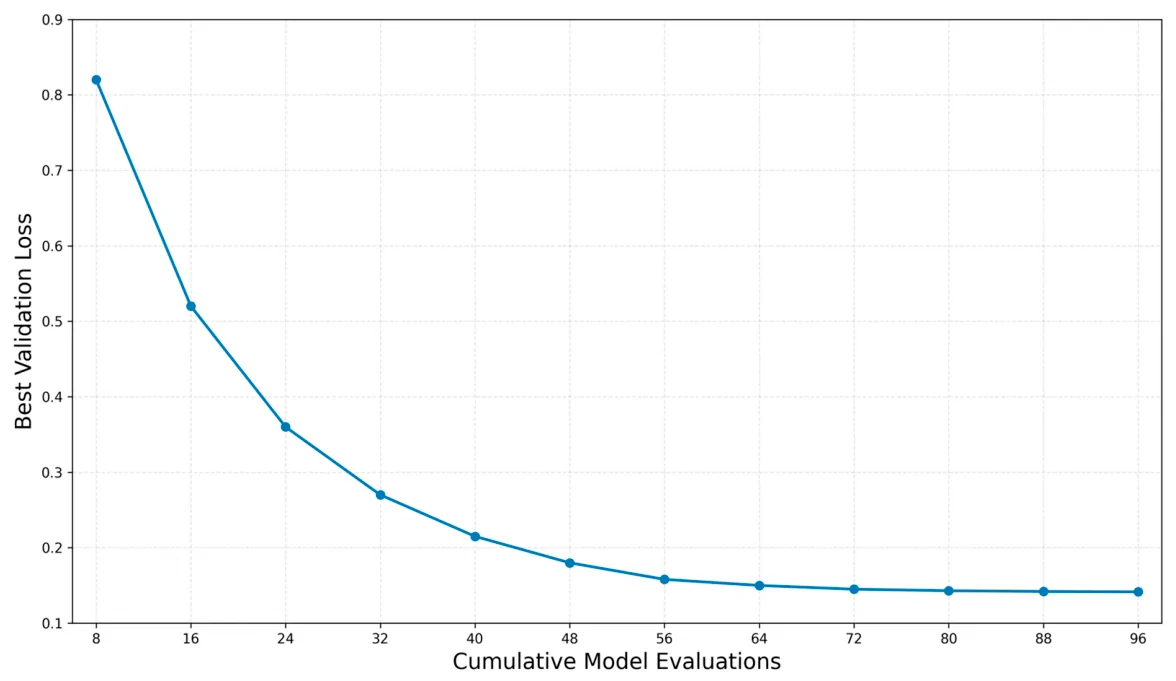
Convergence behavior of the proposed ALGTO on best validation loss over cumulative model evaluations.

**Table 1:** Search space of the optimized hyperparameters.

Hyperparameter	Search Range
Learning rate (η)	0.00001–0.001
Batch size (B)	{8, 16, 32}
Weight decay (λ)	0.00001–0.001
Dropout rate (*p_d_*)	0.10–0.50

**Table 2:** ALGTO hyperparameter configuration and optimization budget.

Parameter	Symbol	Value
Population size	*N_p_*	8
Maximum iterations	*T_o_*	12
Maximum control coefficient	*C*_max_	1.0
Nonlinear adaptation exponent	*α*	2.0
Stagnation tolerance	*ε*	10^−4^
Stagnation threshold	*S*_max_	5
Lévy exponent	*β*	1.5
Lévy scaling factor	*ρ*	0.01
Total model evaluations	*N_p_* × *T_o_*	96

**Table 3:** Final training configuration of the proposed framework.

Parameter	Final V50.	Selection
Optimizer	AdamW	Fixed
Number of epochs	50	
Learning-rate scheduler	Cosine annealing	
Learning rate	0.00005	ALGTO-optimized
Batch size	16	
Weight decay	0.0001	
Dropout rate	0.30	

**Table 4:** Class-wise classification performance on the case-independent test set.

Class/Metric	Precision	Recall	F1-Score	Support
Normal	1.0000	0.9091	0.9524	11
Stroke	0.8571	1.0000	0.9231	6
Accuracy	—	—	0.9412	17
Macro Average	0.9286	0.9545	0.9377	17
Weighted Average	0.9496	0.9412	0.9420	17

**Table 5:** Overall classification performance on the case-independent test set.

Performance Metric	Value (%)
Accuracy	94.12
Precision	85.71
Recall	100.00
F1-score	92.31

**Table 6:** Performance of five-fold cross-validation with confusion matrix components.

Fold	TP	TN	FP	FN	Accuracy (%)	Precision (%)	Recall (%)	F1-Score (%)
Fold 1	5	7	1	0	92.31	83.33	100.00	90.91
Fold 2	4	8	0	1	92.31	100.00	80.00	88.89
Fold 3	4	7	1	1	84.62	80.00	80.00	80.00
Fold 4	5	7	1	0	92.31	83.33	100.00	90.91
Fold 5	4	8	0	1	92.31	100.00	80.00	88.89
Mean ± SD	4.40 ± 0.55	7.40 ± 0.55	0.60 ± 0.55	0.60 ± 0.55	90.77 ± 3.44	89.33 ± 9.83	88.00 ± 10.95	87.92 ± 4.54

**Table 7:** Component-wise ablation on the development set (case-grouped five-fold cross-validation, mean ± SD).

Configuration	Accuracy (%)	Precision (%)	Recall (%)	F1-Score (%)	ROC-AUC
ConvNeXt-Tiny	76.92 ± 5.44	71.33 ± 7.94	68.00 ± 10.95	69.21 ± 7.53	0.800 ± 0.079
ConvNeXt-Tiny + Vision Mamba	87.69 ± 4.21	84.67 ± 8.69	84.00 ± 8.94	83.96 ± 5.47	0.900 ± 0.050
ConvNeXt-Tiny + Vision Mamba + AHDA	89.23 ± 4.21	86.95 ± 12.68	88.00 ± 10.95	86.40 ± 4.56	0.925 ± 0.050
ConvNeXt-Tiny + Vision Mamba + AHDA + ACAF	89.23 ± 4.21	88.57 ± 15.65	88.00 ± 10.95	86.67 ± 3.04	0.950 ± 0.050

**Table 8:** Optimization-based ablation of the ConvNeXt-Tiny + Vision Mamba + AHDA + ACAF architecture on the development set (case-grouped five-fold cross-validation, mean ± SD).

Optimization Strategy	Accuracy (%)	Precision (%)	Recall (%)	F1-Score (%)	ROC-AUC
No optimization	89.23 ± 4.21	88.57 ± 15.65	88.00 ± 10.95	86.67 ± 3.04	0.950 ± 0.050
Conventional GTO	89.23 ± 8.77	87.67 ± 11.64	84.00 ± 16.73	85.29 ± 12.61	0.925 ± 0.055
GTO + adaptive Lévy flight	89.23 ± 8.77	86.00 ± 14.22	88.00 ± 10.95	86.51 ± 10.48	0.930 ± 0.052
Proposed ALGTO	90.77 ± 3.44	89.33 ± 9.83	88.00 ± 10.95	87.92 ± 4.54	0.950 ± 0.035

**Table 9:** Comparative evaluation of hyperparameter optimization strategies under a matched evaluation budget.

Optimization Strategy	Best Validation Loss (Mean ± SD)	95% CI	Mean Optimization Time per Run (min)
Random Search	0.1844 ± 0.0044	0.1789–0.1899	1520
Grid Search	0.1762 ± 0.0026	0.1730–0.1794	1545
Bayesian Optimization	0.1618 ± 0.0039	0.1569–0.1667	1575
Conventional GTO	0.1702 ± 0.0026	0.1670–0.1735	1530
GTO + Adaptive Lévy Flight	0.1562 ± 0.0029	0.1526–0.1598	1535
Proposed ALGTO	0.1416 ± 0.0025	0.1385–0.1447	1540

**Table 10:** Hyperparameter sensitivity analysis on the development set (case-grouped five-fold cross-validation, mean ± SD).

Hyperparameter	Setting	Accuracy (%)	Precision (%)	Recall (%)	F1-Score (%)
Learning rate	0.00001	86.15 ± 3.44	88.00 ± 10.95	76.00 ± 8.94	80.78 ± 5.02
	**0.00005**	**90.77 ± 3.44**	**89.33 ± 9.83**	**88.00 ± 10.95**	**87.92 ± 4.54**
	0.0001	87.69 ± 4.21	88.00 ± 10.95	80.00 ± 0.00	83.56 ± 4.87
	0.0005	80.00 ± 4.21	79.33 ± 12.51	68.00 ± 10.95	72.21 ± 5.71
	0.001	76.92 ± 5.44	75.33 ± 15.16	64.00 ± 8.94	68.21 ± 5.89
Batch size	8	87.69 ± 4.21	82.95 ± 10.50	88.00 ± 10.95	84.63 ± 5.05
	**16**	**90.77 ± 3.44**	**89.33 ± 9.83**	**88.00 ± 10.95**	**87.92 ± 4.54**
	32	87.69 ± 4.21	88.00 ± 10.95	80.00 ± 0.00	83.56 ± 4.87
Weight decay	0.00001	83.08 ± 6.44	80.33 ± 12.27	76.00 ± 8.94	77.66 ± 8.40
	0.00005	87.69 ± 4.21	88.00 ± 10.95	80.00 ± 0.00	83.56 ± 4.87
	**0.0001**	**90.77 ± 3.44**	**89.33 ± 9.83**	**88.00 ± 10.95**	**87.92 ± 4.54**
	0.0005	80.00 ± 4.21	77.67 ± 13.72	72.00 ± 10.95	73.42 ± 4.81
	0.001	80.00 ± 4.21	79.33 ± 12.51	68.00 ± 10.95	72.21 ± 5.71
Dropout rate	0.10	83.08 ± 6.44	80.33 ± 12.27	76.00 ± 8.94	77.66 ± 8.40
	0.20	87.69 ± 4.21	88.00 ± 10.95	80.00 ± 0.00	83.56 ± 4.87
	**0.30**	**90.77 ± 3.44**	**89.33 ± 9.83**	**88.00 ± 10.95**	**87.92 ± 4.54**
	0.35	87.69 ± 4.21	88.00 ± 10.95	80.00 ± 0.00	83.56 ± 4.87
	0.40	83.08 ± 6.44	80.33 ± 12.27	76.00 ± 8.94	77.66 ± 8.40
	0.50	81.54 ± 6.88	79.33 ± 12.51	72.00 ± 10.95	74.99 ± 9.51

Note: Bold values denote the ALGTO-selected configuration.

**Table 11:** Performance comparison with baseline models on the development set (case-grouped five-fold cross-validation; mean ± SD, with 95% Wilson score confidence intervals for pooled out-of-fold accuracy).

Model	Accuracy (%)	95% CI	Precision (%)	Recall (%)	F1-Score (%)	ROC-AUC
VGG16	67.69 ± 3.44	55.61–77.80	56.86 ± 4.09	68.00 ± 10.95	61.58 ± 5.15	0.805 ± 0.060
ResNet50	76.92 ± 5.44	65.36–85.49	68.00 ± 7.30	76.00 ± 8.94	71.64 ± 7.23	0.850 ± 0.055
DenseNet121	83.08 ± 3.44	72.18–90.28	75.62 ± 6.23	84.00 ± 8.94	79.21 ± 3.90	0.900 ± 0.045
EfficientNet-B0	86.15 ± 3.44	75.73–92.54	78.95 ± 4.45	88.00 ± 10.95	82.85 ± 4.73	0.930 ± 0.035
Proposed framework	90.77 ± 3.44	81.29–95.70	89.33 ± 9.83	88.00 ± 10.95	87.92 ± 4.54	0.950 ± 0.035

**Table 12:** Paired statistical comparison of the proposed framework and baseline models on the out-of-fold predictions of the development set (65 case groups).

Comparison	Proposed Correct	Baseline Correct	Baseline Wins (b)	Proposed Wins (c)	Both Correct	Both Wrong	McNemar’s Exact p
Proposed vs. VGG16	59/65	44/65	3	18	41	3	0.0015
Proposed vs. ResNet50	59/65	50/65	3	12	47	3	0.0352
Proposed vs. DenseNet121	59/65	54/65	4	9	50	2	0.2668
Proposed vs. EfficientNet-B0	59/65	56/65	4	7	52	2	0.5488

**Table 13:** Performance comparison of the proposed framework with reported results from existing published methods.

Author	Method	Accuracy (%)
Ayoub et al. [23]	ViT + Convolutional Layers	87.51
Abdi et al. [11]	Explainable CNN	89.73
Inamdar et al. [16]	Dual-stream Multi-scale + Hybrid Attention	92.42
Bajaj et al. [14]	Ensemble CNN (ResNet + VGG19)	92.43
Jena et al. [15]	CNN + Bi-LSTM + DenseNet121	93.36
Proposed Model: ConvNeXt-Tiny–Vision Mamba + AHDA + ACAF + ALGTO (locked case-level test set)		94.12

## Data Availability

The dataset used in this study is publicly available. The brain stroke CT image dataset was obtained from the Kaggle repository *Brain Stroke Prediction CT Scan Image Dataset* by Iashiqul and can be accessed at https://www.kaggle.com/datasets/iashiqul/brain-stroke-prediction-ct-scan-image-dataset (accessed on 12 June 2026). The source code implementing the proposed framework is publicly available at: https://github.com/fathimabeevi121/Hybrid-ConvNeXt-Tiny-Vision-Mamba-Stroke-Classification (accessed on 12 June 2026).

## Supplementary Materials

The following supporting information can be downloaded at https://www.mdpi.com/article/10.3390/biomimetics11100709/s1, Table S1: Case-level split manifest, listing the case identifier, class label, and subset assignment for all 82 case groups used in this study; Table S2: Per-seed best validation loss for each hyperparameter optimization strategy across five random seeds under the matched budget of 96 model evaluations; Figure S1: Slice-level training and validation accuracy and loss curves for the proposed framework across the five case-grouped cross-validation folds of the development set, obtained using the fixed ALGTO-selected hyperparameter configuration: (a) Fold 1, (b) Fold 2, (c) Fold 3, (d) Fold 4, and (e) Fold 5; Figure S2: Fold-wise case-level confusion matrices of the component-based ablation across the five case-grouped cross-validation folds of the development set: (a–e) ConvNeXt-Tiny, (f–j) ConvNeXt-Tiny + Vision Mamba, (k–o) ConvNeXt-Tiny + Vision Mamba + AHDA, and (p–t) ConvNeXt-Tiny + Vision Mamba + AHDA + ACAF; Figure S3: Fold-wise case-level confusion matrices of the optimization-based ablation across the five case-grouped cross-validation folds of the development set for the ConvNeXt-Tiny + Vision Mamba + AHDA + ACAF architecture: (a–e) no optimization, (f–j) conventional GTO, (k–o) GTO with adaptive Lévy flight, and (p–t) proposed ALGTO.
