# Supplementary material for: Hybrid ConvNeXt–Vision Mamba with Hemispheric Difference-Aware Attention and Bioinspired Optimization for Stroke Classification
Source: Biomimetics (Basel). 2026 Oct 4;11(10):709. doi: 10.3390/biomimetics11100709 (PMC13702623; doi:10.3390/biomimetics11100709)
Supplement: Supplementary file 1 [file biomimetics-11-00709-s001.zip › biomimetics-4548901-supplementary.pdf]

Table S1. Case-level split manifest, listing the case identifier, class label, and subset assignment for all 82 case groups used in this study.

Case ID	Class label	Subset
50	Normal	Test
57	Normal	Test
59	Normal	Test
65	Normal	Test
96	Normal	Test
98	Normal	Test
104	Normal	Test
121	Normal	Test
123	Normal	Test
125	Normal	Test
128	Normal	Test
70	Stroke	Test
76	Stroke	Test
79	Stroke	Test
80	Stroke	Test
87	Stroke	Test
90	Stroke	Test
49	Normal	Train
51	Normal	Train
52	Normal	Train
54	Normal	Train
55	Normal	Train
56	Normal	Train
61	Normal	Train
62	Normal	Train
63	Normal	Train
64	Normal	Train
95	Normal	Train
99	Normal	Train
100	Normal	Train
102	Normal	Train
103	Normal	Train
105	Normal	Train
106	Normal	Train
107	Normal	Train
108	Normal	Train
109	Normal	Train
111	Normal	Train
112	Normal	Train
113	Normal	Train
114	Normal	Train
115	Normal	Train
116	Normal	Train
117	Normal	Train
118	Normal	Train
119	Normal	Train
120	Normal	Train

122	Normal	Train
126	Normal	Train
127	Normal	Train
129	Normal	Train
130	Normal	Train
58	Stroke	Train
67	Stroke	Train
68	Stroke	Train
69	Stroke	Train
71	Stroke	Train
72	Stroke	Train
73	Stroke	Train
75	Stroke	Train
78	Stroke	Train
81	Stroke	Train
82	Stroke	Train
83	Stroke	Train
84	Stroke	Train
85	Stroke	Train
86	Stroke	Train
88	Stroke	Train
89	Stroke	Train
91	Stroke	Train
92	Stroke	Train
93	Stroke	Train
94	Stroke	Train
97	Stroke	Train
53	Normal	Validation
60	Normal	Validation
101	Normal	Validation
110	Normal	Validation
124	Normal	Validation
66	Stroke	Validation
74	Stroke	Validation
77	Stroke	Validation

Table S2. Per-seed best validation loss for each hyperparameter optimization strategy across five random seeds under the matched budget of 96 model evaluations.

Optimization Strategy	Seed 1	Seed 2	Seed 3	Seed 4	Seed 5
Random Search	0.1790	0.1815	0.1840	0.1875	0.1900
Grid Search	0.1728	0.1748	0.1762	0.1776	0.1796
Bayesian Optimization	0.1575	0.1592	0.1612	0.1635	0.1676
Conventional GTO	0.1670	0.1685	0.1702	0.1720	0.1735
GTO + Adaptive Lévy Flight	0.1525	0.1545	0.1562	0.1580	0.1598
Proposed ALGTO	0.1385	0.1400	0.1415	0.1430	0.1450

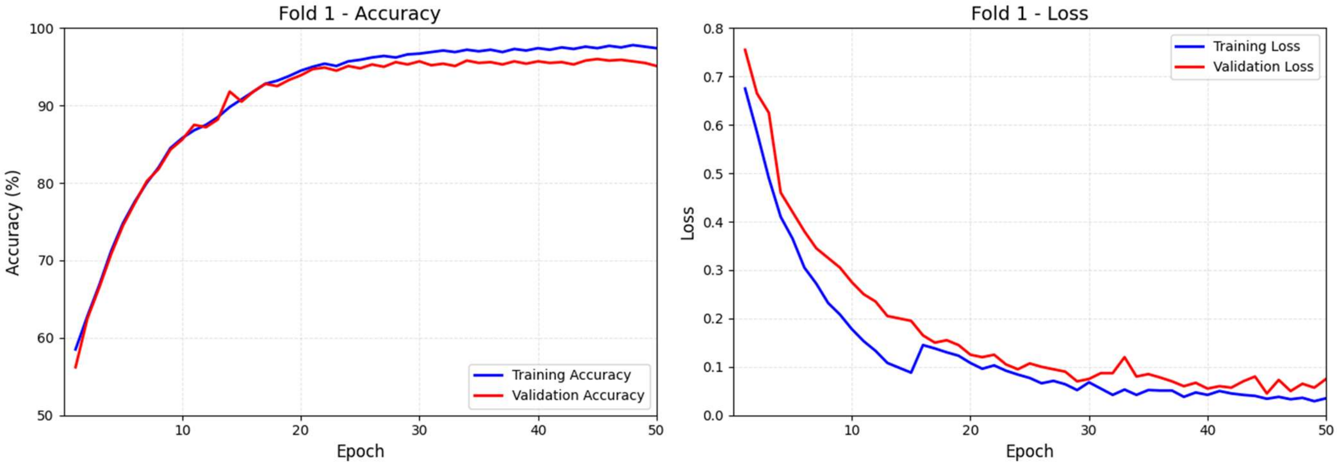

(a)

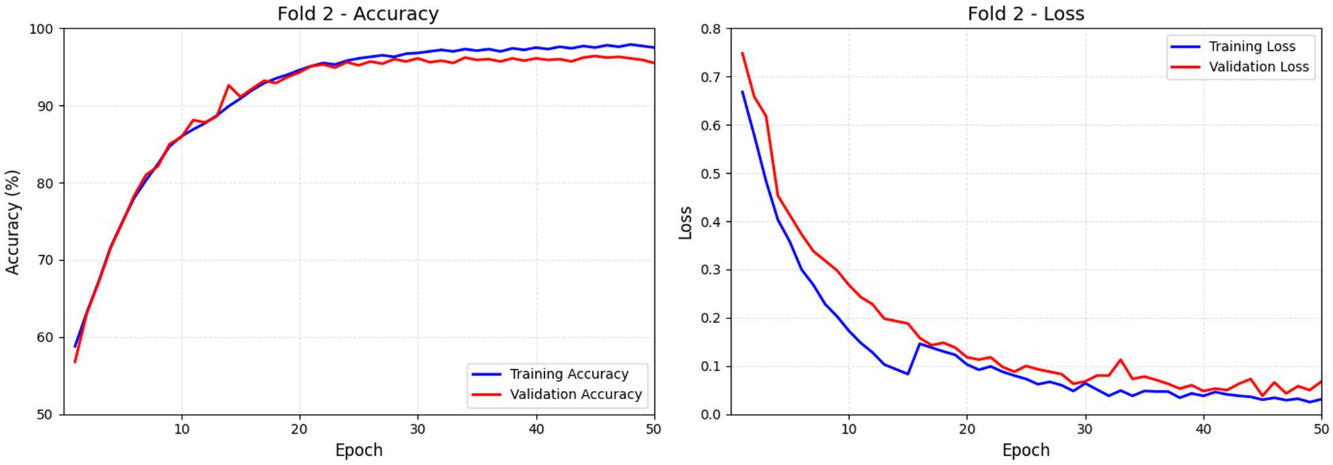

(b)

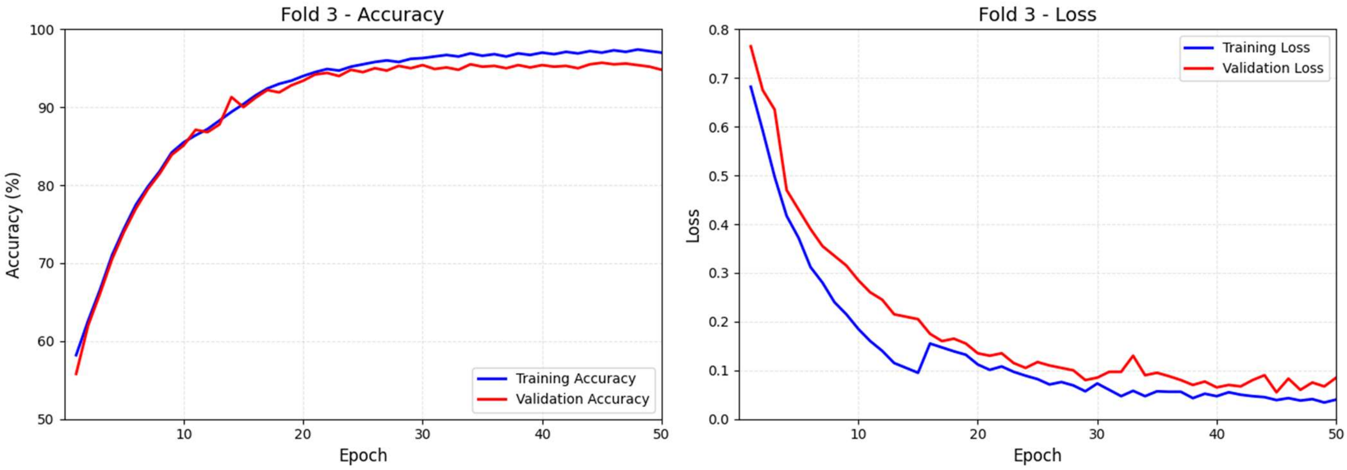

(c)

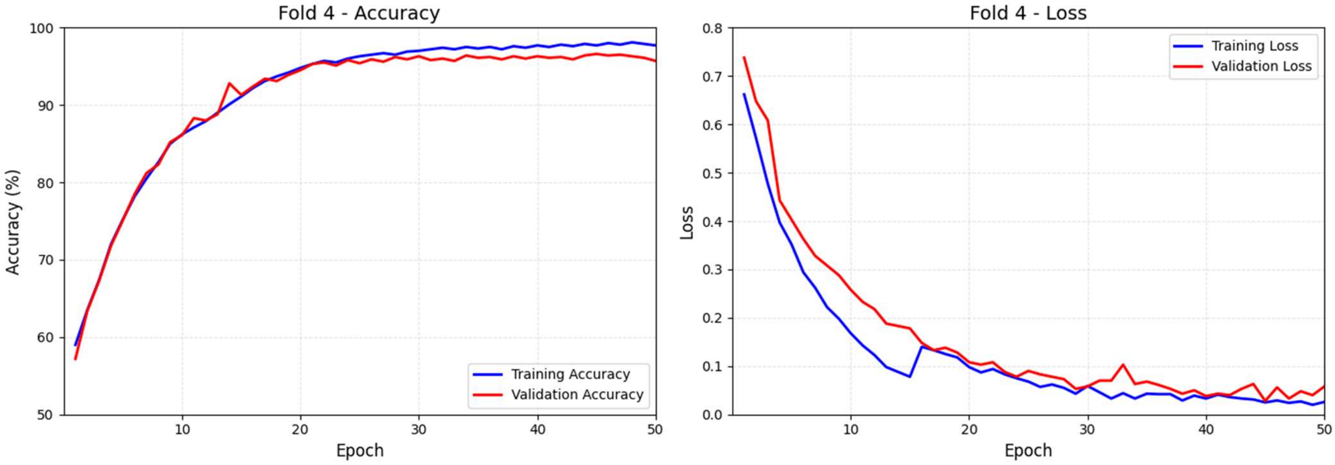

(d)

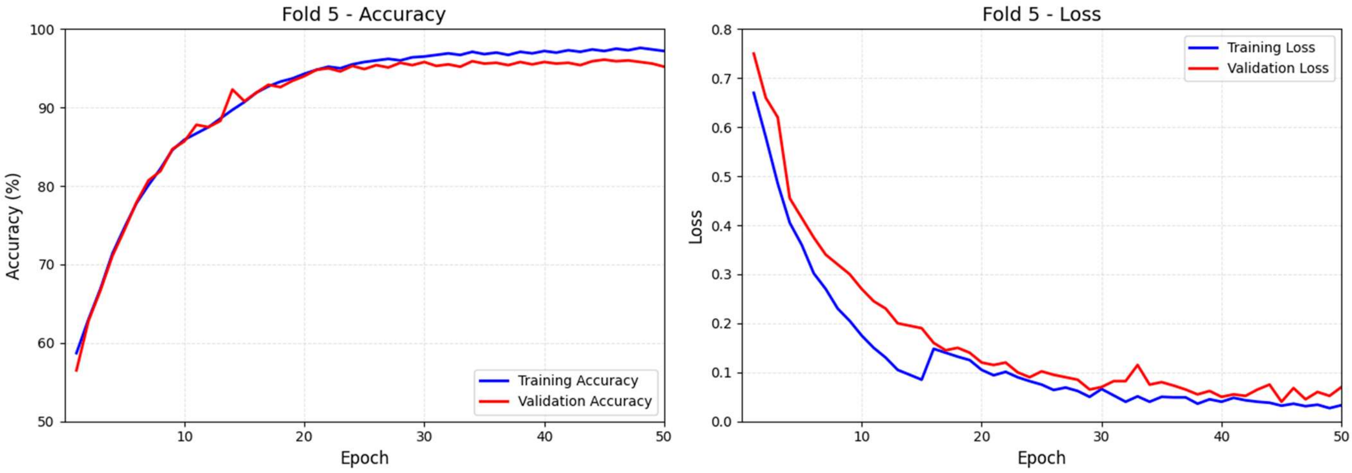

(e)

Figure S1. Slice-level training and validation accuracy and loss curves for the proposed framework across the five case-grouped cross-validation folds of the development set, obtained using the fixed ALGTO-selected hyperparameter configuration: (a) Fold 1, (b) Fold 2, (c) Fold 3, (d) Fold 4, and (e) Fold 5.

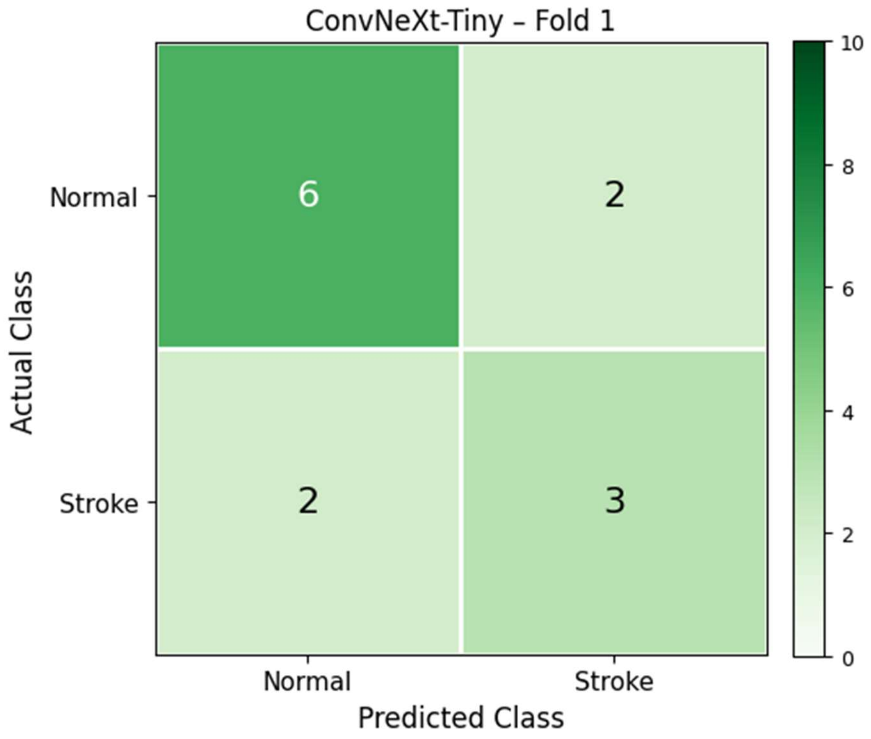
ConvNeXt-Tiny – Fold 1
Actual Class
Normal
Stroke
6
2
2
3
Normal
Stroke
Predicted Class
10
8
6
4
2
0

(a)

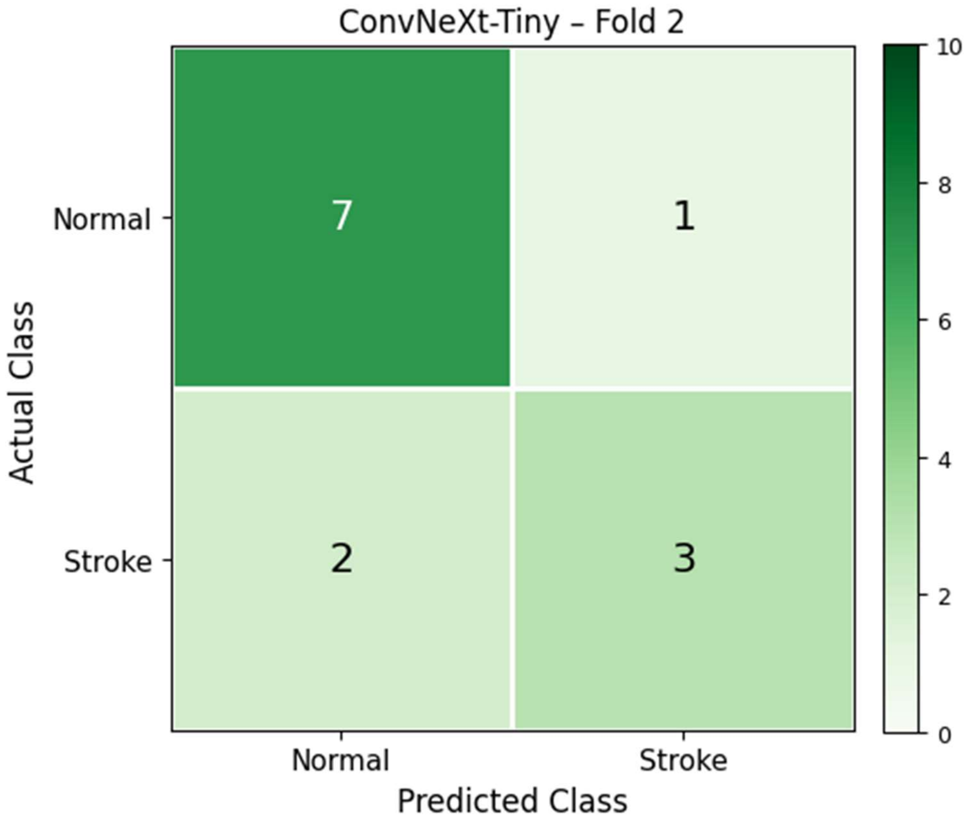
ConvNeXt-Tiny – Fold 2
Actual Class
Normal
Stroke
7
1
2
3
Normal
Stroke
Predicted Class
10
8
6
4
2
0

(b)

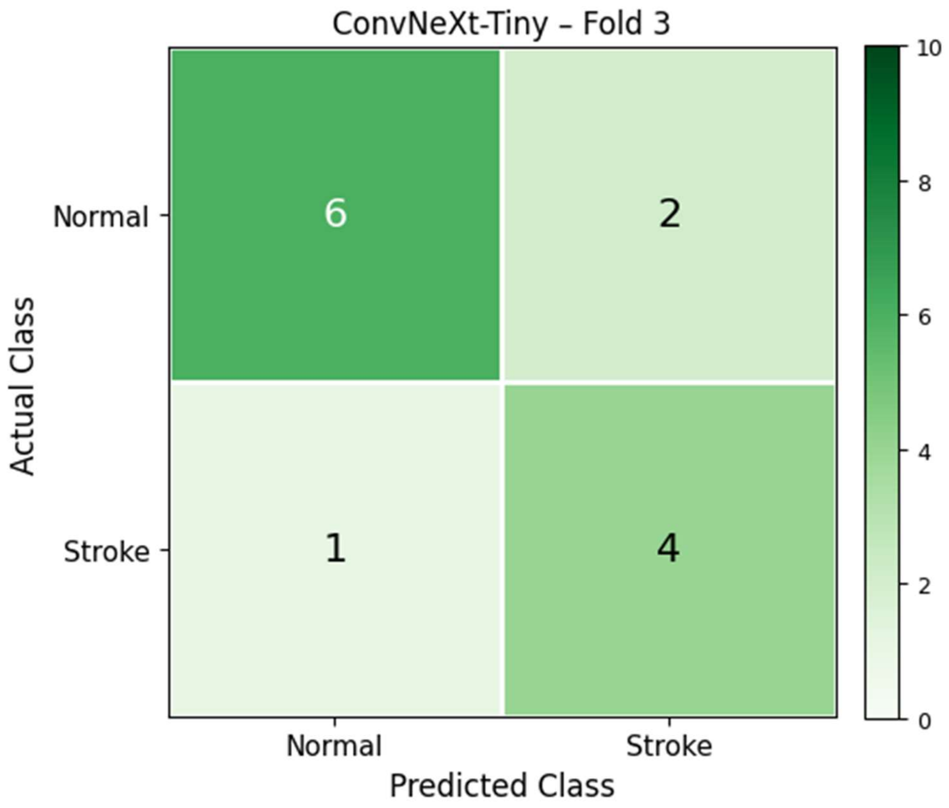
ConvNeXt-Tiny – Fold 3
Normal
Stroke
Actual Class
6
2
1
4
Normal
Stroke
Predicted Class
10
8
6
4
2
0

(c)

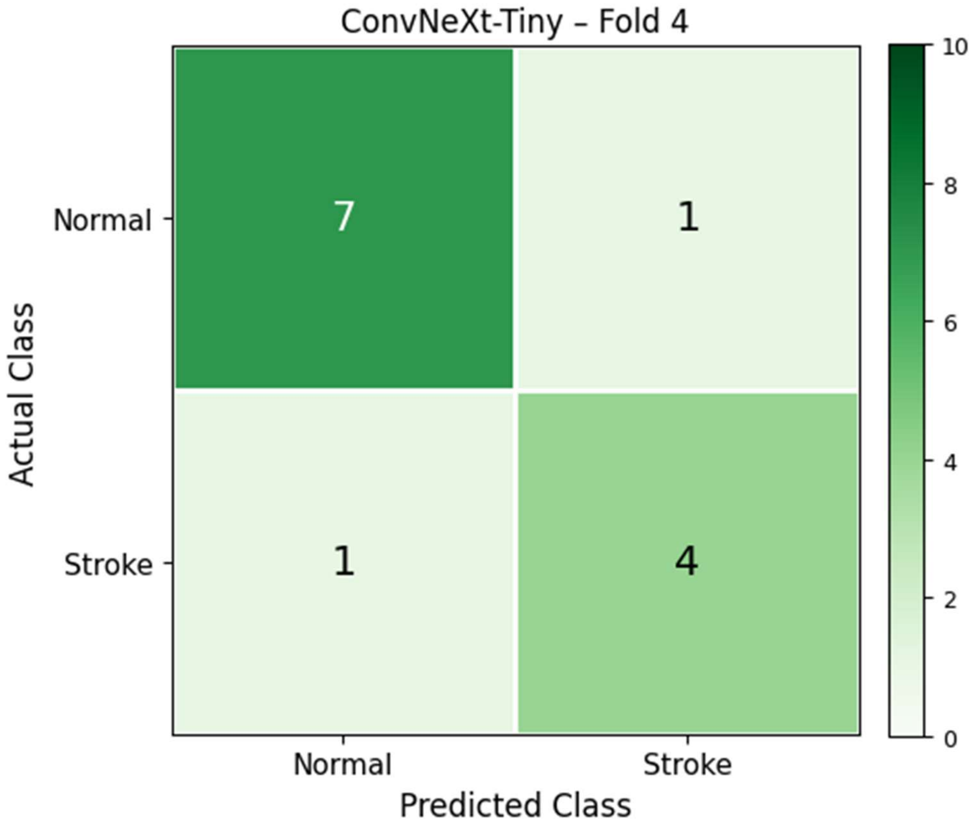
ConvNeXt-Tiny – Fold 4
Normal
Stroke
Actual Class
7
1
1
4
Normal
Stroke
Predicted Class
10
8
6
4
2
0

(d)

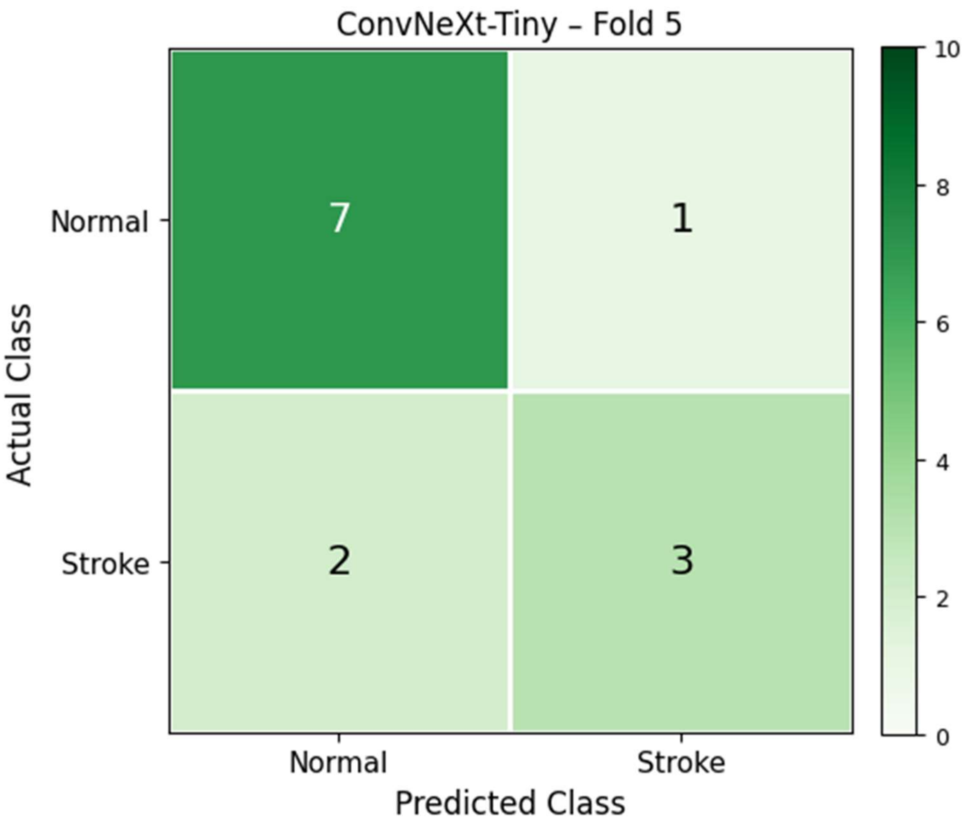

ConvNeXt-Tiny – Fold 5
Normal
Stroke
Actual Class
7
1
2
3
Normal
Stroke
Predicted Class
10
8
6
4
2
0

(e)

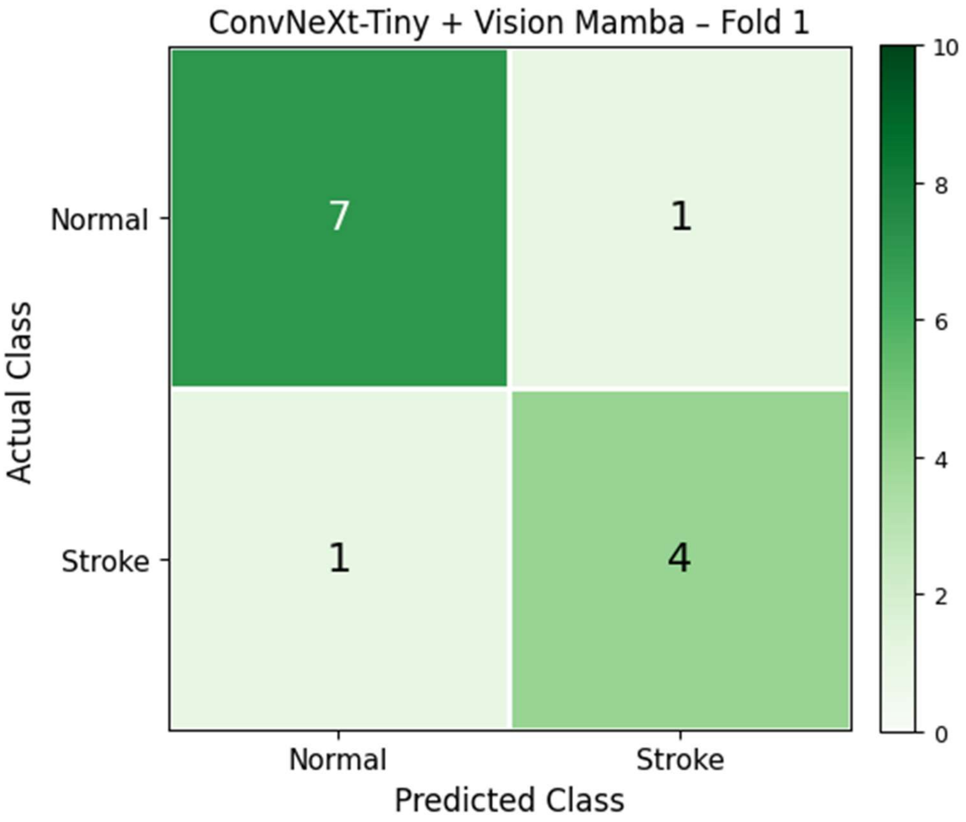

ConvNeXt-Tiny + Vision Mamba – Fold 1
Normal
Stroke
Actual Class
7
1
1
4
Normal
Stroke
Predicted Class
10
8
6
4
2
0

(f)

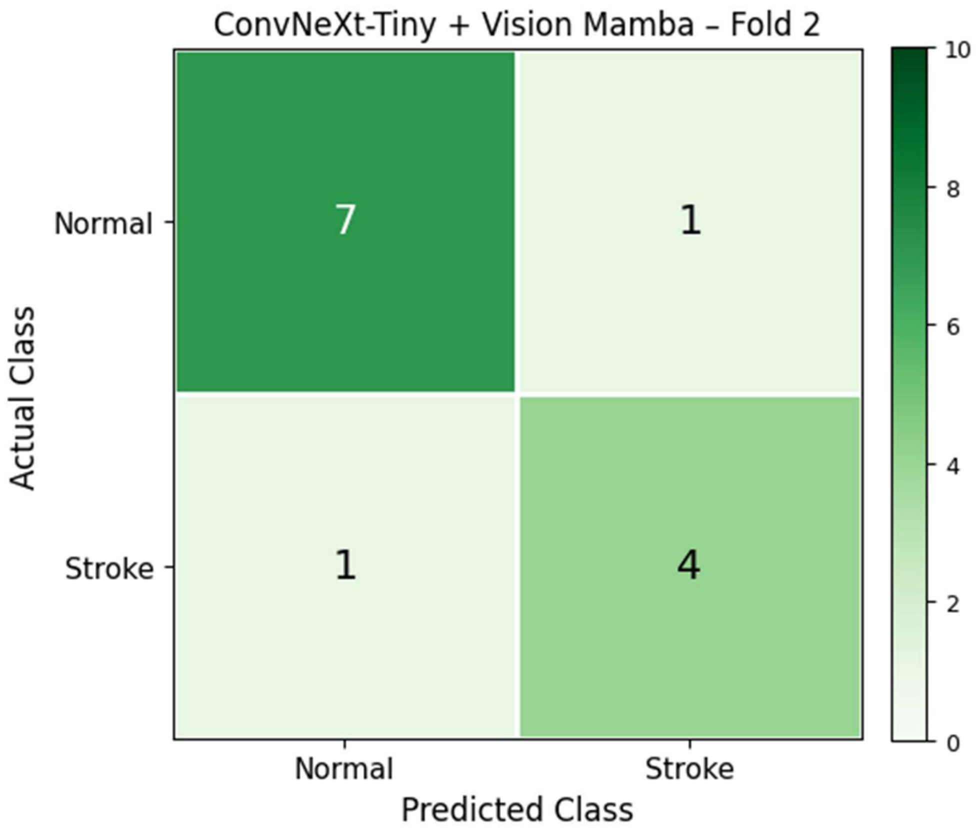
ConvNeXt-Tiny + Vision Mamba – Fold 2
Actual Class
Normal
Stroke
7
1
1
4
Predicted Class
Normal
Stroke
0
2
4
6
8
10
(g)

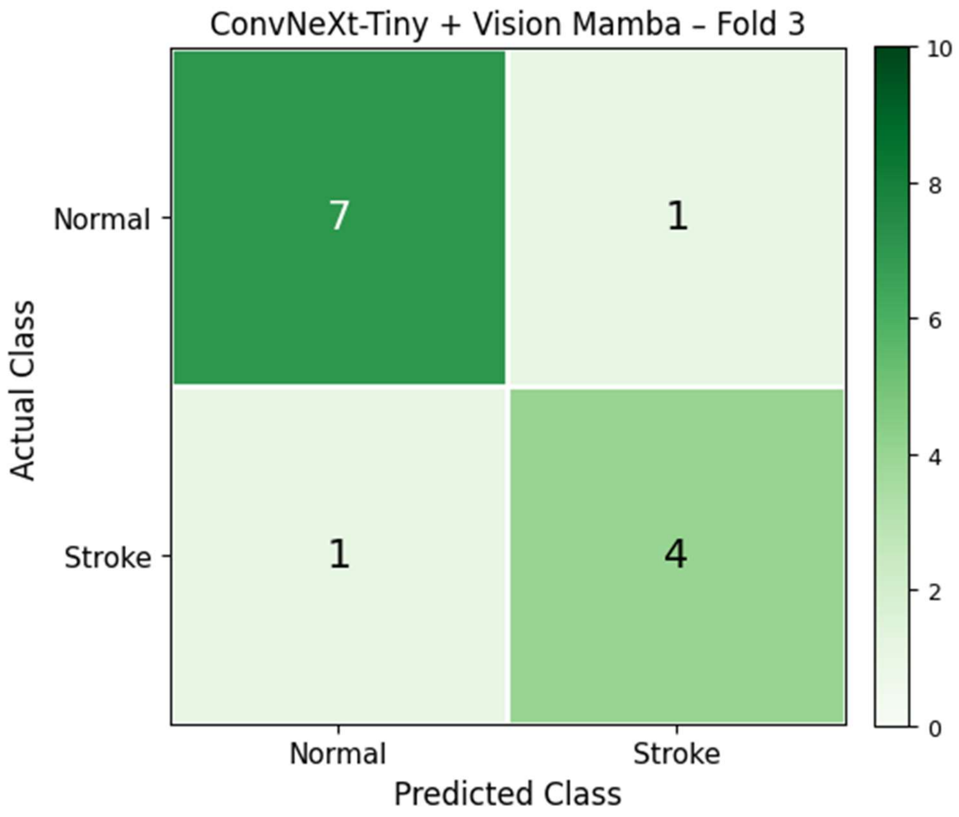
ConvNeXt-Tiny + Vision Mamba – Fold 3
Actual Class
Normal
Stroke
7
1
1
4
Predicted Class
Normal
Stroke
0
2
4
6
8
10
(h)

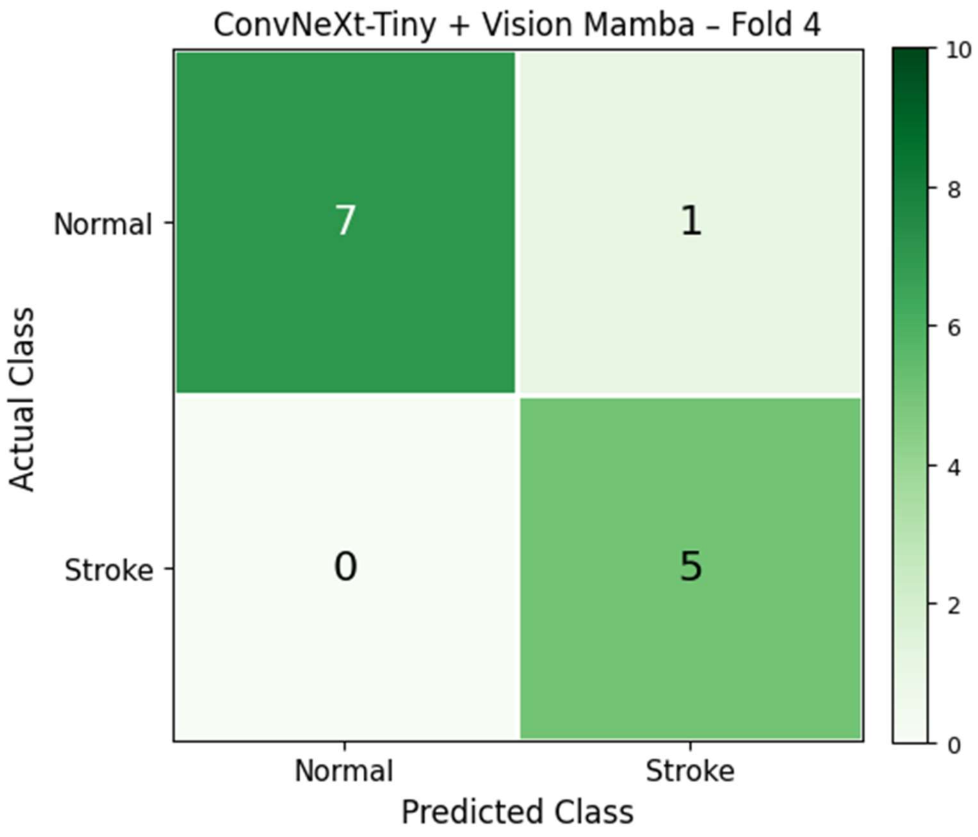
ConvNeXt-Tiny + Vision Mamba – Fold 4
Normal
Stroke
Actual Class
7
1
0
5
Normal
Stroke
Predicted Class
10
8
6
4
2
0

(i)

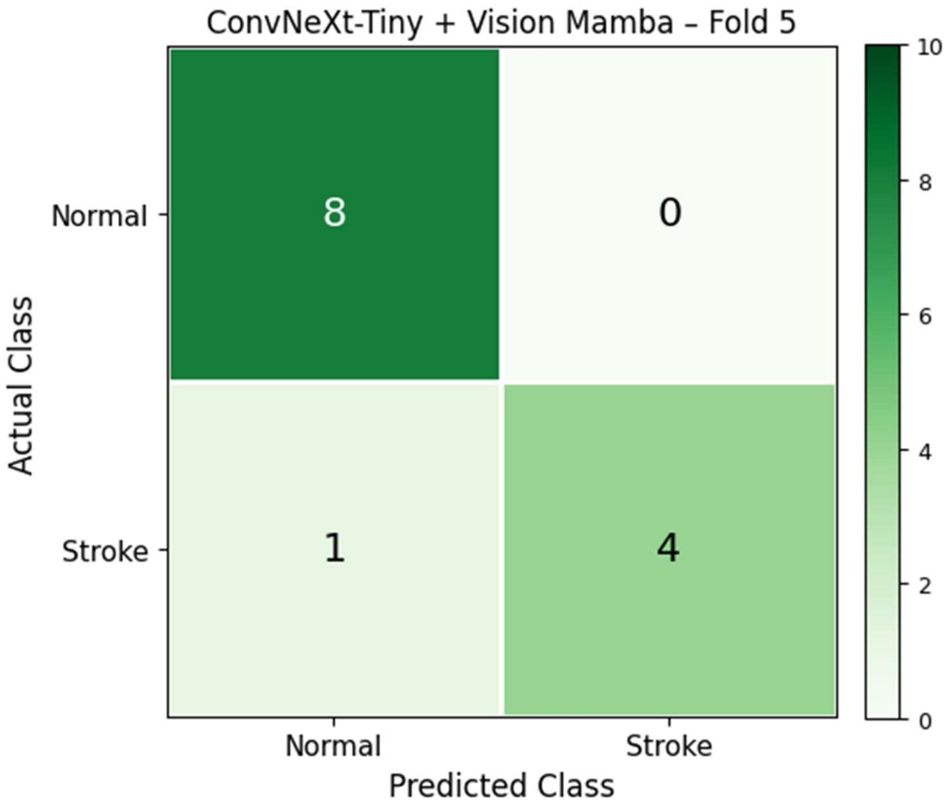
ConvNeXt-Tiny + Vision Mamba – Fold 5
Normal
Stroke
Actual Class
8
0
1
4
Normal
Stroke
Predicted Class
10
8
6
4
2
0

(j)

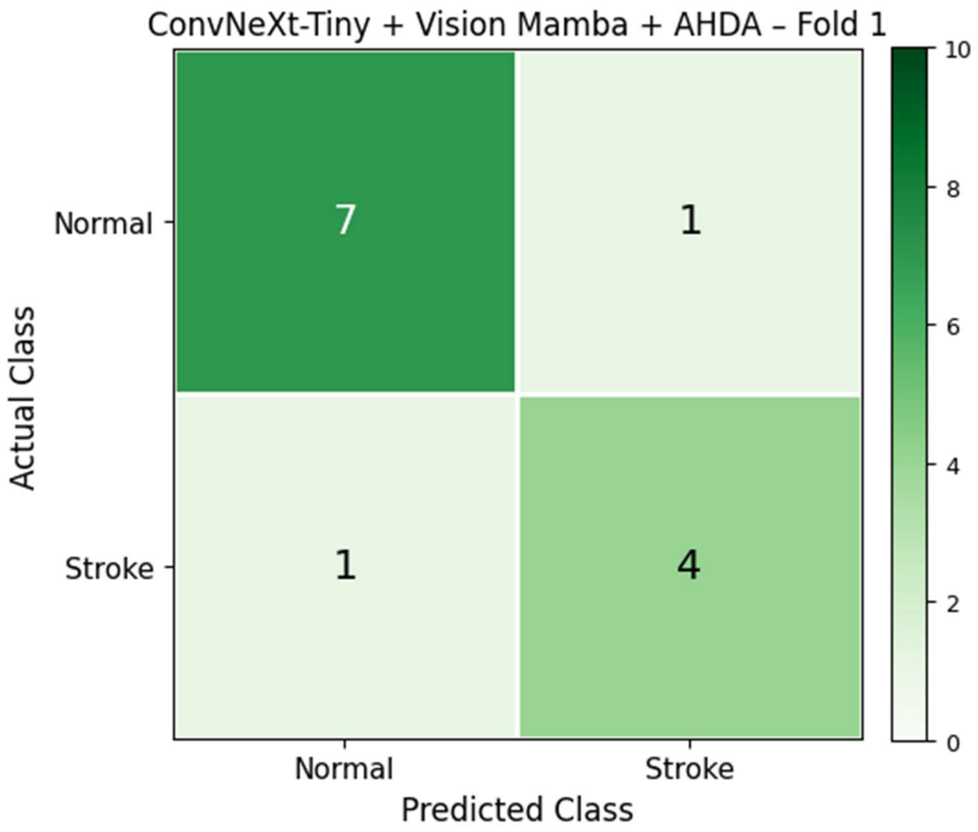
ConvNeXt-Tiny + Vision Mamba + AHDA – Fold 1
Actual Class
Normal
Stroke
7
1
1
4
Predicted Class
Normal
Stroke
0
2
4
6
8
10

(k)

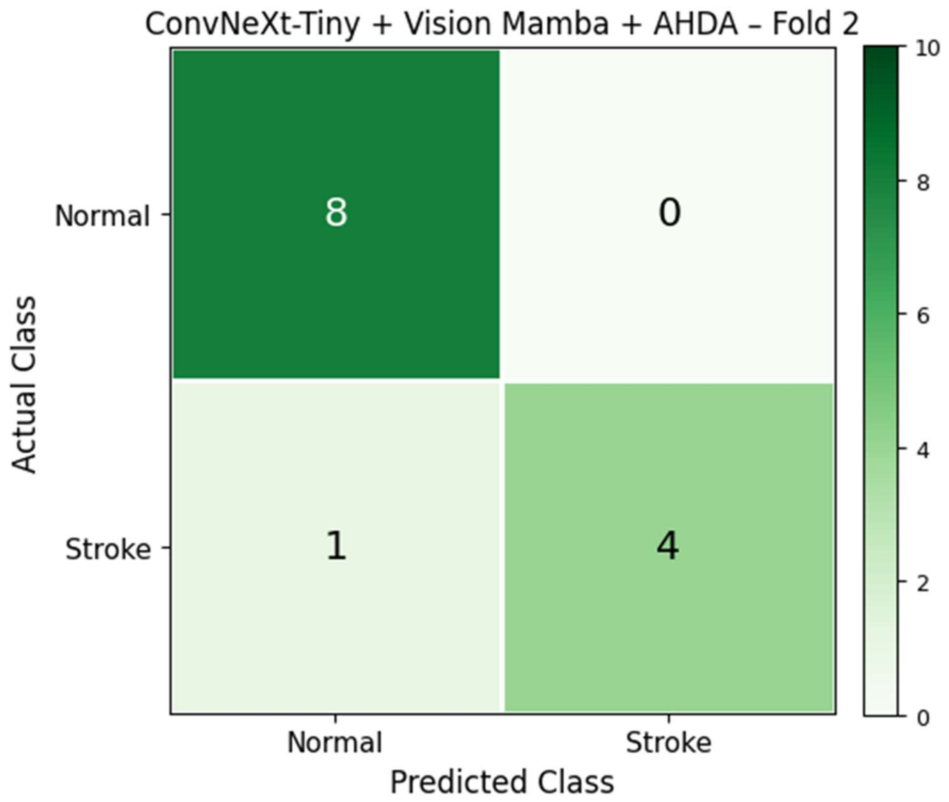
ConvNeXt-Tiny + Vision Mamba + AHDA – Fold 2
Actual Class
Normal
Stroke
8
0
1
4
Predicted Class
Normal
Stroke
0
2
4
6
8
10

(l)

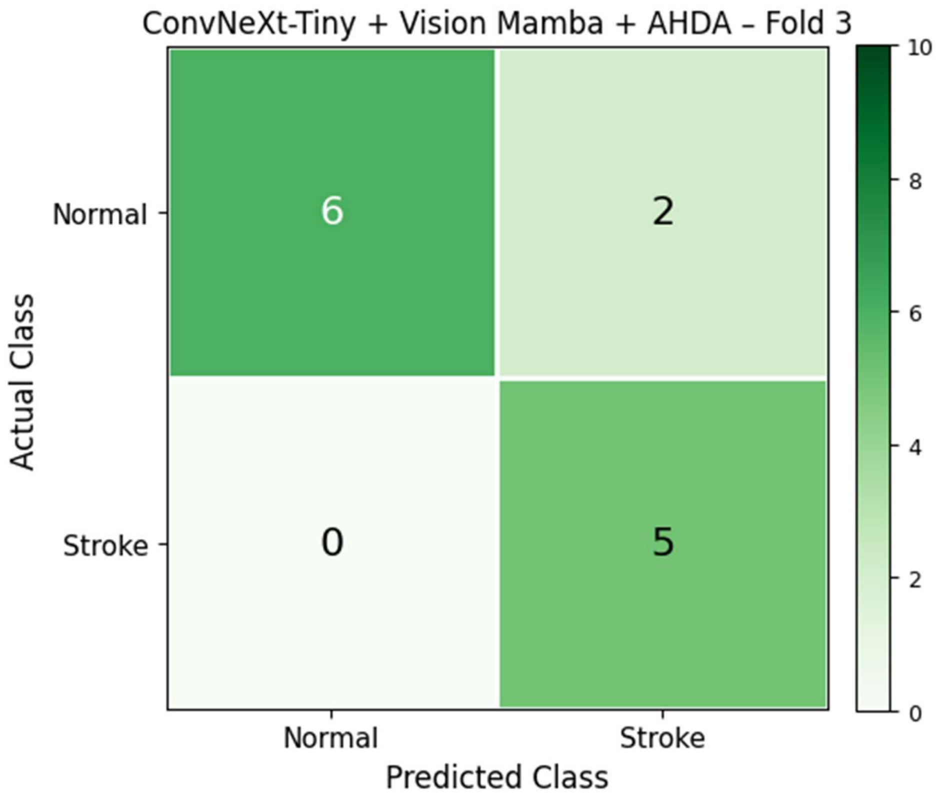
ConvNeXt-Tiny + Vision Mamba + AHDA – Fold 3
Actual Class
Normal
Stroke
6
2
0
5
Normal
Stroke
Predicted Class
0
2
4
6
8
10

(m)

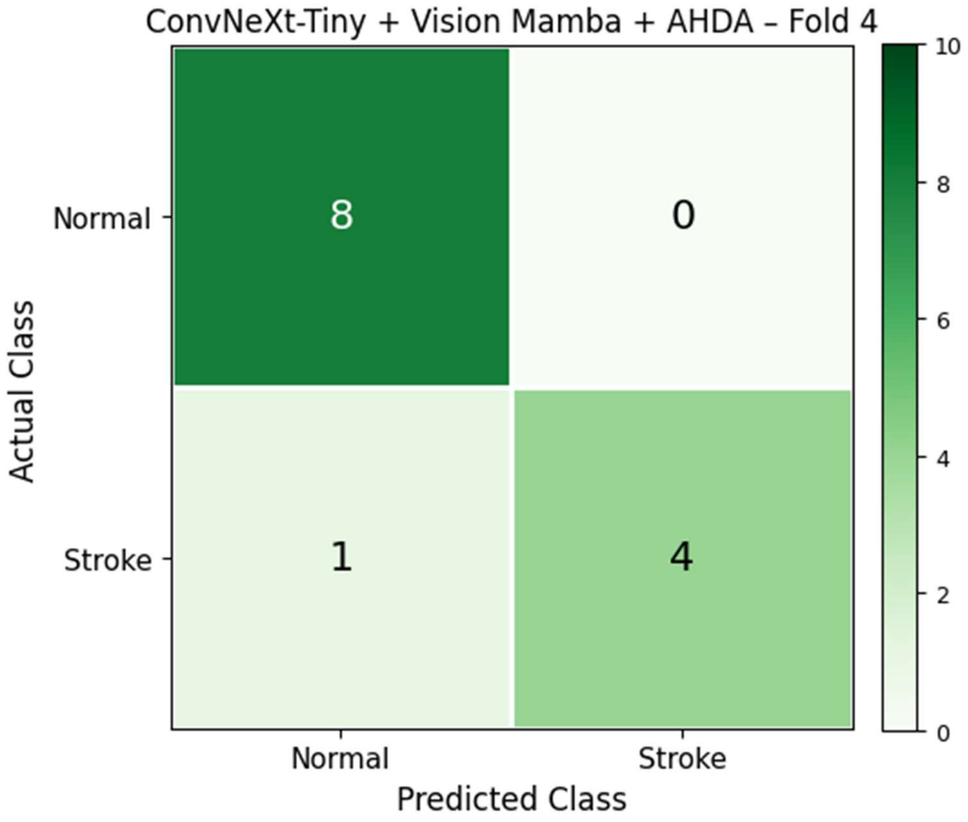
ConvNeXt-Tiny + Vision Mamba + AHDA – Fold 4
Actual Class
Normal
Stroke
8
0
1
4
Normal
Stroke
Predicted Class
0
2
4
6
8
10

(n)

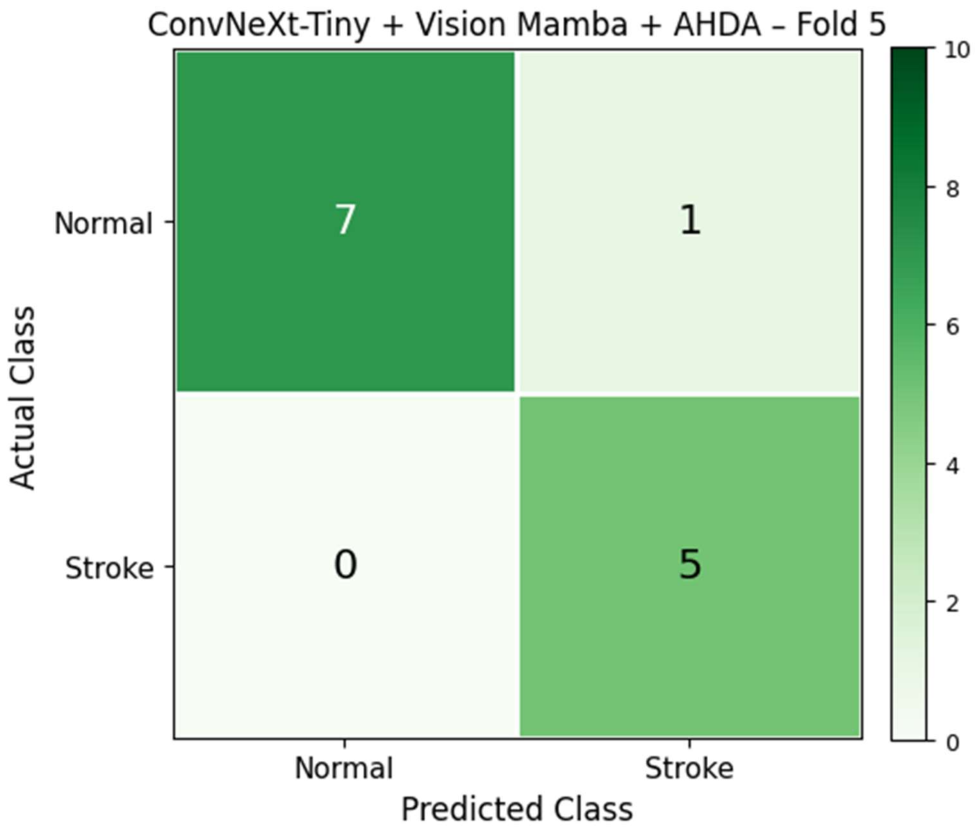
ConvNeXt-Tiny + Vision Mamba + AHDA – Fold 5
Normal
Stroke
Actual Class
7
1
0
5
Normal
Stroke
Predicted Class
10
8
6
4
2
0

(o)

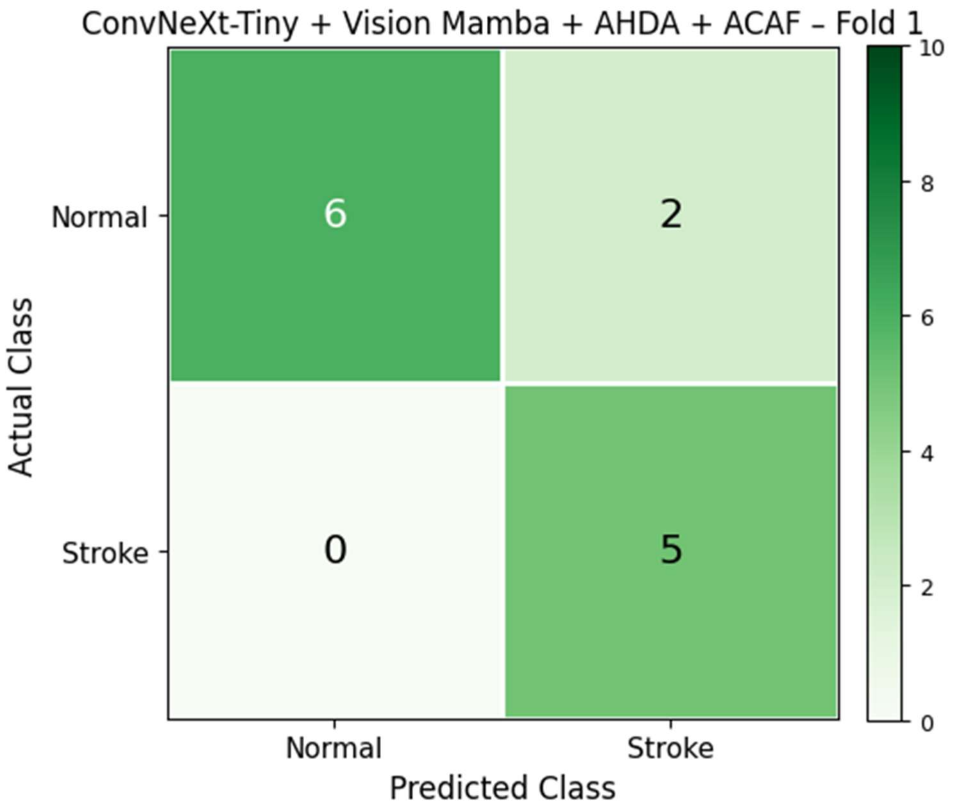
ConvNeXt-Tiny + Vision Mamba + AHDA + ACAF – Fold 1
Normal
Stroke
Actual Class
6
2
0
5
Normal
Stroke
Predicted Class
10
8
6
4
2
0

(p)

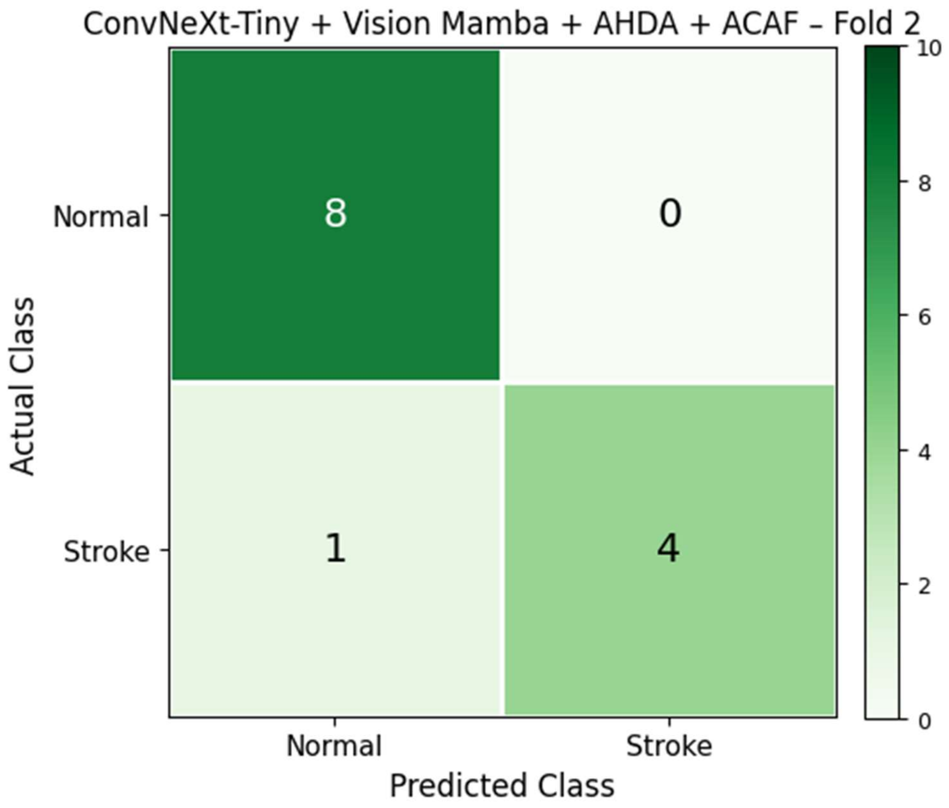

ConvNeXt-Tiny + Vision Mamba + AHDA + ACAF – Fold 2
Actual Class
Normal
Stroke
8
0
1
4
Normal
Stroke
Predicted Class
10
8
6
4
2
0

(q)

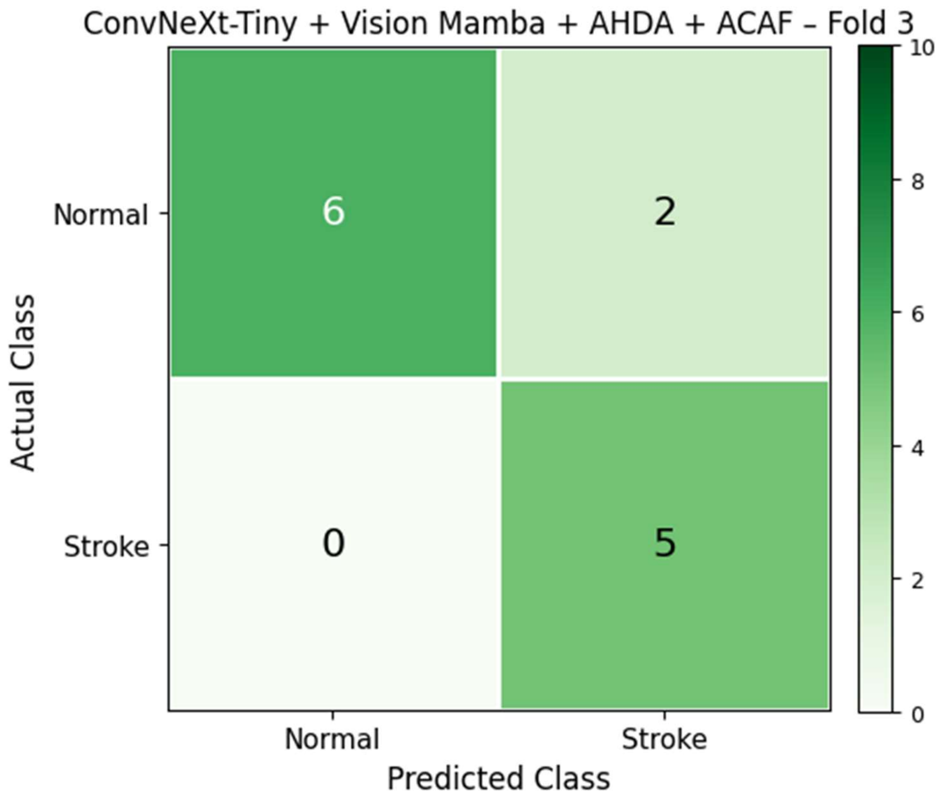

ConvNeXt-Tiny + Vision Mamba + AHDA + ACAF – Fold 3
Actual Class
Normal
Stroke
6
2
0
5
Normal
Stroke
Predicted Class
10
8
6
4
2
0

(r)

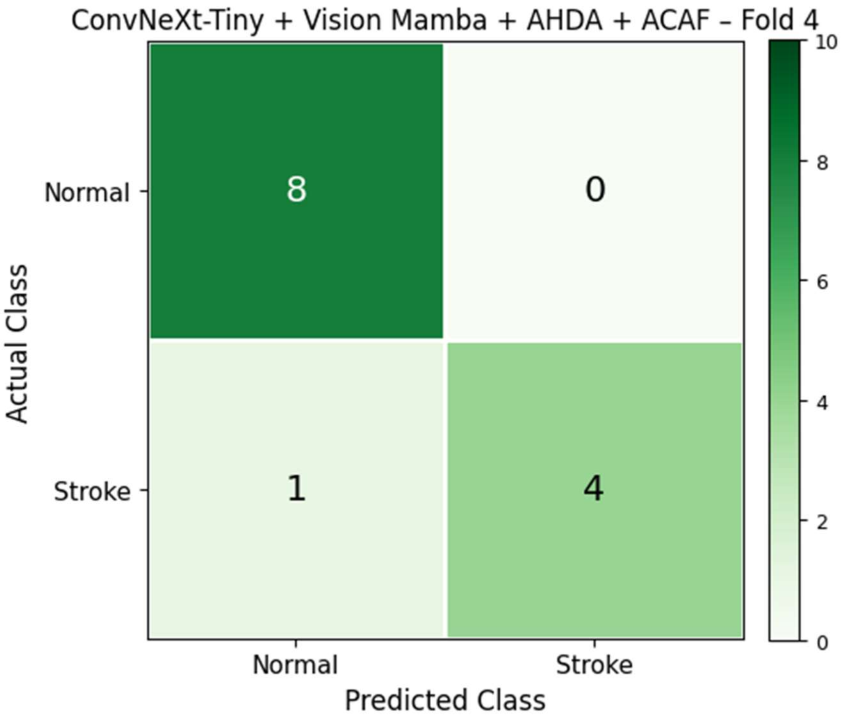

(s)

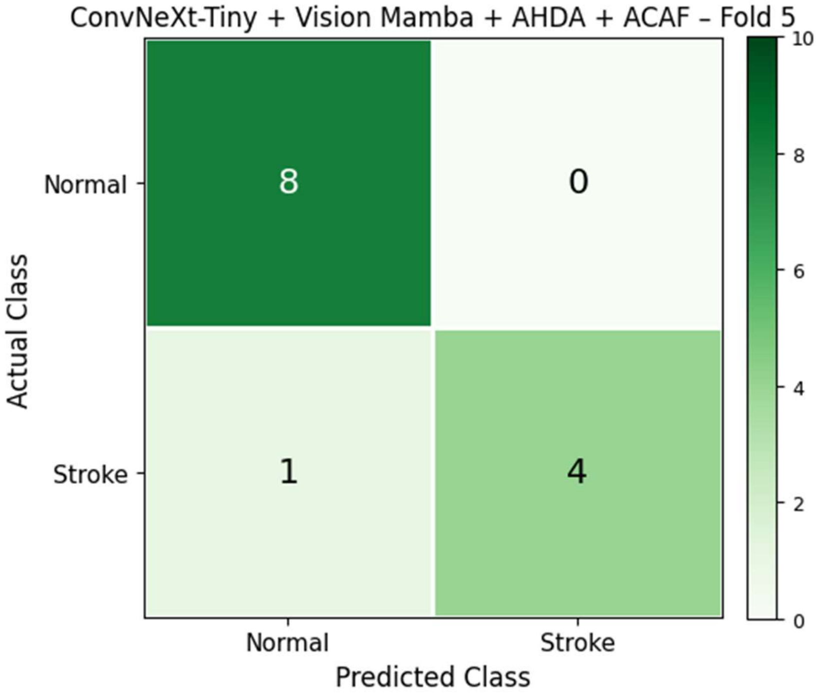

(t)

Figure S2. Fold-wise case-level confusion matrices of the component-based ablation across the five case-grouped cross-validation folds of the development set: (a–e) ConvNeXt-Tiny, (f–j) ConvNeXt-Tiny + Vision Mamba, (k–o) ConvNeXt-Tiny + Vision Mamba + AHDA, and (p–t) ConvNeXt-Tiny + Vision Mamba + AHDA + ACAF.

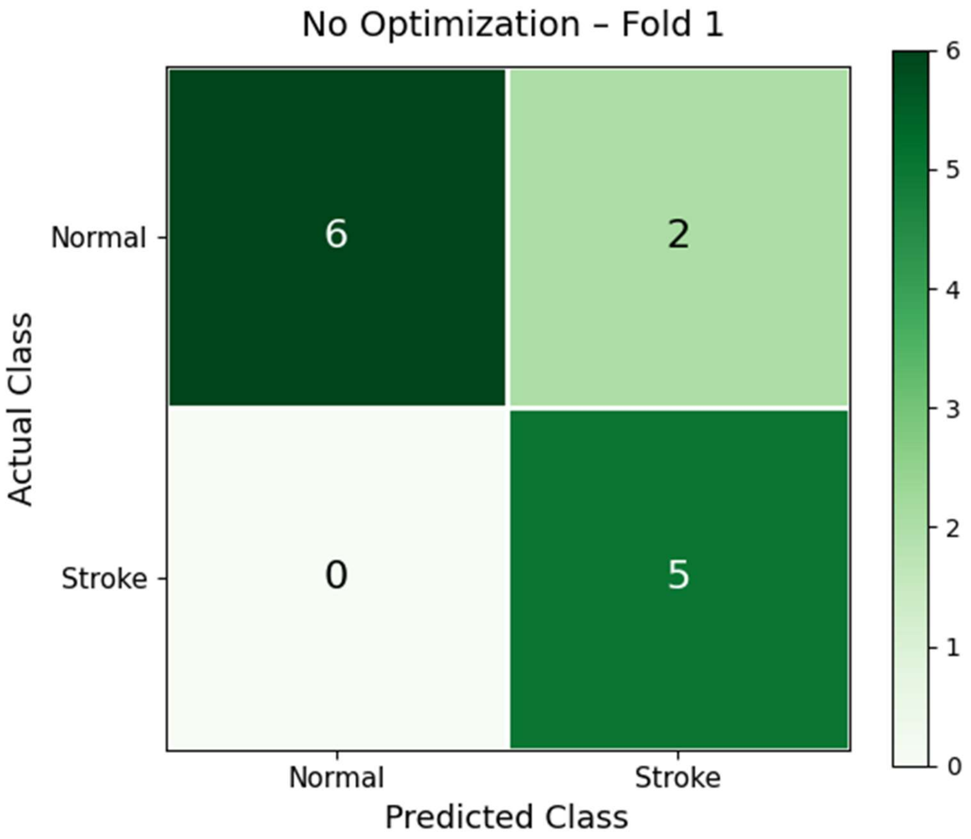
No Optimization – Fold 1
Actual Class
Normal
Stroke
6
2
0
5
Normal
Stroke
Predicted Class
0
1
2
3
4
5
6

(a)

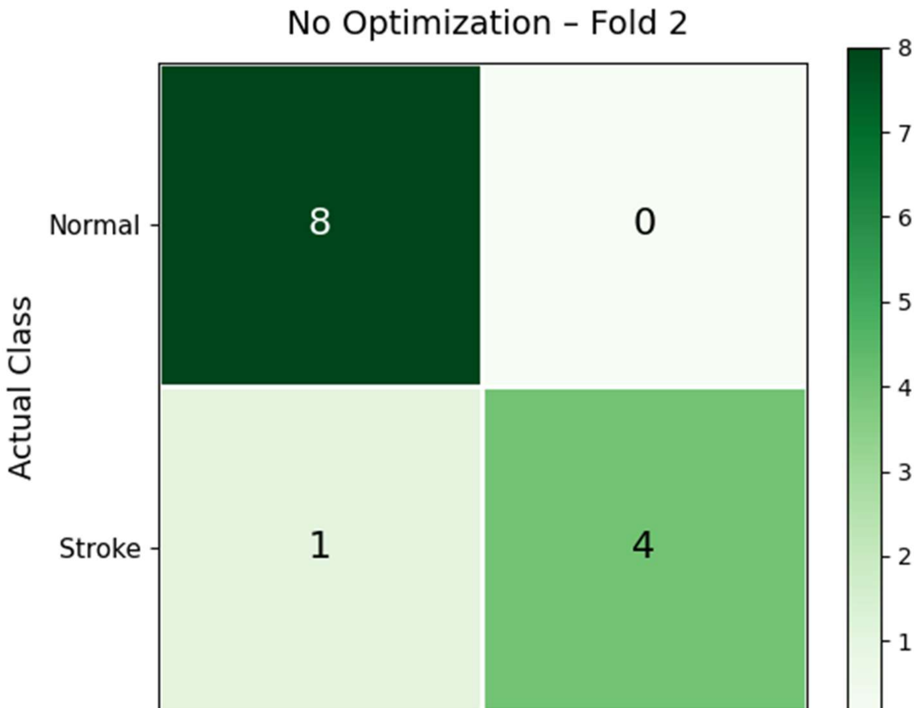
No Optimization – Fold 2
Actual Class
Normal
Stroke
8
0
1
4
Normal
Stroke
Predicted Class
0
1
2
3
4
5
6
7
8

(b)

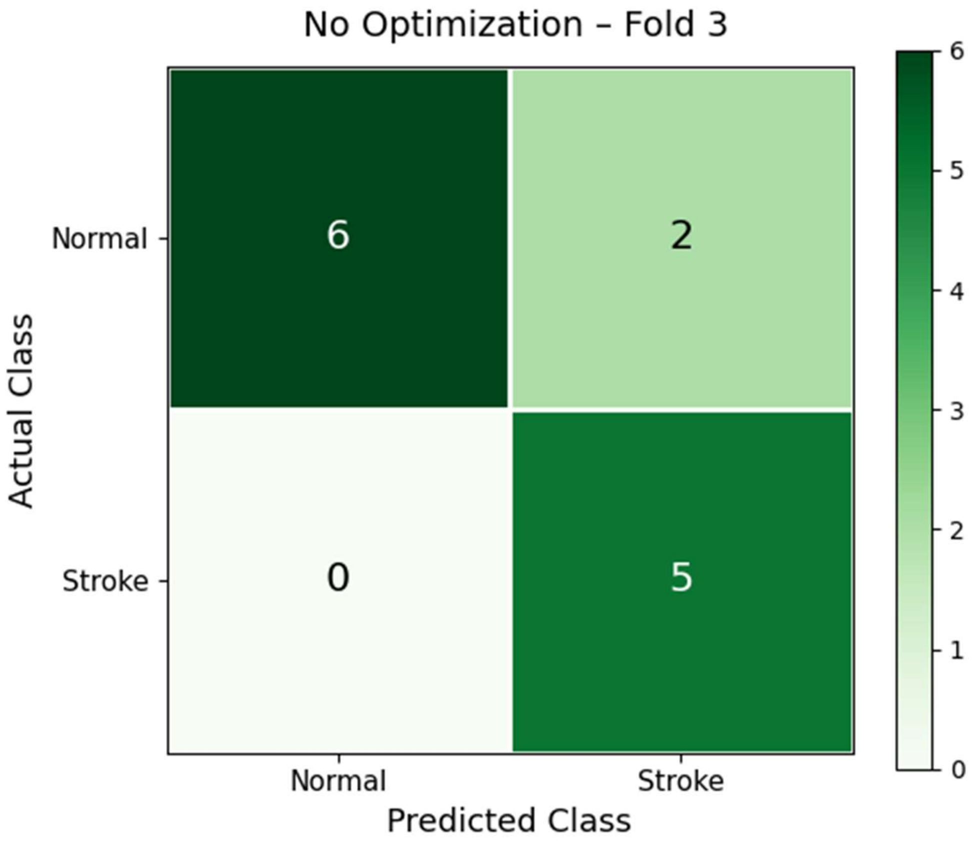
No Optimization – Fold 3
Actual Class
Normal
Stroke
6
2
0
5
Predicted Class
Normal
Stroke
0
1
2
3
4
5
6

(c)

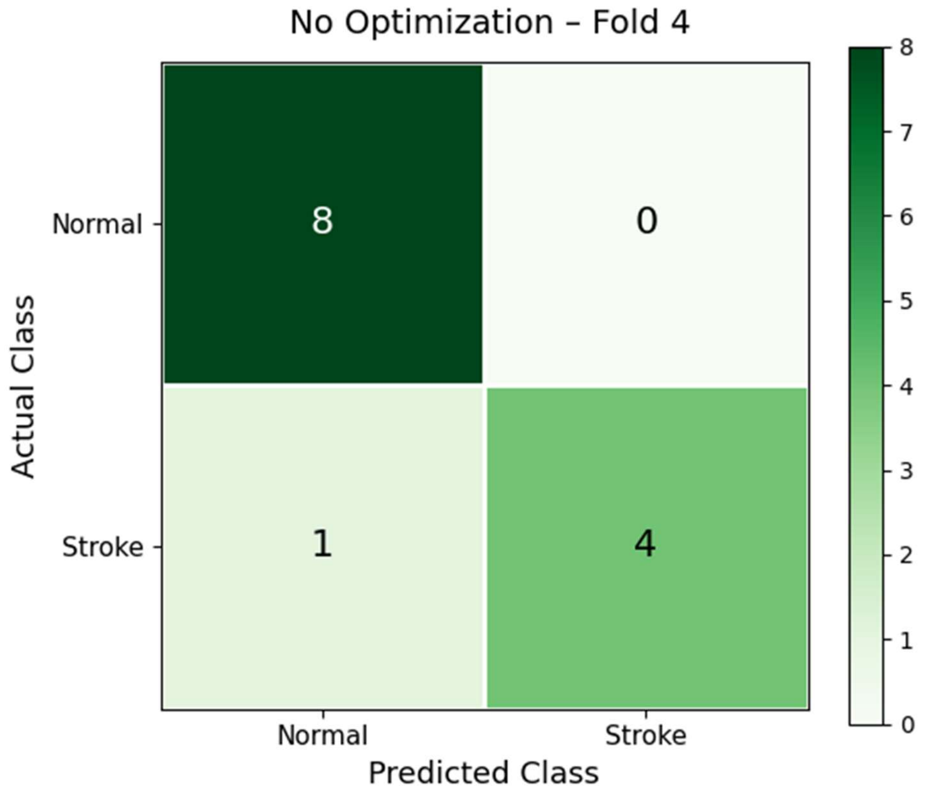
No Optimization – Fold 4
Actual Class
Normal
Stroke
8
0
1
4
Predicted Class
Normal
Stroke
0
1
2
3
4
5
6
7
8

(d)

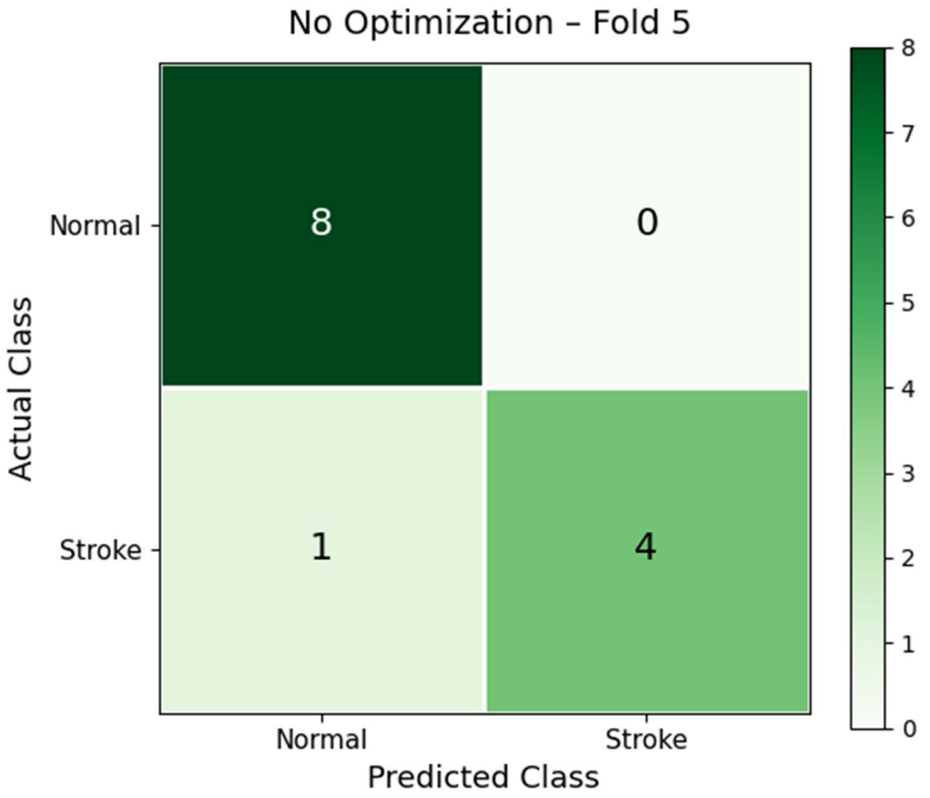

No Optimization – Fold 5
Normal
Stroke
8
0
1
4
Actual Class
Normal
Stroke
Predicted Class
8
7
6
5
4
3
2
1
0

(e)

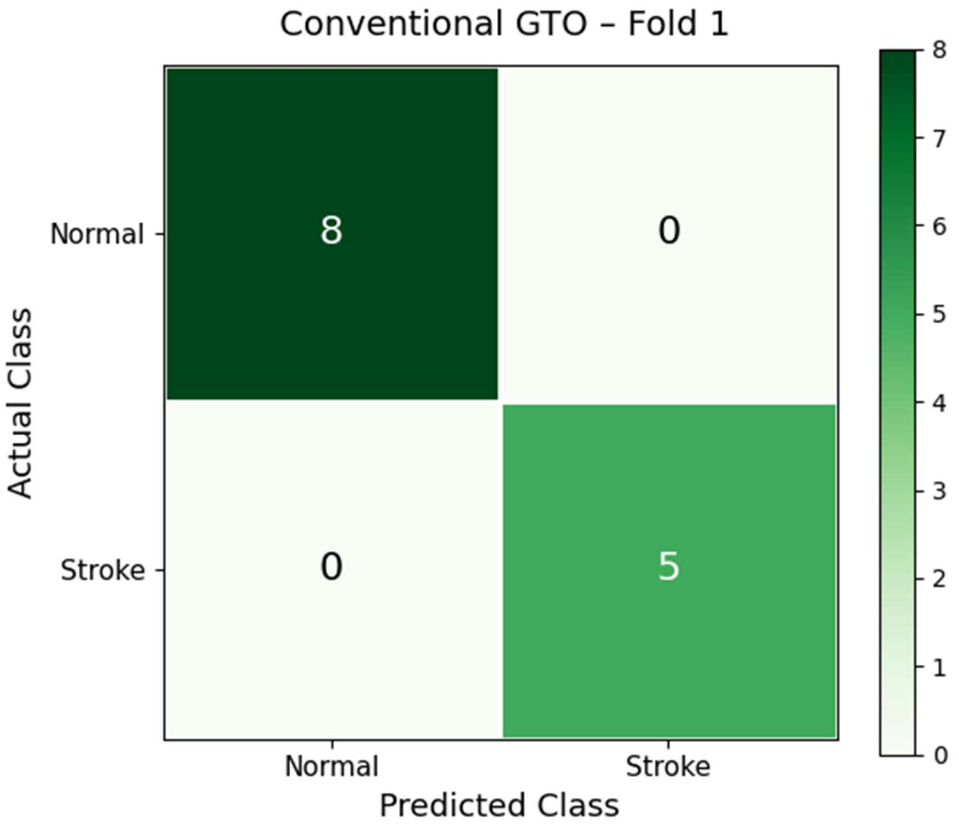

Conventional GTO – Fold 1
Normal
Stroke
8
0
0
5
Actual Class
Normal
Stroke
Predicted Class
8
7
6
5
4
3
2
1
0

(f)

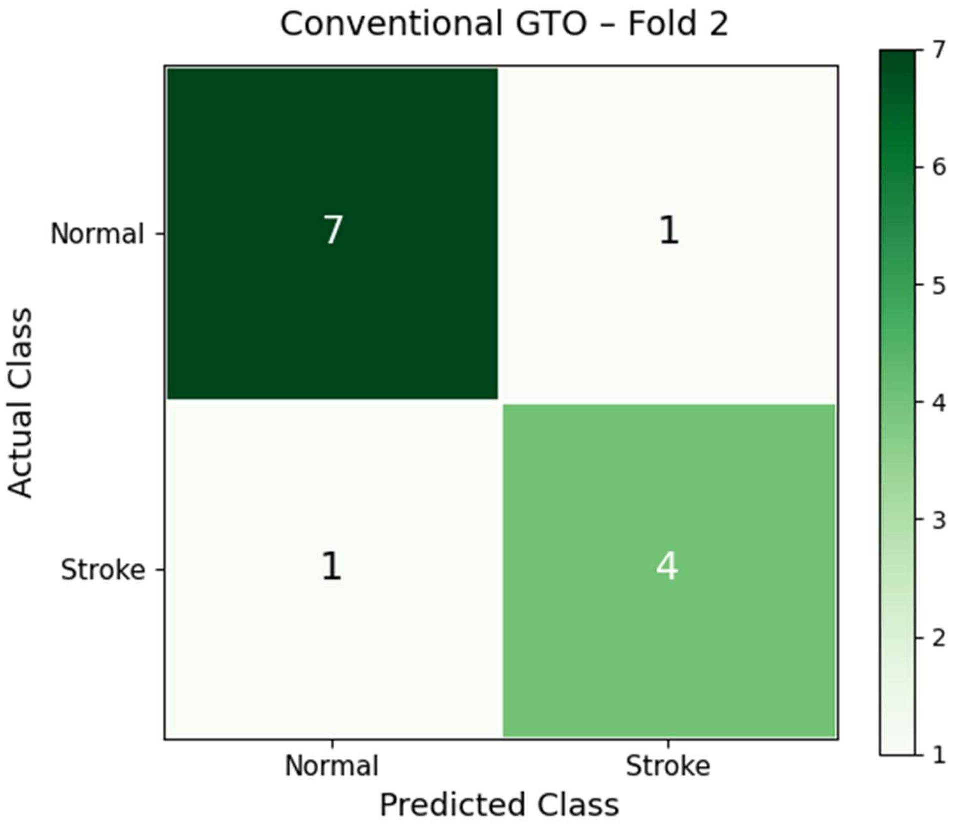
Conventional GTO – Fold 2
Normal
Stroke
Actual Class
Predicted Class
7
1
1
4
7
6
5
4
3
2
1

(g)

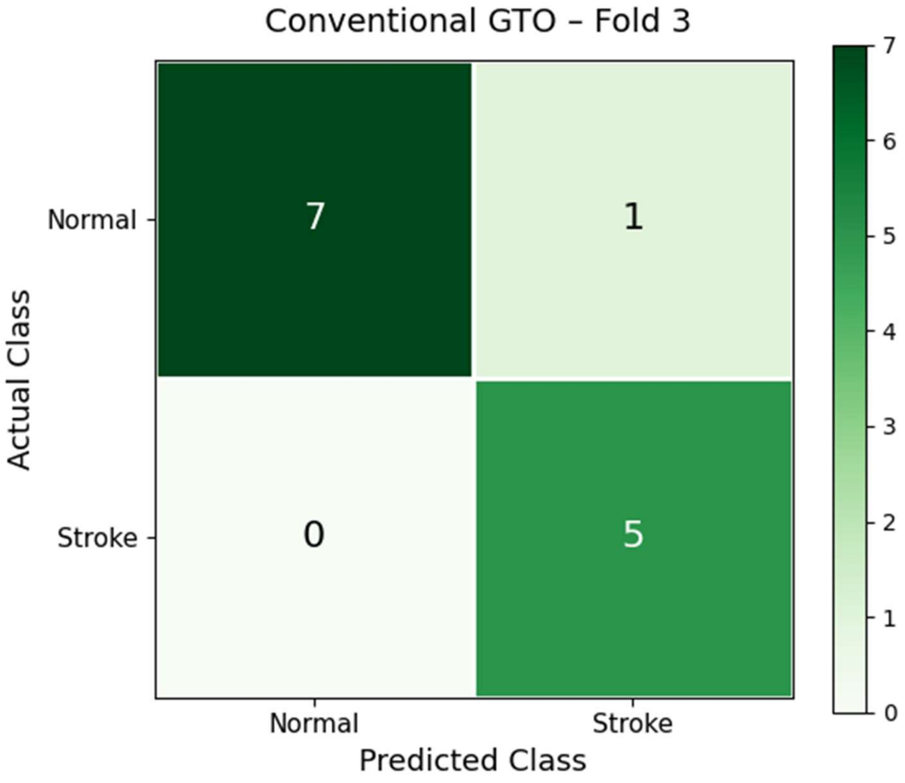
Conventional GTO – Fold 3
Normal
Stroke
Actual Class
Predicted Class
7
1
0
5
7
6
5
4
3
2
1
0

(h)

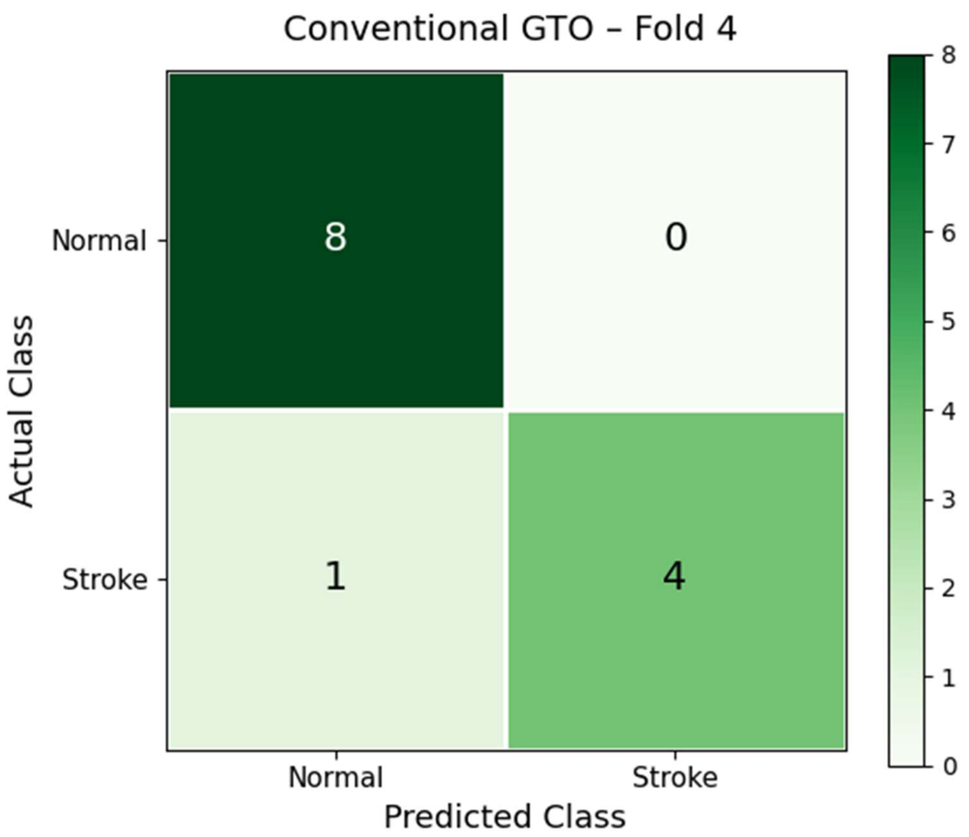
Conventional GTO – Fold 4
8
0
1
4
Normal
Stroke
Actual Class
Normal
Stroke
Predicted Class
0
1
2
3
4
5
6
7
8

(i)

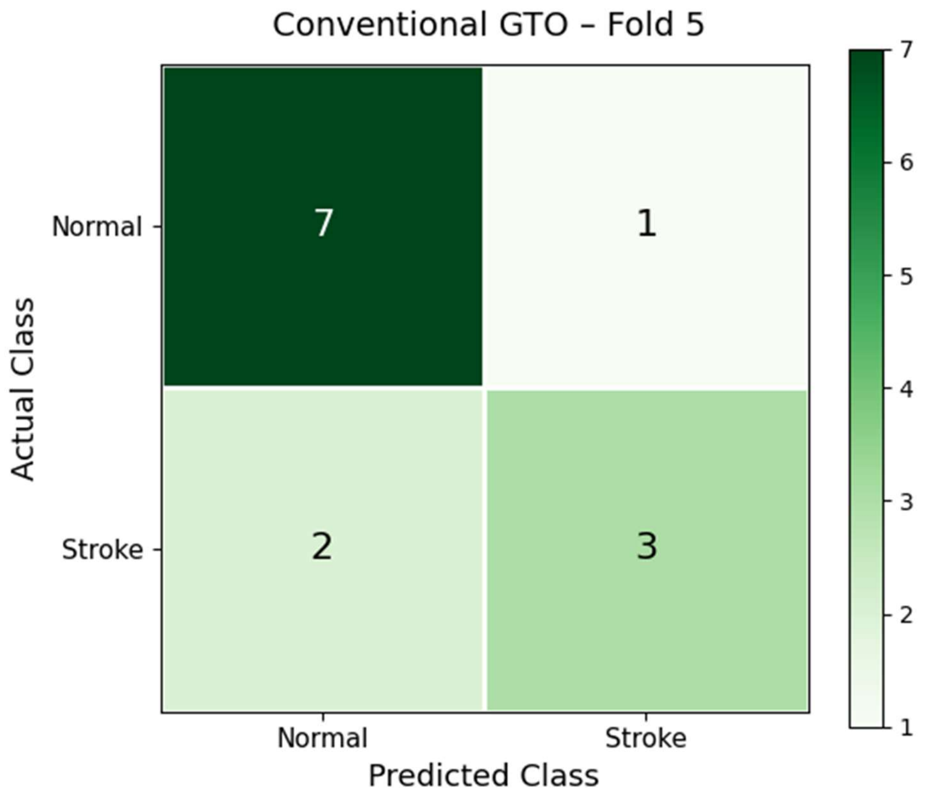
Conventional GTO – Fold 5
7
1
2
3
Normal
Stroke
Actual Class
Normal
Stroke
Predicted Class
1
2
3
4
5
6
7

(j)

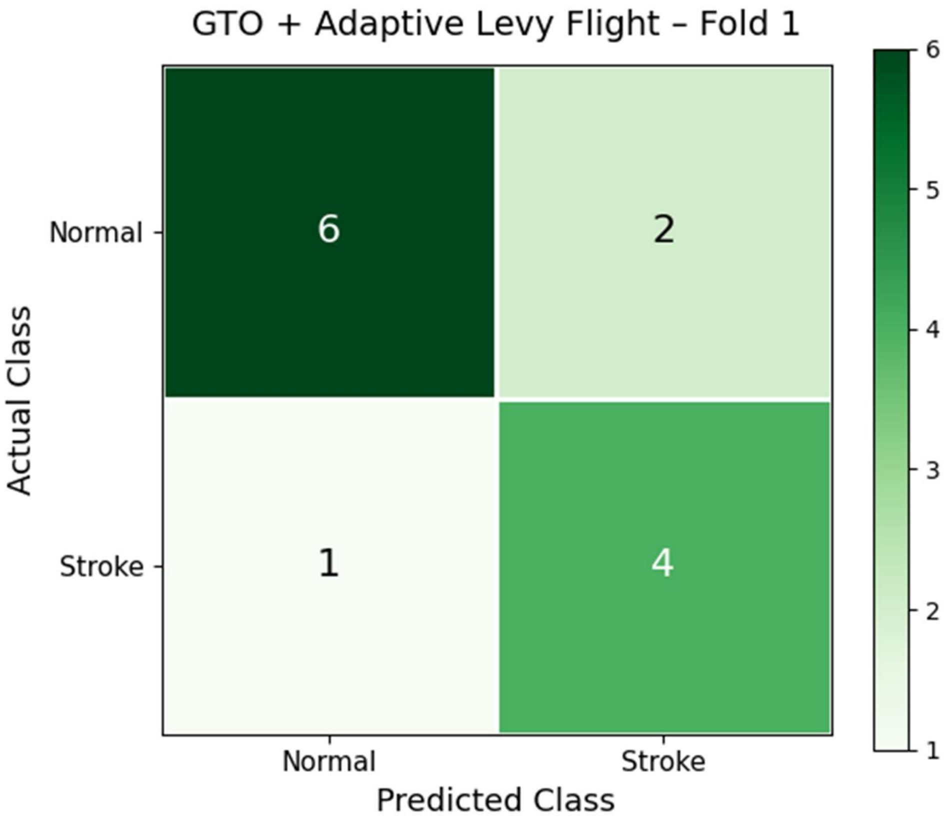
GTO + Adaptive Levy Flight – Fold 1
Actual Class
Normal
Stroke
6
2
1
4
Predicted Class
Normal
Stroke
6
5
4
3
2
1

(k)

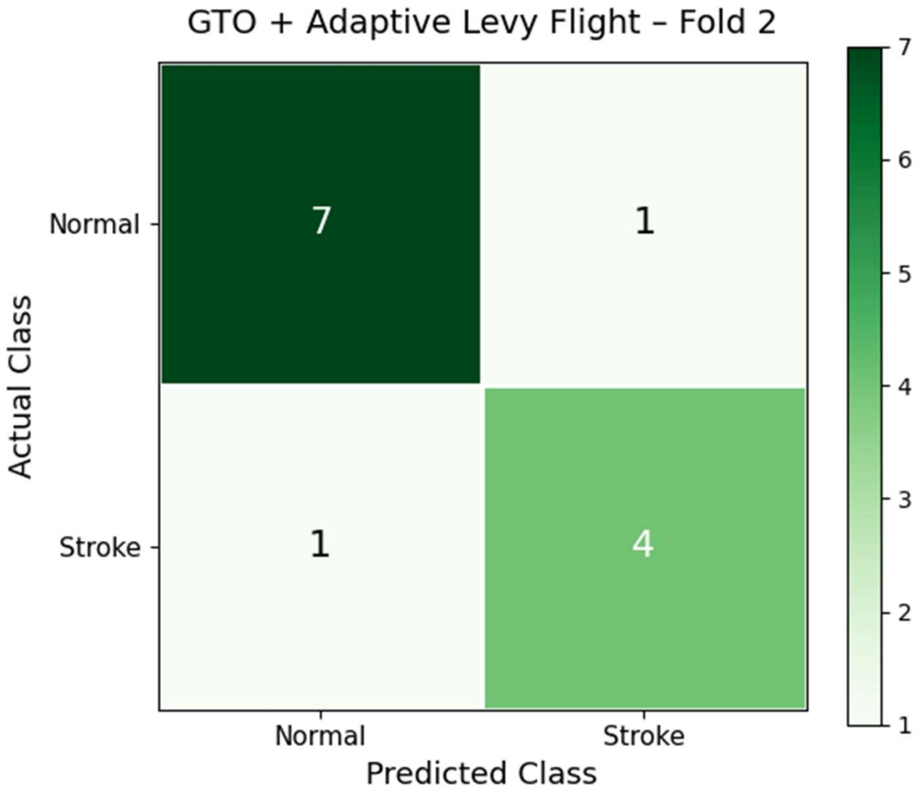
GTO + Adaptive Levy Flight – Fold 2
Actual Class
Normal
Stroke
7
1
1
4
Predicted Class
Normal
Stroke
7
6
5
4
3
2
1

(l)

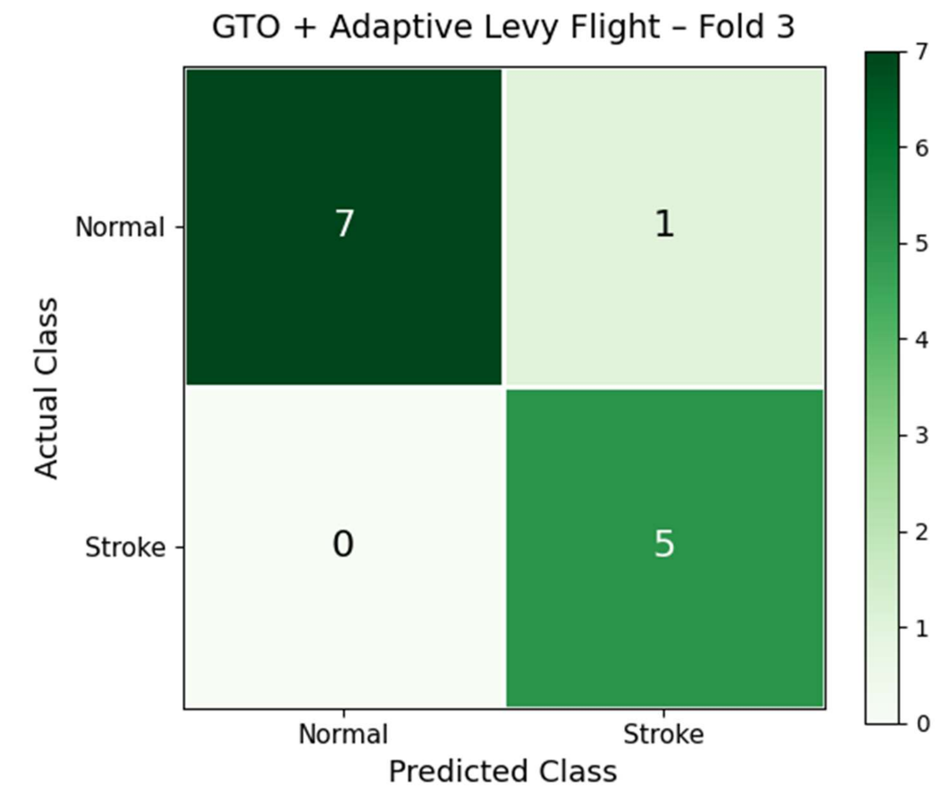
GTO + Adaptive Levy Flight – Fold 3
Actual Class
Normal
Stroke
7
1
0
5
Predicted Class
Normal
Stroke
0
1
2
3
4
5
6
7

(m)

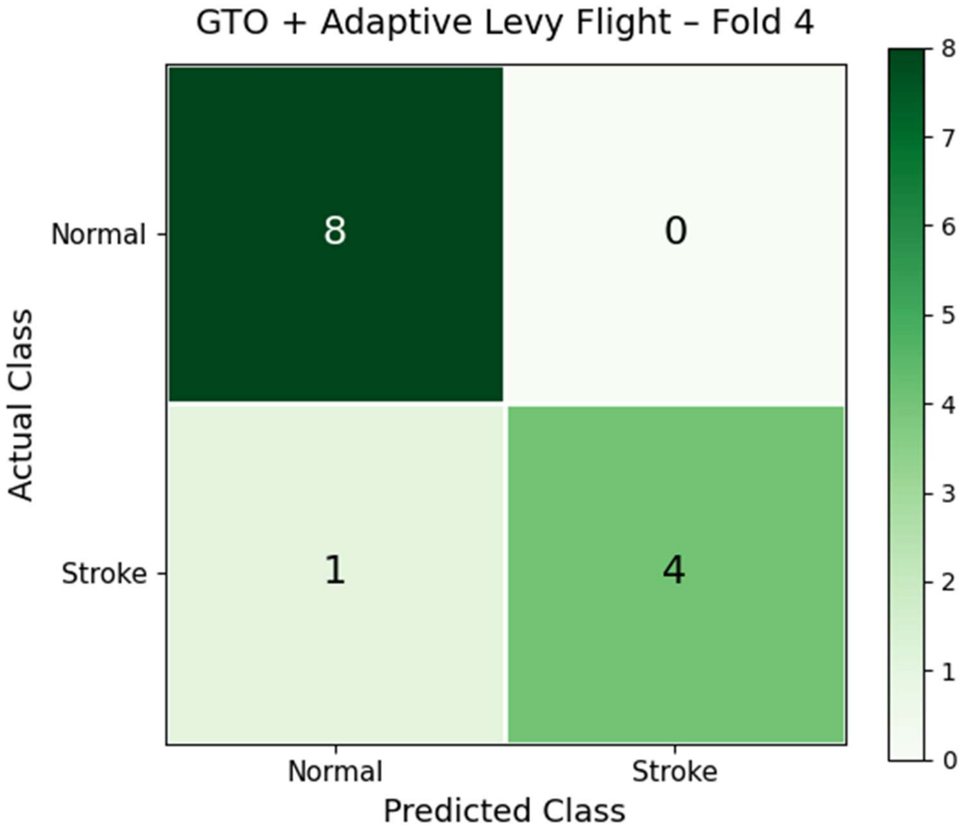
GTO + Adaptive Levy Flight – Fold 4
Actual Class
Normal
Stroke
8
0
1
4
Predicted Class
Normal
Stroke
0
1
2
3
4
5
6
7
8

(n)

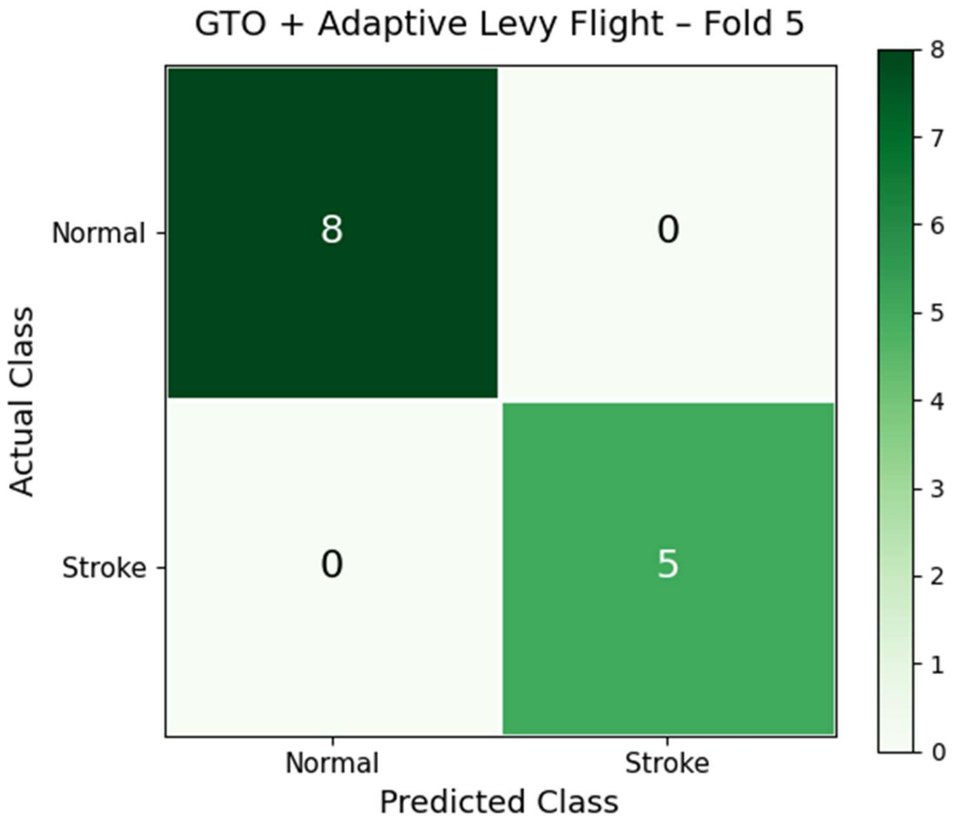
GTO + Adaptive Levy Flight – Fold 5
Normal
Stroke
Actual Class
8
0
0
5
Normal
Stroke
Predicted Class
8
7
6
5
4
3
2
1
0

(o)

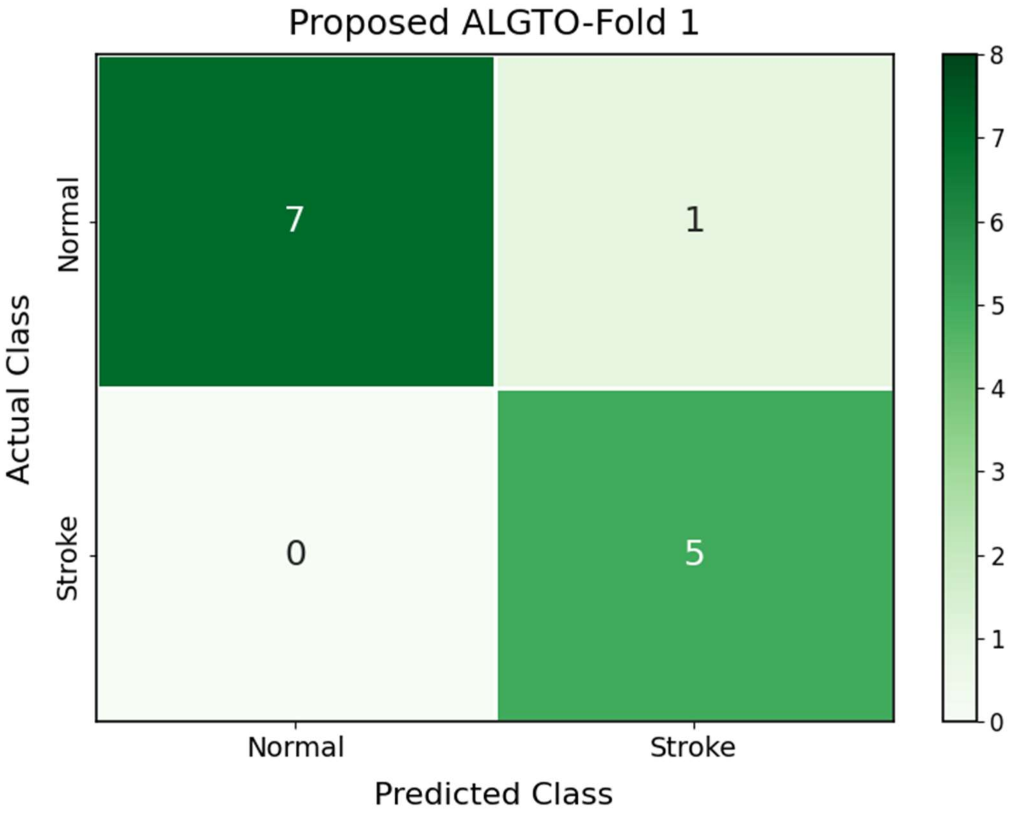
Proposed ALGTO-Fold 1
Normal
Stroke
Actual Class
7
1
0
5
Normal
Stroke
Predicted Class
8
7
6
5
4
3
2
1
0

(p)

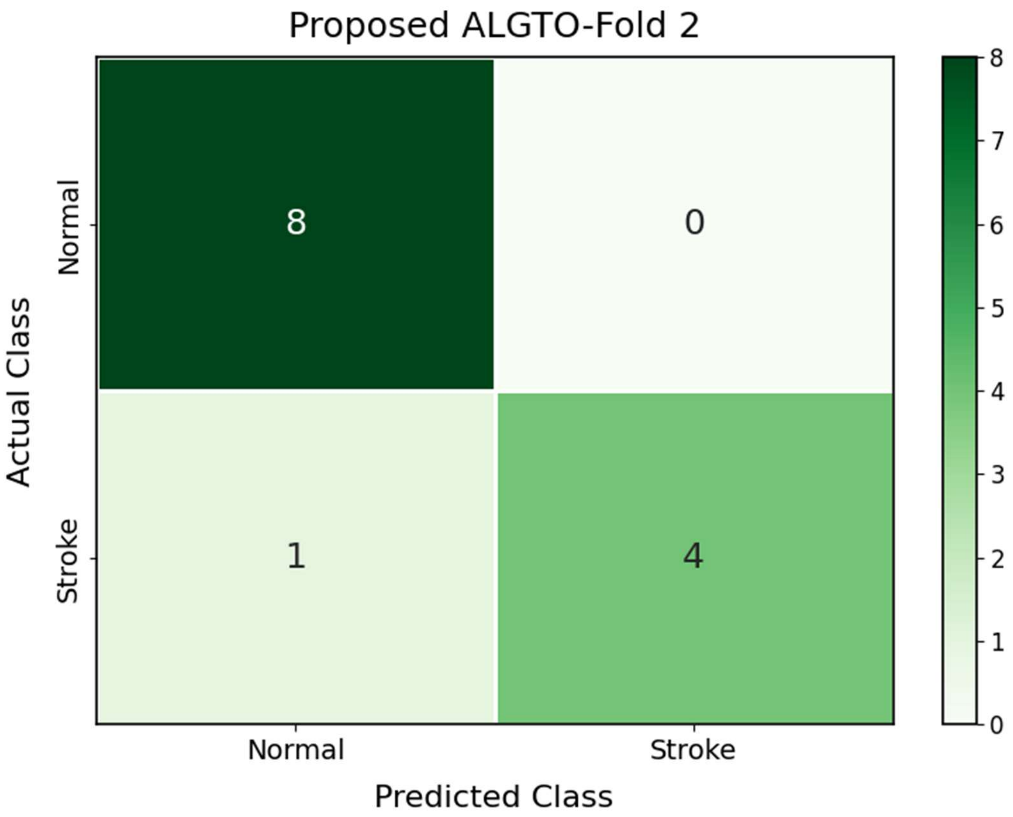
Proposed ALGTO-Fold 2
Actual Class
Normal
Stroke
8
0
1
4
Normal
Stroke
Predicted Class
0
1
2
3
4
5
6
7
8

(q)

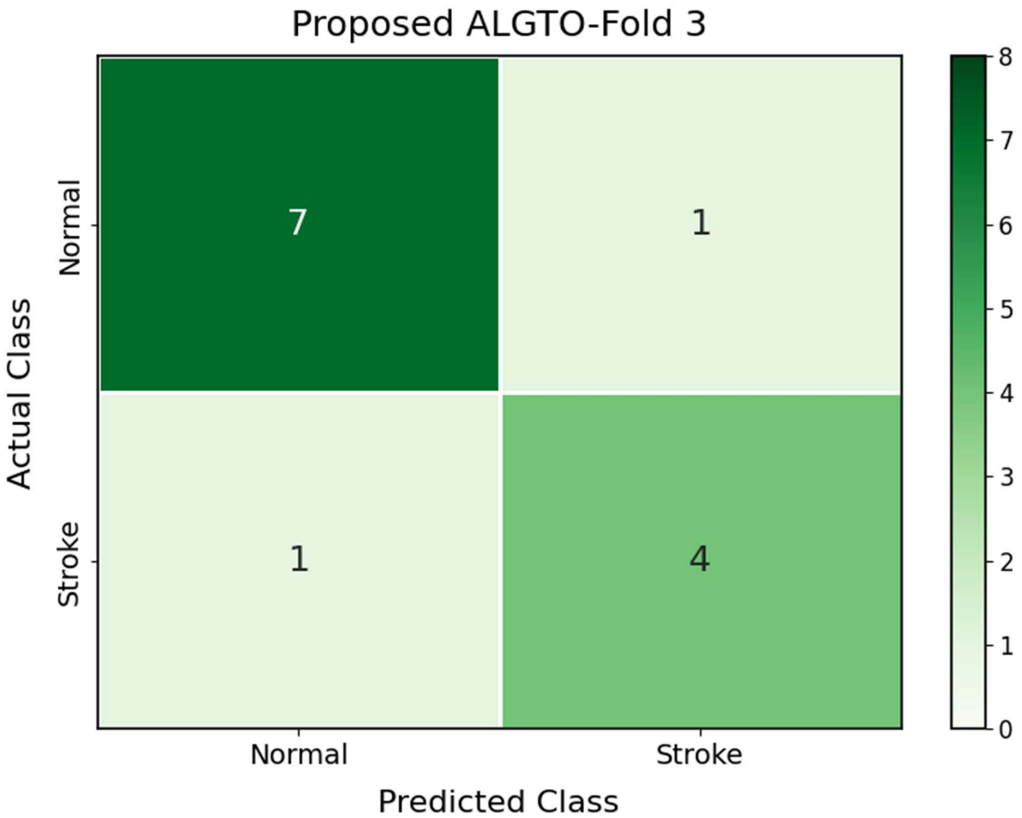
Proposed ALGTO-Fold 3
Actual Class
Normal
Stroke
7
1
1
4
Normal
Stroke
Predicted Class
0
1
2
3
4
5
6
7
8

(r)

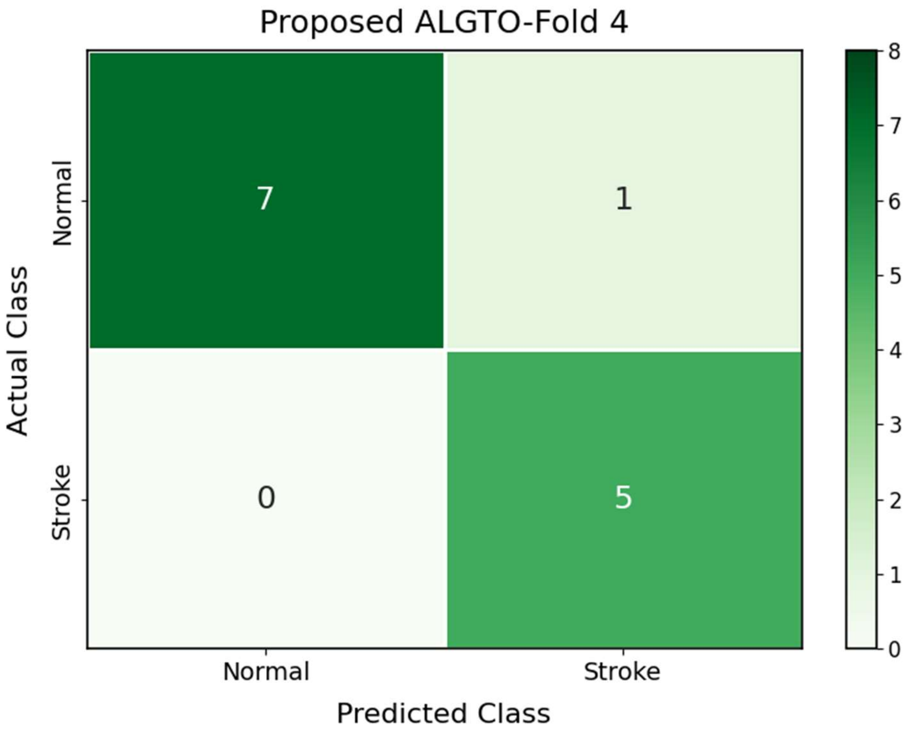

(s)

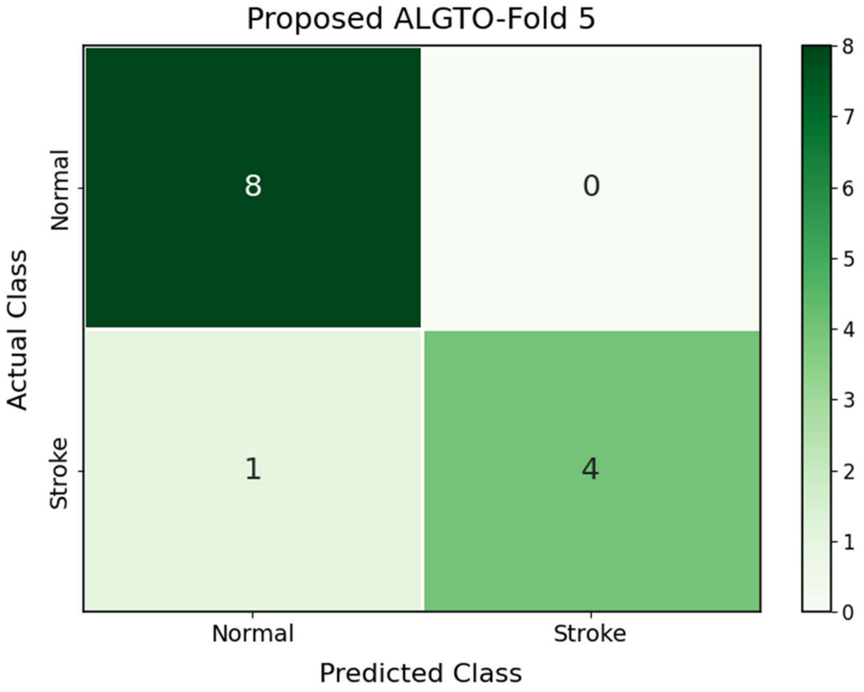

(t)

Figure S3. Fold-wise case-level confusion matrices of the optimization-based ablation across the five case-grouped cross-validation folds of the development set for the ConvNeXt-Tiny + Vision Mamba + AHDA + ACAF architecture: (a–e) no optimization, (f–j) conventional GTO, (k–o) GTO with adaptive Lévy flight, and (p–t) proposed ALGTO.
